# Health Effects of Plant-Based Diets in People with Overweight or Obesity: A Systematic Review and Meta-Analysis

**DOI:** 10.3390/nu18121987

**Published:** 2026-06-19

**Authors:** Ildikó Csölle, Viktória Cseh, Gábor Veres, László Czina, Daniela Kuellenberg de Gaudry, Dávid U. Nagy, Almut Georgi, Szimonetta Lohner

**Affiliations:** 1 Cochrane Hungary, Medical School, University of Pécs, 7624 Pécs, Hungary; csehviktoria92@gmail.com (V.C.); veres.gabor2@pte.hu (G.V.); laszlo.czina@pte.hu (L.C.); dkuellenberg@gmail.com (D.K.d.G.); davenagy9@gmail.com (D.U.N.); almut.georgi@gmx.de (A.G.); lohner.szimonetta@pte.hu (S.L.); 2 Doctoral School of Health Sciences, Faculty of Health Sciences, University of Pécs, 7621 Pécs, Hungary; 3 Institute of Biology/Geobotany and Botanical Garden, Martin Luther University Halle-Wittenberg, 06108 Halle, Germany; 4 Department of Public Health Medicine, Medical School, University of Pécs, 7624 Pécs, Hungary

**Keywords:** plant-based diets, obesity, health effect, systematic review and meta-analysis

## Abstract

Background/Objectives: Nutrition plays a core role in chronic disease management, and there is growing interest in the health impact of plant-based diets (PBDs) in people with overweight or obesity. We conducted this systematic review and meta-analysis to summarize the evidence on the health effect of PBDs compared to omnivorous diets in overweight or obese individuals. Methods: We searched the databases Cochrane Central Register of Controlled Trials, MEDLINE, Embase, ClinicalTrials.gov and WHO International Clinical Trials Registry Platform from inception to 3 January 2024. Two review authors independently screened studies for eligibility, extracted data, evaluated the risk of bias, and rated the certainty of the evidence using GRADE. This study is registered with PROSPERO, CRD42021225525. We used random-effects meta-analysis to analyze data. Results: Of 2664 records identified, 10 randomized controlled trials (RCTs) and six ongoing studies met the inclusion criteria. The available evidence suggests little to no difference between plant-based and omnivorous diets for body weight, systolic blood pressure, diastolic blood pressure, serum glucose, serum insulin, insulin sensitivity, total cholesterol, triglyceride, HDL cholesterol and body fat mass. Plant-based diets may slightly reduce LDL cholesterol. They may also reduce BMI and HbA1c, although the certainty of the evidence is very low. Longer-duration dietary interventions (14 weeks or more) showed greater improvements in BMI, LDL cholesterol and HbA1c. Conclusions: Plant-based diets may represent a dietary option for people with overweight or obesity and may support modest improvements in selected cardiometabolic outcomes, although the available evidence is limited and uncertain. Most outcomes showed little or no difference between PBDs and comparison diets, while the observed effects on BMI and HbA1c were supported by very low certainty evidence.

## 1. Introduction

For the past decades it has been known that obesity is an important threat to health, since it can lead to a higher mortality rate and a significant reduction in quality of life [1]. The World Health Organization (WHO) defines overweight and obesity for both children and adults [2]. The burden of overweight and obesity has increased over time in most countries [3,4,5]. This trend has also been reflected in age-specific analysis, including younger age groups [3,5,6]. In order to address the global obesity epidemic, lifestyle factors should be examined, with nutrition being one of the most important determinants [7]. As obesity-related disability-adjusted life years (DALYs) are predicted to remain a substantial contributor to the global burden of diseases in the coming years, effective prevention and management strategies remain important [8]. There is growing interest in PBDs because of their potential health benefits and their possible role in supporting dietary approaches to chronic disease prevention and management. Potential health benefits of plant-based dietary patterns have been documented over the past several decades including observational studies indicating associations between plant-based nutrition and favourable cardiovascular outcomes [9,10]. RCTs have also demonstrated the effects of plant-based or vegetarian diets on weight loss or body mass index (BMI) [11]. In addition to health-related reasons, there are other factors for people to prefer PBDs, including spiritual, religious, ethical, and environmental impacts (e.g., lower greenhouse gas emissions, reduced pressure on water resources, biodiversity, land preservation), ecological footprint reduction, political and animal welfare [12,13]. In general, the main categories of PBDs may include lacto-ovo-vegetarianism, lacto-vegetarianism, ovo-vegetarianism, veganism and raw veganism. There is currently no universally accepted definition of PBDs [14]. Common features in PBDs are the content of fruit, vegetables, mushrooms, wholemeal cereals, grains, pulses and oilseeds [13]. Storz et al. [14] suggest that an appropriate definition should be written to meet the need for consistency of content and comparability of use across clinical practice interventions. PBDs are often considered synonymous with healthy eating [15] and have been associated with a decreased risk of developing a variety of non-communicable diseases [16].

As for their qualitative characteristics, PBDs could differ from “normal diets” in their energy content, macronutrient composition (e.g., animal- or plant-origin protein source), micronutrient content, and the proportion of plant raw materials consumed (dietary fibre). These factors could each have an independent effect on health-related outcomes, which makes it difficult to determine which factor essentially influenced the observed health change. Additionally, various industrially produced and ultra-processed foods that can also be used in vegetarian or vegan diets have been shown to have a large impact on obesity, hypertension and cardiovascular diseases (CVDs) [17,18], mainly because of their high fat, sugar and salt content [19]. One of the beneficial health effects of PBDs may be weight loss, which is due to the increased intake of fibre, which reduces energy intake, calorie content and calorie density and induces a feeling of fullness and satiety [15].

In view of the growing interest in PBDs, it seems worthwhile to prepare an overview to summarize the evidence that may show the impact of PBDs on weight loss and, in this context, on reducing obesity as a global epidemic. A Cochrane review [20] and a protocol [21] on vegan dietary patterns have been reported in the literature, as well as a position statement based on systematic research by the ESPGHAN Nutrition Committee on vegan diets and nutritional status in infants, children, and adolescents [22], but a systematic review and meta-analysis of the effects of PBDs on people with overweight or obesity in a broader definition of PBDs is still needed. To the best of the authors’ knowledge, few systematic reviews, meta-analyses or Cochrane reviews have specifically focused on RCTs evaluating the effects of PBDs exclusively in people with overweight or obesity.

The aim of this systematic review was to synthesize evidence from RCTs on the health effects of PBDs compared to omnivorous diets in people with overweight or obesity.

## 2. Materials and Methods

This systematic review and meta-analysis were registered in PROSPERO (International Prospective Register of Systematic Reviews, identifier CRD42021225525 (https://www.crd.york.ac.uk/PROSPERO/view/CRD42021225525, accessed on 1 April 2026) and are reported in adherence with the updated PRISMA guidelines for reporting systematic reviews [23].

We searched the following electronic databases and trial registers from the inception of each database up to 3 January 2024, without restrictions on the language of publication or publication status: Ovid MEDLINE, Cochrane Central Register of Controlled Trials (CENTRAL), Embase (www.embase.com), ClinicalTrials.gov and WHO ICTRP (International Clinical Trials Registry Platform) (trialsearch.who.int). Details for all search strategies are available in Supplementary File S1. In addition to the electronic searches, we conducted manual backward citation searching to find other potentially suitable studies or additional publications. We checked the reference list of included studies and systematic reviews conducted on similar topics and contacted the authors of studies where we found missing information.

We included studies in the systematic review that met all the following criteria:

(i) Randomized controlled trials (RCTs), with a parallel or crossover design, including full-text publications, registered but unpublished trials, conference abstracts or posters, and unpublished data.
(ii) Participants: Individuals of any age and from any country with overweight or obesity (BMI equal to or above 25 kg/m^2^) who were otherwise healthy and without diagnosed diabetes, hypertension, or cancer. Only studies in which all participants met our predefined BMI inclusion criteria were included.
(iii) Any plant-based dietary intervention, such as vegetarian, lacto-ovo-vegetarian, lacto-vegetarian, ovo-vegetarian, or vegan. The interventions were based on participants who might have received the plant-based diet from the researchers or the instructions on how to follow the plant-based diet. In addition to those who consumed only plant foods (vegan), diets that included only milk and milk products (lacto-vegetarianism), eggs (ovo-vegetarianism), or both (lacto-ovo-vegetarianism) were allowed as part of the plant-based diet.
(iv) Comparator: An omnivorous diet, usual diet, or controlled meat-containing diet. Studies comparing two different types of PBDs were also eligible.
(v) Minimum duration of the intervention: ≥7 days.
(vi) Studies assessing any health-related outcomes were included. Our primary outcomes were (a) anthropometry (weight, BMI, body fat mass—not prespecified outcome); (b) blood pressure (BP) (systolic BP and diastolic BP); (c) lipid levels (total cholesterol, LDL cholesterol, HDL cholesterol, triglycerides); (d) diabetes-related outcomes (glucose levels, serum insulin, glycated hemoglobin (HbA1c), insulin sensitivity). Our secondary outcomes were (a) adverse effects (e.g., symptoms related to vitamin deficiencies); (b) health-related quality of life; (c) socio-economic effects; (d) incidence of non-communicable diseases; (e) outcomes related to mood; (f) outcomes related to behaviour; and (g) neurocognition.

The following studies were excluded:

(i) RCTs including patients with diagnosed diabetes, hypertension, or cancer disease.
(ii) Interventions with Mediterranean diet and its variants (e.g., the green Mediterranean diet), therapy-related diets that suggest modifications to certain macronutrients (e.g., the Dietary Approaches to Stop Hypertension (DASH) diet), and those kinds of intervention meals that did not cover the entire day (e.g., interventions that only requested a plant-based diet at breakfast) and those that provided exclusively education on the benefits of a plant-based diet. We also excluded combined interventions containing both diet and exercise, as well as other types of combined interventions (behavioural), or plant-based dietary interventions combined with another kind of dietary restriction (e.g., plant-based diet containing only gluten-free cereals).

### 2.1. Selection Process

Pairs of review authors (SL, IC, VC, DUN, AG) independently screened the title and abstract of every record retrieved by the literature searches to determine which trials should be assessed further. We performed the screening using Covidence^TM^ software [24]. We obtained the full texts of all potentially relevant records and screened them for eligibility. Any disagreements were resolved through consensus or by discussion with a third review author (SL). We have presented a PRISMA flow diagram to describe the process of trial selection [23].

### 2.2. Data Extraction and Data Synthesis

Data were extracted by two independent authors (IC, VC). The data collected was compared, and in case of discrepancies, a final decision was made by comparing the data with the original text. We extracted data using pilot-tested data extraction forms in Excel. We extracted the following data, if available. (a) Study information data: author(s) of the study, title of the study, published date of the study (day, month, year), country, research design, randomized participants, randomized intervention group, randomized control group, sample size of each group, number of participants lost to follow-up/withdrawn/dropouts. (b) Participants: mean age, age range, sex, BMI, inclusion criteria and exclusion criteria, analyzed intervention group, analyzed control group, duration of the intervention. (c) Type of intervention: name of the control group, name of the intervention group, duration, details on the intervention and comparison, and concomitant intervention. (d) Type of outcomes: primary and any other outcomes specified and collected.

### 2.3. Assessment of Study Quality

Risk of bias in RCTs was assessed using version 2.0 of the Cochrane ‘Risk of bias’ tool (RoB2) [25,26]. Three review authors (IC, VC, DKdG) independently assessed the risk of bias of each included trial. We resolved any disagreements by consensus. We assessed the following RoB domains: (1) bias arising from the randomisation process; (2) bias due to deviations from intended interventions; (3) bias due to missing outcome data; (4) bias in measurement of the outcome; (5) bias in selection of the reported result. We rated each domain as ‘low risk of bias’, ‘some concerns’ or ‘high risk of bias’.

### 2.4. Statistical Analysis

When studies were comparable in terms of the intervention, outcome, and study design, estimates from different studies were pooled using a random-effects meta-analysis. We used a random-effects model because it estimates the mean effect across a distribution of diet-specific effects rather than forcing all diets to share one fixed effect. During data preparation for synthesis, we collected the mean ± standard deviation (SD) values for the outcomes. In case the results were presented in other effect measures, we used the conversion methods that can be found in the *Cochrane Handbook for Systematic Reviews for Interventions* [27]. If the data were missing (e.g., the measure of variance) for the control or treatment groups, the study was excluded from the synthesis; we did not use any kind of imputation. For dichotomous data, we presented results as risk ratios (RRs) or odds ratios (ORs) with 95% confidence intervals (CIs). For continuous data, we used mean differences (MDs) or standardized mean differences (SMDs, where appropriate) with 95% CIs for studies measuring outcomes in the same way, and standardized mean differences (SMDs) with 95% CIs for studies measuring outcomes in a variety of ways.

### 2.5. Synthesis Methods

We used the R software (version 4.4.1) to perform statistical analysis and to create plots with the ‘meta’ and ‘metafor’ packages (version 8.3-0 and 5.0-1, respectively) [28]. We used an inverse variance mean difference analysis random effects model for parallel studies and a generic inverse variance analysis when parallel and crossover studies were combined in the synthesis. For crossover trials, reported end-of-intervention values were analyzed using the generic inverse variance method. The Sidik–Jonkman estimator with the Hartung–Knapp adjustment was used for between-study variance estimation and confidence interval calculation. The Sidik–Jonkman estimator with the Hartung–Knapp adjustment was prespecified for between-study variance estimation and confidence interval calculation, as this approach provides a conservative framework for random-effects meta-analysis when heterogeneity is expected and the number of available studies may be limited.

In case a study had more than one intervention or control arm, we combined the results of the relevant groups (as described in Section 6.5.2.10 of the *Cochrane Handbook* [27]) and compared the combined group with the corresponding comparison arm.

In the present publication, primary outcomes are reported in detail, while results for the secondary outcomes are listed in the Supplementary Files S1–S8.

### 2.6. Assessment of Heterogeneity

Methodological heterogeneity was assessed by examining risk of bias, while clinical heterogeneity was assessed by examining similarities and differences between studies regarding types of participants, interventions, and outcomes. We considered the size and direction of effect and used a standard χ^2^ test with a significance level of α = 0.1 and I^2^ statistic, quantifying inconsistency across trials, to assess the impact of heterogeneity on the meta-analysis. We explored heterogeneity by conducting pre-specified subgroup analyses for different diet types and for duration of diet.

### 2.7. Grading of Recommendations Assessment, Development, and Evaluation (Certainty of the Evidence)

We followed the GRADE approach to rate the certainty of evidence [29]. The certainty of evidence was downgraded because of the risk of bias, inconsistency, indirectness and imprecision across the included studies.

## 3. Results

### 3.1. Study Selection

**Figure 1:**
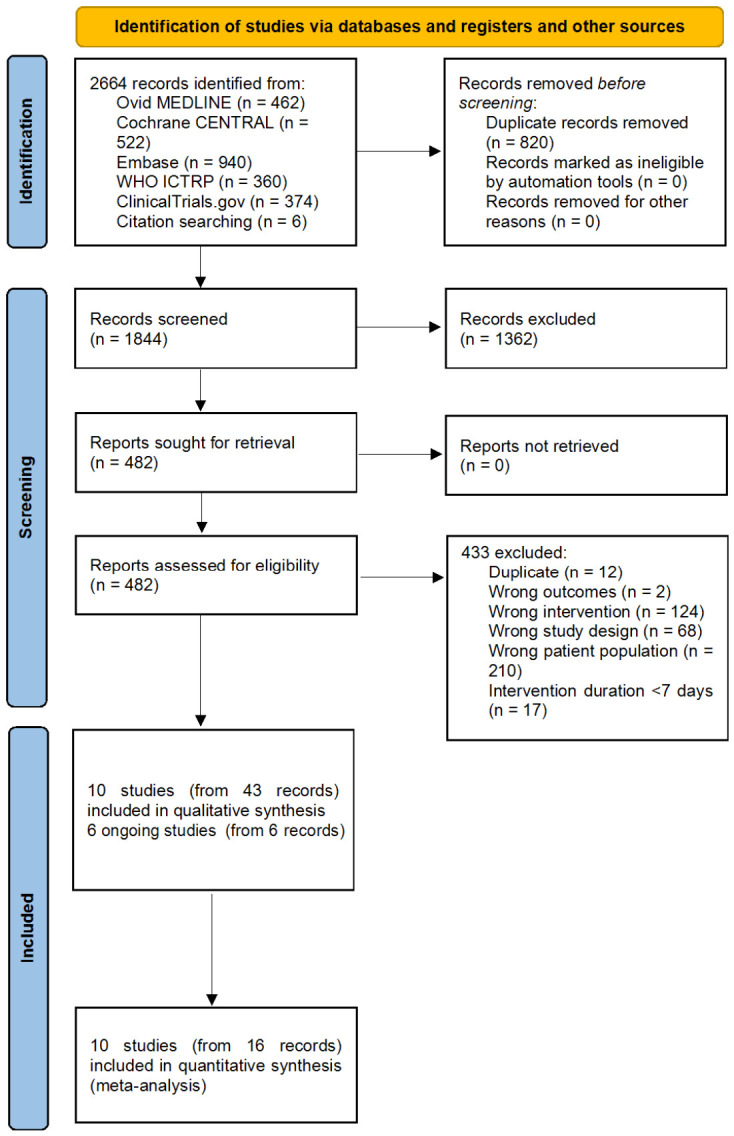
PRISMA flow diagram.

The search was run on 3 January 2024. We retrieved 2664 unique records through database searching, including six records through citation searching. After removing duplicates, 1844 records were screened based on their titles and abstracts. Most of the references clearly did not meet the inclusion criteria based on title and abstract review and were excluded. We evaluated 482 full texts or records to determine their eligibility for inclusion in the review. All articles were excluded after full-text assessment and the reasons for their exclusion are described in Supplementary File S2. Finally, ten studies met our inclusion criteria (Figure 1) [23].

### 3.2. Randomized Controlled Studies Characteristics

The main characteristics of the included studies are presented in Table 1. The detailed characteristics of included studies are presented in Supplementary File S3. Seven studies were parallel RCTs [30,31,32,33,34,35,36] and three had a crossover design [37,38,39]. All studies were conducted in high-income countries (USA, Canada, UK, Italy, Germany). Nine studies were conducted in adults aged between 15 and 75 years, while one study was conducted in mother-child pairs [33]. One study included only men [38], two studies included only women [30,36], and the remaining studies were conducted among both men and women [31,32,34,35,37,39]. In three studies the intervention was a low-fat vegan diet [35,36,37]; two studies reported on a modified low-fat vegan diet [33,34]. Furthermore, one study analyzed a low-carbohydrate vegan diet [31], another study investigated an energy-restricted high-protein lacto-ovo-vegetarian diet [32], and a further study reported on an energy-restricted lacto-ovo-vegetarian diet with different protein sources [30]. Additionally, one study investigated a soy-based high-protein weight-loss diet [38], and finally another study on a hypocaloric lacto-ovo-vegetarian diet [39]. Control groups received various diets, including the National Cholesterol Education Program diet [36], Mediterranean diet [37,39], American Heart Association diet [33], meat-based high-protein weight loss diet [38], energy-restricted lacto-ovo-vegetarian diet with different meat-based (chicken and beef) protein sources [30], and habitual diet [32,34,35]. There was one trial where a plant-based intervention diet was compared with another type of plant-based control diet, which was a high-carbohydrate lacto-ovo-vegetarian diet [31]. We categorized diets; these dietary categorizations have considered the differences between the macronutrients described in each diet named by the authors (Supplementary File S4).

### 3.3. Body Weight, Body Mass Index, Body Fat Mass

#### 3.3.1. Weight

**Figure 2:**
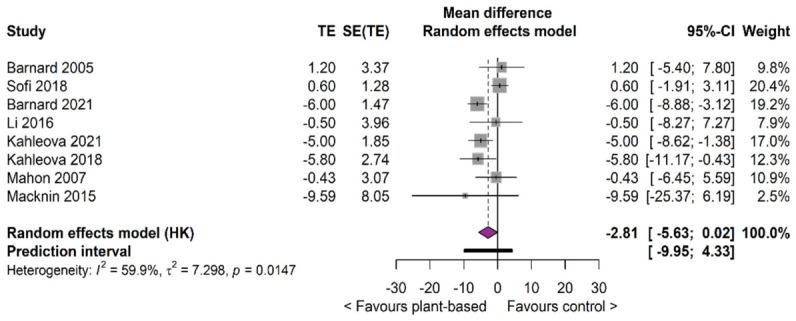
Plant-based diets versus control (omnivorous) diets. Outcome: body weight (kg). Abbreviations: TE treatment effect, SE (TE) standard error of treatment effect, HK Hartung–Knapp, CI confidence interval [30,32,33,34,35,36,37,39].

Eight RCTs—which compared plant-based intervention diets with an omnivorous control diet—measured body weight [30,32,33,34,35,36,37,39]. In these studies, there was no significant difference found in weight loss (MD: −2.81; 95% CI −5.63 to 0.02, *n* = 639, I^2^ = 59.9%, low certainty evidence; Figure 2 and Supplementary File S5, Analysis S1.1).

In one trial [31], conducted in participants who followed PBDs as an intervention diet and a lacto-ovo-vegetarian diet as a control diet, there was no difference in weight loss between the two groups (MD: −3.50; 95% CI −11.47 to 4.47, *n* = 50). Our subgroup analysis showed a significant difference between the low-fat vegan diet and the omnivorous diet in one trial [34] (MD: −5.80; 95% CI −11.17 to −0.43, *n* = 72; Supplementary File S5, Analysis S1.3). Subgroup analysis based on the duration of diet showed a significant effect of plant-based diet versus control diet in studies with 14 weeks or longer duration (MD: −5.64; 95% CI −7.02 to −4.26, 3 trials, *n* = 357; Supplementary File S5, Analysis S1.4). In trials shorter than 14 weeks there was no significant difference between the plant-based and the omnivorous diets [30,32,33,36,39] (MD: −0.07, 95% CI −2.92 to 2.79, 5 trials, 282 participants).

#### 3.3.2. BMI

**Figure 3:**
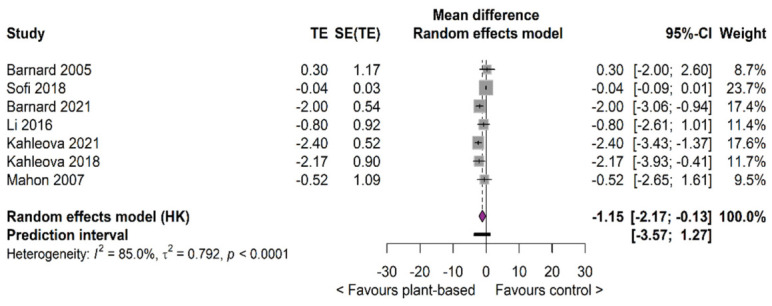
Plant-based diets versus control (omnivorous) diets. Outcome: BMI (kg/m^2^). Abbreviations: CI confidence interval, HK Hartung–Knapp, TE treatment effect, SE (TE) standard error of treatment effect [30,32,34,35,36,37,39].

Based on seven trials [30,32,34,35,36,37,39] there was a significant difference between the intervention and control diet (MD: −1.15; 95% CI −2.17 to −0.13, *n* = 611, I^2^ = 85.0%, very low certainty evidence; Figure 3 and Supplementary File S5, Analysis S2.1).

In one trial [31] comparing plant-based diet with lacto-ovo-vegetarian diet there was no difference in BMI (MD −0.50, 95% CI −2.41 to 1.41, 1 trial, 50 participants). In one trial [33] conducted in children BMI percentile and BMI z-score were measured and no significant difference was described (Supplementary File S5, Analysis S2.3). Subgroup analysis by diet type found PBDs being more effective in decreasing BMI compared with control diet (MD: −1.15; 95% CI −2.17 to −0.13; Supplementary File S5, Analysis S2.4) In subgroup analysis by duration of the diet 14 weeks long or longer diets were effective in decreasing BMI (MD: −2.20, 95% CI −2.76 to −1.64), while no significant effects were seen in shorter trials (Supplementary File S5, Analysis S2.5).

#### 3.3.3. Body Fat Mass

Six trials [30,32,34,35,36,37] measured body fat mass (kg) or body fat percent (%). The overall (combined) result showed no difference between PBDs compared to the control diet (MD: −0.93; 95% CI −3.47 to 1.62, *n* = 493, I^2^ = 64.5%, very low certainty evidence; Supplementary File S5, Analysis S3.3). In one trial [31] comparing a plant-based diet with a lacto-ovo vegetarian diet, no difference in body fat mass (%) (Supplementary File S5, Analysis S3.4). There was no significant difference in the effect on body fat mass between the two groups compared in any of the subgroup analyses (Supplementary File S5, Analyses S3.5–S3.10).

### 3.4. Blood Pressure

Systolic blood pressure and diastolic blood pressure were compared between PBDs and omnivorous diets in three trials [32,33,37], no difference between the groups was found either in effect on systolic blood pressure (MD: 3.32; 95% CI −0.38 to 7.03, 3 trials, *n* = 124, I^2^ = 0.0%, very low certainty evidence; Supplementary File S5, Analysis S4.1), or diastolic blood pressure (MD: 2.07; 95% CI −0.68 to 4.83, 3 trials, *n* = 124, I^2^ = 0.0%, very low certainty evidence; Supplementary File S5, Analysis S4.2) In one study [31], the control diet, which was another plant-based diet, was not found to be superior to the intervention diet in its effects on systolic and diastolic blood pressure (Supplementary File S5, Analyses S4.3 and S4.4). Subgroup analyses by diet type and duration of intervention did not find favourable changes in blood pressure levels between groups (Supplementary File S5, Analyses S4.5–S4.8).

### 3.5. Lipid Levels

#### 3.5.1. Total Cholesterol

In seven trials [30,32,33,35,37,38,39] no effect on total cholesterol levels was found between plant-based diet and omnivorous diet (MD: −4.31; 95% CI −18.88 to 10.27, *n* = 528, I^2^ = 51.8%, very low certainty evidence; Supplementary File S5, Analysis S5.1). In the trial [31] which compared plant-based diet with lacto-ovo-vegetarian diet, no significant difference was seen either (Supplementary File S5, Analysis S5.2). We found significant difference in none of the subgroups (Supplementary File S5, Analyses S5.3 and S5.4).

#### 3.5.2. LDL Cholesterol

In seven trials [30,32,33,35,37,38,39] we found significantly lower LDL levels in participants in the PBDs as compared to the omnivorous diet group (MD: −7.29; 95% CI −13.30 to −1.28, *n* = 528, I^2^ = 0.0%, low certainty evidence; Supplementary File S5, Analysis S5.5), but not between plant-based diet and lacto-ovo-vegetarian diet [31] (MD: −13.15; 95% CI −34.23 to 7.93, *n* = 50; Supplementary File S5, Analysis S5.6). When subgroup analysis by diet type and duration was performed, we found a significant difference between groups (MD −7.29; 95% CI −13.30 to −1.28, *n* = 528, I^2^ = 0.0%; Supplementary File S5, Analyses S5.7 and S5.8).

#### 3.5.3. HDL Cholesterol

Seven trials evaluated the effect of a plant-based diet on HDL-cholesterol [30,32,33,35,37,38,39]. Statistical analysis showed that participants following a plant-based diet did not demonstrate significant differences compared with those in the control group (MD: −0.09; 95% CI −4.73 to 4.92, *n* = 528, I^2^ = 62.8%, very low certainty evidence; Supplementary File S5, Analysis S5.9) and no difference was found in those who followed a lacto-ovo-vegetarian diet versus a plant-based diet [31] (Supplementary File S5, Analysis S5.10). In the diet duration subgroup analysis, no significant difference in HDL levels compared to the omnivorous diet (MD: 0.09; 95% CI −4.73 to 4.92, *n* = 528, I^2^ = 62.8%; Supplementary File S5, Analysis S5.11). In the diet type subgroup analysis, we found no significant differences in PBDs versus the omnivorous diet (Supplementary File S5, Analysis S5.12).

#### 3.5.4. Triglycerides

Six trials [32,33,35,37,38,39] reported data on triglyceride levels. There was no difference in triglyceride levels, when plant-based diet was compared with the control diet (MD: 3.21; 95% CI −4.45 to 10.87, *n* = 485, I^2^ = 0.0%, low certainty evidence; Supplementary File S5, Analysis S5.13) or when plant-based diet was compared with lacto-ovo-vegetarian diet (MD: −18.59; 95% CI −58.68 to 21.50, *n* = 50; Supplementary File S5, Analysis S5.14). Similarly, subgroup analysis based on types of diets and duration of the intervention identified no differences between groups (Supplementary File S5, Analyses S5.15 and S5.16).

### 3.6. Diabetes-Related Outcomes

#### 3.6.1. Glucose

In total, seven trials reported data on glucose levels [31,32,35,36,37,38,39]. No significant difference was shown between the plant-based diet and control groups in glucose concentrations (MD: −1.46; 95% CI −4.62 to 1.69; *n* = 552; I^2^ = 16.8%, low certainty evidence; Supplementary File S5, Analysis S6.1). One trial [31] showed no difference between a plant-based diet and a lacto-ovo vegetarian diet (MD: 0.00; 95% CI −4.85, 4.85; *n* = 50; Supplementary File S5, Analysis S6.2). Significantly lower glucose levels were found in the subgroup of trials [35,36,37] with a low-fat vegan diet as compared to a control diet (MD: −4.72; 95% CI −9.25 to −0.19; *n* = 344; I^2^ = 0%, 3 trials; Supplementary File S5, Analysis S6.3). There was no significant difference in glucose levels compared to the control diets in the duration of the diet subgroup analysis (Supplementary File S5, Analysis S6.4).

#### 3.6.2. Insulin

Five trials [32,33,35,36,39] reported data for insulin (MD: −3.46, 95% CI: −7.91 to 0.98, *n* = 434, I^2^ = 53.7%, low certainty evidence; Supplementary File S5, Analysis S6.5) plant-based diet compared to an omnivorous diet. In neither case was there a significant difference in the result of comparing a plant-based diet versus a lacto-ovo-vegetarian diet (Supplementary File S5, Analysis S6.6), or the duration of the diet subgroup analysis (Supplementary File S5, Analysis S6.8) nor in the results of the subgroup analysis of diet types (Supplementary File S5, Analysis S6.7).

#### 3.6.3. HbA1c

Three studies [33,35,37] reported data on HbA1c levels and showed a significant effect of a plant-based diet as compared to an omnivorous diet on HbA1c levels (MD: −0.11; 95% CI −0.21 to −0.02, *n* = 313, I^2^ = 0.0%, very low certainty evidence; Supplementary File S5, Analysis S6.9). There was no difference between a plant-based diet compared to a lacto-ovo-vegetarian diet on glycated hemoglobin (MD: 0.00; 95% CI −0.25 to 0.25, *n* = 50; Supplementary File S5, Analysis S6.10) [31]. Analysis stratified by subgroups (types of diet, duration of diet) also showed a significant difference between the plant-based diet and omnivorous diet (Supplementary File S5, Analyses S6.11 and S6.12).

#### 3.6.4. Insulin Sensitivity

Three trials measured insulin sensitivity [35,36,37]. There was no significant difference between omnivorous diet and PBDs (SMD: 0.26; 95% CI −0.04 to 0.57, *n* = 344, I^2^ = 0.0%, very low certainty evidence; Supplementary File S5, Analysis S6.13) and in the diet duration in the subgroup analysis (Supplementary File S5, Analysis S6.15), and in the diet types subgroup analysis (Supplementary File S5, Analysis S6.14).

See Supplementary File S6 for the pooled estimate table. The included studies did not provide sufficient data to allow analysis of socio-economic effects.

### 3.7. Results of Risk of Bias Assessment

Overall, five randomized controlled parallel trials (50%) (four parallel RCTs [31,32,34,35]; one crossover RCT [38]) were rated as having a high risk of bias due to bias in the randomization process and differences between groups in drop-out rates. Two studies were rated with overall some concern, also due to the randomization process (20%), two parallel RCTs [30,33], and three RCTs were assessed to be at low risk of bias (30%) (one parallel RCT [36], two crossover RCTs [37,39]). For an overview of review authors’ judgements about each ‘Risk of bias’ item for RCTs see Supplementary File S7. See in Supplementary File S8 the grading of recommendations assessment, development.

## 4. Discussion

In this systematic review and meta-analysis, we investigated the health effects of PBDs compared with control diets on anthropometric data, blood pressure, lipid levels, diabetes-related outcomes and additional outcomes in individuals with overweight or obesity. Our results suggest that PBDs may influence several health outcomes in individuals with overweight or obesity, including BMI, HbA1c and LDL cholesterol. Effects on BMI and LDL cholesterol were observed only in interventions of 14 weeks or longer, whereas shorter interventions showed little or no effect. Although BMI showed significant changes in some analyses, body weight outcomes did not reach statistical significance. This apparent discrepancy may partly reflect differences in the included studies and participants across analyses, as well as the substantial heterogeneity observed in the BMI analysis. Longer intervention periods may allow better dietary adherence and facilitate metabolic adaptations related to body composition, lipid metabolism, glycemic regulation and fibre-related effects on satiety and insulin sensitivity. Based on our subgroup analyses, intervention durations of at least 14 weeks may be more appropriate to detect clinically meaningful changes in BMI, LDL cholesterol and HbA1c, while improved standardization of dietary interventions and outcome reporting may enhance comparability across future studies.

For body weight, systolic blood pressure, diastolic blood pressure, serum glucose, serum insulin, total cholesterol, triglycerides, HDL cholesterol, and body fat mass, the available evidence suggests little to no difference between plant-based and omnivorous diets.

Regarding body weight, our findings differ from those of Tran et al. [13], whose systematic review reported benefits of plant-based intervention diets compared with control diets in eight of the 22 included studies, although no quantitative synthesis was conducted. Huang et al. [40] and Termannsen et al. [41] reported beneficial effects of vegan diets on weight-related outcomes.

In another systematic review and meta-analysis published in 2021, which evaluated weight status in subjects with obesity and T2DM, PBDs—mostly a vegan diet and in one case a lacto-ovo-vegetarian diet—led to reduced body weight compared to a meat-based diet [11]. In line with our findings, Tran et al. [13] also found significant reductions in BMI with plant-based intervention diets compared to control diets, consistent with findings of Termannsen et al. [41]. A non-randomized intervention trial evaluating an ad libitum low-fat plant-based diet supplemented with plant-based meal replacements (meat was allowed once a week) reported significant reductions in body fat [42], which is contrary to our findings. Several methodological differences between previous reviews and the present systematic review should be considered. Termannsen et al. [41] included studies up to 2022 and studied individuals with overweight or obesity who also had T2DM and a reduction in HbA1c was reported. Tran et al. [13] conducted a systematic review without a meta-analysis and limited their search to a single database (PubMed) up to 2019, using search terms alone. Their analysis focused on the outcomes of changes in body weight and/or BMI, and neither risk of bias assessment nor GRADE was reported in their publication. Huang et al. [40] only published a meta-analysis, which was an analysis of studies published up to 2014. The search of the publications was restricted by language (only English) and was collected by searching two databases (PubMed and Embase), but the search strategy was not reported. Huang et al. [40] examined a narrower range of intervention diets (vegan or lacto-ovo-vegetarian diets versus non-vegetarian diets) and used the Jadad score to assess the quality of the trials. Despite these methodological differences, our findings are consistent with those reported by Termannsen et al. [41], who identified no difference in blood pressure reduction (SBP and DBP) between participants following a vegan diet and those following a control diet. A multicentre prospective intervention trial compared nutritional changes associated with a low-fat vegan diet with those observed with a standard diet and examined whether a combined intervention programme conducted at a corporate site could reduce body weight and improve cardiovascular risk factors in individuals with overweight [43]. A plant-based diet promoted weight loss and had a higher fibre content than an omnivorous diet, but fibre intake was not a significant predictor of weight loss in Ferdowsian et al.’s [43] study. The effectiveness of vegan dietary patterns in preventing cardiovascular disease (primary and secondary) was summarized in a Cochrane review in 2021 [20]. The review concluded, in line with our results, that in studies with small numbers and few participants, the results were limited in terms of the different outcomes associated with the effect of a vegan diet intervention (with low-certainty evidence and moderate-certainty evidence). Studies examining vegan diets and vegetarian diets [41,44] have reported a reduction in total cholesterol levels, contrary to the results of our study. Xu et al. [44] conducted a systematic review and meta-analysis and searched three databases (Cochrane Library, Embase, MEDLINE) with language restrictions (English only) to identify RCTs comparing the effects of an intervention vegetarian diet and a control diet up to 2021. RoB summary was available for the RCTs assessed, but no published GRADE assessments were found. Participants also had a BMI of 25 kg/m^2^ or higher, and outcome measures included glucose and lipid profiles (TC, TG, HDL-C, LDL-C, fasting plasma glucose, HbA1c and HOMA-IR). Similar to our findings, Termannsen et al. [41] reported that the low-fat vegan diet reduced LDL cholesterol levels compared to the control diet, and we found similar results for HDL cholesterol and triglyceride levels. Similar to the analysis of Xu et al. [44], which compared overweight or obese subjects on a vegetarian diet, we also found significant differences in HbA1c parameters, while our analysis of subgroups showed no significant differences in glucose parameters. In a systematic review and meta-analysis conducted by Termannsen et al. [41], a decrease in HbA1c was observed in the subgroup analysis of individuals with overweight on a vegan diet, which showed a similar result for HbA1c compared to the present study. Regarding the results related to insulin, we did not observe favourable effects with the plant-based diet, which is contrary to the results reported by Termannsen et al. [45]. For markers of insulin sensitivity we found no significant difference between PBDs and control diets among participants with overweight or obesity, compared to the study [45] that found an improvement in insulin sensitivity on a low-fat vegan diet over a control diet in overweight adults. Another study comparing PBDs (with or without eggs) with control diets among participants with metabolic syndrome or other risk factors also reported no significant differences in insulin sensitivity [46]. These discrepancies may reflect differences in participant characteristics, co-morbidities, and intervention diets.

### Strengths and Limitations of the Review

A key strength of the present systematic review and meta-analysis is the comprehensive search strategy. We identified available RCTs without language restrictions by conducting a systematic search of three databases and two trial registers, and we did not restrict the outcomes eligible for inclusion. This strategy allowed us the broad assessment of the potential health effects of PBDs in individuals with overweight or obesity.

However, several limitations have to be considered when interpreting the results of this systematic review. First, the relatively low number of available studies limited our ability to properly assess the potential benefit of PBDs compared to a control diet. Only a few studies analyzed PBDs provided in the form of ready-to-eat meals in individuals with overweight or obesity, while a larger proportion of studies addressed intervention diets with modified macronutrient content, often accompanied by reduced energy intake. In some studies, the macronutrient composition of the control diet was not equivalent to the macronutrient content of the intervention diet, and the participants may have followed an energy-restricted diet aimed at weight reduction, which may have influenced the observed effects.

Another limitation is the heterogeneity of the intervention and control diets. The majority of evidence focused on low-fat vegan diets, as PBDs. Several of the primary studies used an omnivorous diet as the control diet. However, other diets were also used as control diets, and one study compared two PBDs (excluding meat). Therefore, the control diets were heterogeneous and there were too few studies for further subgroup analysis. This heterogeneity in the results highlights the role of certain modifying factors when evaluating the effects of PBDs in individuals with overweight or obesity, including differences in settings, types and definitions of PBDs, macronutrient intakes, protein sources and control diets. The substantial heterogeneity observed for several outcomes may also reflect differences in intervention duration, energy restriction, participant characteristics and comparator diet composition, which should be considered when interpreting the pooled estimates. Subgroup analysis based on diet type and intervention duration should therefore be interpreted cautiously, especially because of the low number of studies and small sample sizes for several outcomes (e.g., total cholesterol, HDL cholesterol, LDL cholesterol, glucose, insulin, insulin sensitivity).

We had planned to assess reporting bias using funnel plots and the Egger test when at least ten studies were available. However, due to the small number of studies, these analyses could not be performed, which is another limitation of the study.

In addition, we found limited evidence on several additional outcomes (adverse effect, adherence) that may be important to provide a more comprehensive picture of the benefits or harms of PBDs. Although PBDs are often associated with potential health benefits, the number of high-quality RCTs specifically conducted in individuals with overweight or obesity remains limited. Future RCTs investigating the effects of PBDs should follow a clearer and more consistent definition of PBDs and use rigorous methodology. Larger, well-designed RCTs with standardized dietary interventions and longer follow-up periods are needed to assess the health effects of PBDs in individuals with overweight and obesity.

## 5. Conclusions

The results of this systematic review and meta-analysis suggest that PBDs may represent a dietary option for people with overweight or obesity and may support modest improvements in selected cardiometabolic outcomes, although the available evidence is limited and uncertain. Most outcomes showed little or no difference between PBDs and comparison diets, while the observed effects on BMI and HbA1c were supported by very low-certainty evidence. Interpretation of these findings should consider the heterogeneity of the included dietary interventions, including differences in plant-based diet definitions, energy restriction, macronutrient composition, and comparator diets. Further well-designed RCTs with longer intervention durations are needed to strengthen the evidence base and clarify the potential health benefits of PBDs.

## Figures and Tables

**Table 1:** Summary of main characteristics of included studies.

Author, Year, Reference	Study Design/County	Sample Size (*n*) Analyzed Group Intervention/Control	Population	Duration of the Intervention	Intervention Diet	Control Diet	Measured Outcomes
Barnard 2005 [36]	parallel RCT/United States of America	59 (29/30)	overweight postmenopausal women, BMI 26–44 kg/m^2^	14 weeks	low-fat vegan diet	NCEP diet	weight, BMI, body fat %, GLU, insulin sensitivity
Barnard 2021 [37]	crossover RCT/United States of America	62 (62/62)	adults, BMI 28–40 kg/m^2^	16 weeks (36 weeks, 2 periods)	low-fat vegan diet	MED diet	weight, BMI, body fat (kg), SBP, DBP, GLU, Total COL, HDL, LDL, TRIG, HbA1c, insulin sensitivity
Jenkins 2014 [31]	parallel RCT/Canada	50 (20/19)	healthy men and postmenopausal women, BMI > 27 kg/m^2^	1 month + 6 months	low-carbohydrate PB diet	High-carbohydrate LOV diet	weight, BMI, body fat %, SBP, DBP, GLU, Total COL, HDL, LDL, TRIG, HbA1c
Kahleova 2021 [35]	parallel RCT/United States of America	223 (117/106)	female and male, BMI 28–40 kg/m^2^	16 weeks	low-fat vegan	Control	weight, BMI, body fat (kg), GLU, Total COL, HDL, LDL, TRIG, insulin, HbA1c, insulin sensitivity
Kahleova 2018 [34]	parallel RCT/United States of America	72 (35/37)	female and male, BMI 28–40 kg/m^2^	16 weeks	low-fat vegan	Control	weight, BMI, body fat (kg)
Neacsu 2014 [38]	crossover RCT/United Kingdom	20 (20/20)	overweight or obese men, BMI > 27 kg/m^2^	2 weeks	vegetarian HPWL (Soy-HPWL) soy protein or soy–textured vegetable protein	Meat-HPWL	weight, GLU, Total COL, HDL, LDL, TRIG
Li 2016 [32]	parallel RCT/United States of America	34 (17/17)	female and male, BMI 27.0–36.9 kg/m^2^	12 weeks	LOV diet energy-restricted diet	Control diet	weight, BMI, body fat %, SBP, DBP, GLU, Total COL, HDL, LDL, TRIG, insulin
Mahon 2007 [30]	parallel RCT/United States of America	43 (14/29)	not specified (postmenopausal women, BMI 29.6 ± 0.8 kg/m^2^ [BMI exclusion criteria < 25 and >34 kg/m^2^]	9 weeks	LOV diet, energy-restricted, different protein source	Beef diet plus chicken diet, energy restricted	weight, BMI, body fat %, GLU, Total COL, HDL, LDL, TRIG, insulin sensitivity
Sofi 2018 [39]	crossover RCT/Italy	118 (104/103)	overweight (body mass index [BMI] ≥ 25 kg/m^2^) and the simultaneous presence of ≥1 of the following criteria defined by the guidelines for cardiovascular disease prevention of the European Society of Cardiology:15 total cholesterol levels > 190 mg/dL, low-density lipoprotein (LDL) cholesterol levels > 115 mg/dL, triglyceride levels > 150 mg/dL, and glucose levels > 110 but <126 mg/dL	12 weeks	LOV diet energy restricted	MED Diet	weight, BMI, GLU, Total COL, TRIG, LDL, HDL, insulin
Macknin 2015 [33]	prospective RCT/United States of America	28 (14/14)	obese, hypercholesterolemic children, BMI > 95% and total cholesterol > 169 mg/dL	4 weeks	PB no added fat diet	AHA Diet	Children: BMI percentile, BMI z-score, SBP, DBP, weight, waist circumference, mid-arm circumference, PAQ, CHOL, TRIG, HDL-C, LDL-C, GLU, hsCRP, AST, ALT, IL-6, MPO, HbA1c, insulin

Abbreviations: AHA diet = American Heart Association Diet; ALT = Alanine aminotranferase; AST = Aspartate aminotransferase; BMI = Body Mass Index; CHOL = Cholesterol; DBP = Diastolic Blood Pressure; GLU = Glucose; HbA1c = Hemoglobin A1c; HDL-C = High-density lipoprotein cholesterol; HPWL = High-Protein Weight Loss Diet; hsCRP = High-sensitivity C-reactive protein; IL-6 = Interleukin-6; LDL-C = Low-density lipoprotein cholesterol; LOV = Lacto-ovo vegetarian; MED = Mediterranean Diet; MPO = Myeloperoxidase; NCEP = National Cholesterol Education Program Step II diet; PAQ = Physical Activity Questionnaire; PB = Plant-based; SBP = Systolic Blood Pressure; TRIG = Triglyceride; RCT = Randomized Controlled Trial.

## Data Availability

The raw data supporting the conclusions of this article will be made available by the authors on request.

## Supplementary Materials

The following supporting information can be downloaded at: https://www.mdpi.com/article/10.3390/nu18121987/s1, Supplementary File S1: Search strategies; Supplementary File S2: Characteristics of excluded studies; Supplementary File S3: Characteristics of included studies; Supplementary File S4: Nutrition table; Supplementary File S5: Analyses; Supplementary File S6: Pooled estimated table; Supplementary File S7: RoB; Supplementary File S8: GRADE.

## Abbreviations

The following abbreviations are used in this manuscript:

BMI	Body mass index
BP	Blood pressure
CI	Confidence interval
CVD	Cardiovascular disease
DALYs	Disability-adjusted life years
DASH	Dietary Approaches to Stop Hypertension
DBP	Diastolic Blood Pressure
GRADE	Grading of Recommendations Assessment Development and Evaluation
ICTRP	International Clinical Trials Registry Platform
HDL	High-density lipoprotein
HbA1c	Glycated hemoglobin
HOMA-IR	Homeostatic Model Assessment of Insulin Resistance
LDL	Low-density lipoprotein
MD	Mean difference
OR	Odds ratio
PBDs	Plant-based diets
PRISMA	Preferred Reporting Items for Systematic Reviews and Meta-Analysis
PROSPERO	International Prospective Register of Systematic Reviews
RoB	Risk of bias
RCT	Randomized controlled trial
RR	Risk ratio
SBP	Systolic Blood Pressure
SD	Standard deviation
SMD	Standardized mean difference
T2DM	Type 2 diabetes mellitus
TC	Total cholesterol
WHO	World Health Organization
