# Supplementary material for: Health Effects of Plant-Based Diets in People with Overweight or Obesity: A Systematic Review and Meta-Analysis
Source: Nutrients. 2026 Jun 19;18(12):1987. doi: 10.3390/nu18121987 (PMC13304861; doi:10.3390/nu18121987)
Supplement: Supplementary file 1 [file nutrients-18-01987-s001.zip › Supplementary File S1_Search strategies.pdf]

Supplementary file S1: Search strategies

Medline (Ovid)

1 obes*.tw.
2 adipos*.tw.
3 overweight*.tw.
4 "over weight*".tw.
5 (weight adj3 (reduc* or los* or maint* or decreas* or control* or gain* or change* or improve* or modif* or exessiv*)).tw.
6 fat.tw.
7 Obesity/
8 Obesity, Abdominal/
9 Obesity, Morbid/
10 Adiposity/
11 Body Weight Changes/
12 Body Weight/
13 Weight Loss/
14 Overweight/
15 or/1-14
16 vegan*.tw.
17 vegetarian*.tw.
18 exp Diet, Vegetarian/
19 exp Diet, Vegan/
20 exp Vegetarians/
21 exp Vegans/
22 ((plant-based or "plant based" or plantbased) adj3 (diet* or food*)).tw.
23 abstain*.tw.
24 herbivor*.tw.
25 veggie*.tw.
26 plant-eat*.tw.
27 (meatless or (meat adj2 free) or "no-meat" or "without meat").tw.
28 or/16-27
29 15 and 28
30 randomized controlled trial.pt.
31 controlled clinical trial.pt.
32 randomized.ab.
33 placebo.ab.
34 clinical trials as topic.sh.
35 randomly.ab.
36 trial.ti.
37 or/30-36
38 exp animals/ not humans.sh.
39 37 not 38

40 29 and 39

Cochrane Central Register of Controlled Trials (CENTRAL)

1 obes*:ti,ab

2 adipos*:ti,ab

3 overweight*:ti,ab

4 "over weight":ti,ab

5 weight NEAR (reduc* OR los* OR maint* OR decreas* OR control* OR gain* OR change OR improve OR modif* OR exessiv*):ti,ab

6 (body NEAR fat*):ti,ab

7 MeSH descriptor: [Obesity] this term only

8 MeSH descriptor: [Obesity, Abdominal] this term only

9 MeSH descriptor: [Obesity, Morbid] this term only

10 MeSH descriptor: [Overweight] this term only

11 MeSH descriptor: [Adiposity] explode all trees

12 MeSH descriptor: [Weight Gain] this term only

13 MeSH descriptor: [Body Weight] this term only

14 MeSH descriptor: [Weight Loss] this term only

15 #1 OR #2 OR #3 OR #4 OR #5 OR #6 OR #7 OR #10 OR #11 OR #12 OR #13 OR #14

16 vegan*:ti,ab

17 vegetarian*:ti,ab

18 MeSH descriptor: [Diet, Vegetarian] explode all trees

19 MeSH descriptor: [Diet, Vegan] explode all trees

20 ((plant-based OR "plant based" OR plantbased) NEAR (diet* OR food*)):ti,ab

21 abstain:ti,ab

22 herbivor:ti,ab

23 veggie*:ti,ab

24 (plant NEAR eat*):ti,ab

25 (meatless OR (meat NEAR free) OR "no-meat" OR "without meat"):ti,ab

26 #16 OR #17 OR #18 OR #19 OR #20 OR #21 OR #22 OR #23 OR #24 OR #25

27 #15 AND #26

Embase

1 obes*:ti,ab

2 adipos*:ti,ab

3 overweight*:ti,ab

4 'over weight*':ti,ab

5 (weight NEAR/3 (reduc* OR los* OR maint* OR decreas* OR control* OR gain* OR change* OR improve* OR modif* OR exessiv*)):ti,ab

6 fat:ti,ab

7 'obesity'/exp

8 'body weight change'/exp

9 #1 OR #2 OR #3 OR #4 OR #5 OR #6 OR #7 OR #8

10 vegan*:ti,ab

11 vegetarian*:ti,ab

12 'vegetarian diet'/exp

13 'vegan diet'/exp

14 'vegan'/exp

15 'vegetarian'/exp

16 (('plant based' OR 'plant based' OR plantbased) NEAR/3 (diet* OR food*)):ti,ab

17 abstain*:ti,ab

18 herbivor*:ti,ab

19 veggie*:ti,ab

20 'plant eat*':ti,ab

21 meatless:ti,ab OR ((meat NEAR/2 free):ti,ab) OR 'no meat':ti,ab OR 'without meat':ti,ab

22 #10 OR #11 OR #12 OR #13 OR #14 OR #15 OR #16 OR #17 OR #18 OR #19 OR #20 OR #21

23 #9 AND #22

24 'randomized controlled trial'/exp

25 'double blind procedure'/exp

26 'crossover procedure'/exp

27 'parallel design'/exp

28 'single blind procedure'/exp

29 random*:ti,ab

30 doubl* NEAR/1 blind*

31 singl* NEAR/1 blind*

32 assign*:ti,ab

33 allocat*:ti,ab

34 volunteer*:ti,ab

35 placebo*:ti,ab

36 factorial*:ti,ab

37 crossover*:ti,ab

38 'cross over':ti,ab

39 #24 OR #25 OR #26 OR #27 OR #28 OR #29 OR #30 OR #31 OR #32 OR #33 OR #34 OR #35 OR #36 OR #37 OR #38

40 #23 AND #39

WHO ICTRP (Standard search)

vegan

vegetarian

plant-based diet

plant-based food

meatless

meat-free

ClinicalTrials.gov (Expert search)

(obesity OR obese OR overweight OR adiposity OR body weight OR fat) AND (vegan OR vegetarian OR vegans OR vegetarians OR plant-based OR meatless OR „without meat" OR „meat-free")
