# Supplementary material for: Health Effects of Plant-Based Diets in People with Overweight or Obesity: A Systematic Review and Meta-Analysis
Source: Nutrients. 2026 Jun 19;18(12):1987. doi: 10.3390/nu18121987 (PMC13304861; doi:10.3390/nu18121987)
Supplement: Supplementary file 1 [file nutrients-18-01987-s001.zip › Supplementary File S2_Characteristics of excluded studies.pdf]

Supplementary file S2: Characteristics of excluded studies

Study	Reason for exclusion
Acharya 2013 [1]	Wrong intervention
Acharya 2009 [2]	Wrong intervention
Actrn 2021 [3]	Wrong intervention
Actrn 2016 [4]	Wrong patient population
Actrn 2007 [5]	Wrong intervention
ACTRN12607000394448 2007[6]	Wrong patient population
ACTRN12611000295943 2011 [7]	Wrong patient population
ACTRN12614000395639 2014 [8]	Wrong patient population
ACTRN12617000541303 2017 [9]	Wrong patient population
ACTRN12618001369213 2018 [10]	Wrong study design
ACTRN12620001151921 2020 [11]	Wrong patient population
ACTRN12621000743864 2021 [12]	Wrong study design
ACTRN12623000996662 2023 [13]	Wrong study design
Aldubayan 2023 [14]	Wrong intervention
Arntzenius 1986 [15]	Wrong patient population
Arora 1986 [16]	Wrong study design
Baden 2019 [17]	Wrong patient population
Ball 1997 [18]	Wrong study design
BarbaraJakšes 2011 [19]	Wrong study design
Barnard 2007 [20]	Wrong patient population
Barnard 2020 [21]	Duplicate

Barnard 2015 [22]	Wrong patient population
Barnard 2009 [23]	Wrong patient population
Barnard 2006 [24]	Wrong patient population
Barnard 2009 [25]	Wrong patient population
Barnard 2021 [26]	Wrong intervention
Barnard 2023 [27]	Wrong intervention
Barnard 2018 [28]	Wrong patient population
Barnard 2009 [29]	Wrong patient population
Barnard 2000 [30]	Wrong patient population
Barnard 2000 [31]	Wrong patient population
Barrett 2018 [32]	Wrong patient population
Beilin 1994 [33]	Wrong study design
Beilin 1987 [34]	Wrong study design
Beilin 1995 [35]	Wrong study design
Beilin 1988 [36]	Wrong study design
Belinova 2014 [37]	Intervention duration <7 days
Bendinelli 2023 [38]	Wrong intervention
Benson 2017 [39]	Wrong patient population
Bergeron 2019 [40]	Wrong patient population
Bernhart 2023 [41]	Wrong study design
BethIsraelDeaconessMedicalCenter 2019 [42]	Wrong patient population
Bhardwaj 2017 [43]	Wrong intervention
BoFeldt-Rasmussen 2020 [44]	Wrong patient population
Bouzas 2020 [45]	Wrong study design

Braden 2023 [46]	Intervention duration <7 days
Brestrich 1996 [47]	Wrong study design
Brooke 1980 [48]	Wrong patient population
Bunner 2014 [49]	Wrong patient population
Bunner 2014 [50]	Duplicate
Bunner 2015 [51]	Duplicate
Bunner 2015 [52]	Wrong patient population
Bunner 2014 [53]	Wrong patient population
Burke 2006 [54]	Wrong intervention
Burke 2007 [55]	Wrong intervention
Burke 2006 [56]	Wrong intervention
Burke 2008 [57]	Wrong intervention
Campbell 2022 [58]	Wrong patient population
Campbell 2023 [59]	Wrong patient population
Campbell 2023 [60]	Wrong patient population
Campbell 2022 [61]	Wrong patient population
Campbell 2023 [62]	Wrong patient population
Campbell 2023 [63]	Wrong patient population
Campbell 1999 [64]	Wrong intervention
ChiCTR1800019686 2018 [65]	Wrong patient population
ChiCTR2300078480 2023 [66]	Wrong patient population
ClevelandClinicFlorida 2019 [67]	Wrong patient population
Clinton 2015 [68]	Wrong patient population
Colombo 2005 [69]	Wrong patient population

Cooper 1982 [70]	Wrong patient population
Crimarco 2020[71]	Wrong intervention
Ctri 2023 [72]	Wrong study design
CTRI/2018/10/015896 2018 [73]	Wrong patient population
CTRI/2022/03/041032 2022 [74]	Wrong patient population
CTRI/2022/03/041033 2022 [75]	Wrong patient population
CTRI/2022/04/041626 2022 [76]	Wrong patient population
CTRI/2022/06/043292 2022 [77]	Wrong study design
CTRI/2022/09/045618 2022 [78]	Wrong patient population
CTRI/2023/06/053885 2023 [79]	Wrong study design
CTRI/2023/08/056621 2023 [80]	Wrong study design
Daniel-Gentry 1986 [81]	Wrong study design
Daubenmier 2006 [82]	Wrong patient population
DavidGrant 2022 [83]	Wrong study design
Davis 2019 [84]	Wrong patient population
deVries-Postma 2019 [85]	Wrong study design
DelRocioBerglund 2012 [86]	Wrong patient population
DiMauro 2021 [87]	Intervention duration <7 days
DiMauro 2020 [88]	Wrong intervention
Dogra 2020 [89]	Wrong patient population
Dressler 2022 [90]	Wrong patient population
DrexelUniversity 2020 [91]	Wrong study design
DRKS00004994 2013 [92]	Wrong study design
DRKS00010111 2016 [93]	Wrong study design

DRKS00012835 2018 [94]	Wrong patient population
DRKS00013702 2018 [95]	Wrong patient population
DRKS00015861 2019 [96]	Wrong patient population
DRKS00030940 2023 [97]	Wrong study design
DRKS00031541 2023 [98]	Wrong patient population
DRKS00031633 2023 [99]	Wrong patient population
DRKS00031713 2023 [100]	Wrong study design
Eberhard 2022 [101]	Wrong patient population
Eberhard 2022 [102]	Wrong patient population
Eberhard 2021 [103]	Wrong patient population
Elkan 2008 [104]	Wrong patient population
Elkoustaf 2017 [105]	Wrong intervention
Ellsworth 2016 [106]	Wrong study design
Ellsworth 2015 [107]	Wrong study design
EssentiaHealth 2010 [108]	Wrong patient population
Fan 2016 [109]	Wrong patient population
Ferdowsian 2010 [110]	Wrong patient population
Fleming 2019 [111]	Wrong patient population
Flynn 2009 [112]	Wrong patient population
Gardner 2005 [113]	Wrong patient population
Garousi 2023 [114]	Wrong patient population
Garousi 2021 [115]	Wrong patient population
Gonciulea 2015 [116]	Wrong patient population
Gonciulea 2017 [117]	Wrong patient population

Guagnano 2021 [118]	Wrong intervention
Gupta 2023 [119]	Wrong study design
Haag 2023 [120]	Wrong intervention
Hakala 1989 [121]	Wrong intervention
Hartmann 2023 [122]	Wrong intervention
Hartmann 2022 [123]	Wrong intervention
Haub 2005 [124]	Wrong intervention
Hentges 1978 [125]	Wrong study design
Hrachovinová 2009 [126]	Wrong patient population
Huber 2023 [127]	Wrong patient population
Huber 2021 [128]	Wrong patient population
Huber 2023 [129]	Wrong patient population
Huber 2021 [130]	Wrong patient population
Huber 2021 [131]	Wrong patient population
Hunt 1998 [132]	Wrong patient population
Hyder 2009 [133]	Wrong patient population
ImperialCollegeLondon 2012 [134]	Wrong intervention
IRCT20140208016529N2 2018 [135]	Wrong patient population
IRCT20150909023957N10 2022 [136]	Wrong patient population
Irct20190427043387N 2019 [137]	Wrong intervention
IRCT20190717044244N1 2019 [138]	Wrong intervention
IRCT20230821059213N1 2023 [139]	Wrong intervention
ISRCTN10560738 2021 [140]	Wrong intervention
ISRCTN53814211 2023 [141]	Wrong intervention

ISRCTN69541705 2020 [142]	Wrong patient population
Jenkins 2022 [143]	Wrong patient population
Jenkins 2008 [144]	Wrong study design
Johansson 1994 [145]	Wrong patient population
Johnstone 2010 [146]	Wrong intervention
Jprn 2022 [147]	Wrong intervention
JPRN-UMIN000019061 2016 [148]	Wrong study design
JPRN-UMIN000032071 2018 [149]	Wrong intervention
JPRN-UMIN000038293 2019 [150]	Wrong patient population
JPRN-UMIN000048081 2022 [151]	Wrong intervention
Kafyra 2021 [152]	Wrong study design
Kahleova 2023 [153]	Wrong patient population
Kahleova 2022 [154]	Wrong study design
Kahleova 2014 [155]	Wrong patient population
Kahleova 2017 [156]	Wrong patient population
Kahleova 2020 [157]	Intervention duration <7 days
Kahleova 2013 [158]	Wrong patient population
Kahleova 2011 [159]	Wrong patient population
Kahleova 2011 [160]	Wrong patient population
Kahleova 2011 [161]	Wrong patient population
Kahleova 2010 [162]	Wrong patient population
Kahleova 2009 [163]	Wrong patient population
Kahleova 2023 [164]	Wrong outcomes
Kahleova 2021 [165]	Intervention duration <7 days

Kahleova 2016 [166]	Wrong patient population
Kahleova 2018 [167]	Duplicate
Kahleova 2019 [168]	Wrong intervention
Kahleova 2023 [169]	Wrong patient population
Karlsson 1994 [170]	Wrong patient population
KCT0001771 2016 [171]	Wrong patient population
Kestin 1989 [172]	Wrong patient population
Kies 1988 [173]	Wrong study design
Kim 2013 [174]	Wrong study design
Kim 2012 [175]	Wrong patient population
Klementova 2019 [176]	Intervention duration <7 days
Koebnick 2004 [177]	Wrong intervention
Krenek 2023 [178]	Wrong patient population
Kudláčková 2020 [179]	Wrong intervention
Landry 2023 [180]	Wrong intervention
Lederer 2019 [181]	Wrong patient population
Lee 2018 [182]	Wrong patient population
Lee 2017 [183]	Wrong patient population
Lee 2016 [184]	Wrong patient population
Leslie 2002 [185]	Wrong intervention
Link 2023 [186]	Wrong study design
Lisevick 2021 [187]	Wrong intervention
Lowe 2020 [188]	Wrong intervention
Lukaszuk 2002 [189]	Wrong patient population

MaastrichtUniversityMedicalCenter 2021 [190]	Intervention duration <7 days
Malek 2021 [191]	Wrong study design
Margetts 1987 [192]	Wrong patient population
Marniemi 1990 [193]	Wrong intervention
Martineta 2019 [194]	Wrong intervention
Masarei 1984 [195]	Wrong patient population
McDougall 2002 [196]	Wrong study design
McGillUniversityHealthCentre/ResearchInstituteoftheMcGillUniversityHealthCentre 2020 [197]	Intervention duration <7 days
Memorial Sloan Kettering Cancer Center 2021 [198]	Wrong patient population
Memorial Sloan Kettering Cancer Center 2020 [199]	Wrong patient population
Mia 2000 [200]	Wrong patient population
MirelaZec 2011 [201]	Wrong patient population
Mishra 2013 [202]	Wrong patient population
Mishra 2012 [203]	Wrong patient population
Mishra 2013 [204]	Wrong patient population
Mocanu 2022 [205]	Wrong patient population
Mohammad 2019 [206]	Wrong patient population
Moore 2015 [207]	Wrong intervention
Morgan-Bathke 2019 [208]	Wrong study design
Murphy 2020 [209]	Wrong patient population
Muti 2003 [210]	Wrong patient population
Najafabadi 2023 [211]	Wrong study design
Nct 2016 [212]	Duplicate

Nct 2017 [213]	Wrong patient population
Nct 2012 [214]	Wrong intervention
Nct 2015 [215]	Wrong intervention
Nct 2018 [216]	Wrong patient population
Nct 2018 [217]	Duplicate
Nct 2023 [218]	Wrong intervention
Nct 2022 [219]	Wrong intervention
Nct 2012 [220]	Wrong patient population
Nct 2021 [221]	Intervention duration <7 days
Nct 2020 [222]	Wrong intervention
Nct 2023 [223]	Intervention duration <7 days
Nct 2022 [224]	Wrong intervention
Nct 2018 [225]	Wrong patient population
Nct 2016 [226]	Wrong patient population
NCT00276939 2006 [227]	Wrong patient population
NCT00330629 2006 [228]	Wrong intervention
NCT00883038 2008 [229]	Wrong patient population
NCT01222429 2010 [230]	Wrong patient population
NCT01412346 2011 [231]	Wrong patient population
NCT01544101 2012 [232]	Wrong patient population
NCT01547494 2012 [233]	Wrong patient population
NCT01593423 2012 [234]	Wrong patient population
NCT01690962 2012 [235]	Wrong patient population
NCT01699009 2012 [236]	Wrong patient population

NCT01700868 2012 [237]	Wrong patient population
NCT01716429 2012 [238]	Wrong patient population
NCT01817491 2013 [239]	Wrong patient population
NCT01931631 2013 [240]	Wrong patient population
NCT01953757 2013 [241]	Wrong patient population
NCT02080325 2014 [242]	Wrong intervention
NCT02135939 2014 [243]	Wrong patient population
NCT02153138 2014 [244]	Wrong study design
NCT02562209 2015 [245]	Wrong intervention
NCT02651480 2016 [246]	Wrong patient population
NCT02816814 2016 [247]	Wrong intervention
NCT02942628 2016 [248]	Wrong patient population
NCT03045289 2017 [249]	Wrong patient population
NCT03134235 2017 [250]	Wrong study design
NCT03315988 2017 [251]	Wrong study design
NCT03416777 2018 [252]	Wrong patient population
NCT03608176 2018 [253]	Wrong intervention
NCT03798938 2019 [254]	Wrong study design
NCT03901183 2019 [255]	Wrong intervention
NCT03925142 2019 [256]	Wrong intervention
NCT03962335 2019 [257]	Wrong patient population
NCT04018040 2019 [258]	Wrong patient population
NCT04048642 2019 [259]	Wrong study design
NCT04091516 2019 [260]	Wrong study design

NCT04105608 2019 [261]	Wrong patient population
NCT04171778 2019 [262]	Wrong patient population
NCT04297852 2019 [263]	Wrong patient population
NCT04316429 2020 [264]	Wrong patient population
NCT04347213 2020 [265]	Wrong study design
NCT04349059 2020 [266]	Wrong patient population
NCT04801745 2021 [267]	Wrong patient population
NCT04820829 2021 [268]	Wrong intervention
NCT04923022 2021 [269]	Wrong patient population
NCT05071196 2021 [270]	Wrong intervention
NCT05351853 2021 [271]	Wrong study design
NCT05359848 2022 [272]	Wrong study design
NCT05410002 2022 [273]	Wrong intervention
NCT05624333 2022 [274]	Wrong patient population
NCT05814874 2023 [275]	Wrong intervention
NCT05860088 2023 [276]	Wrong patient population
NCT05885828 2023 [277]	Wrong intervention
NCT05985369 2023 [278]	Wrong intervention
NCT06172725 2023 [279]	Wrong intervention
Neuenschwander 2019 [280]	Wrong study design
Neumann 2013 [281]	Wrong patient population
Nicholson 1999 [282]	Wrong patient population
Njike 2023 [283]	Wrong patient population
Nl 2019 [284]	Duplicate

Nl 2019 [285]	Duplicate
NL7800 2019 [286]	Wrong patient population
NL7801 2019 [287]	Duplicate
OchsnerHealthSystem 2015 [288]	Wrong study design
Ohio State University Comprehensive Cancer Center 2017 [289]	Wrong intervention
OhioUniversity 2011 [290]	Wrong intervention
Olsen 2023 [291]	Wrong intervention
OregonResearchInstitute 1993 [292]	Wrong study design
Ornish 1990 [293]	Wrong patient population
Ornish 2001 [294]	Wrong patient population
Pagliai 2016 [295]	Wrong patient population
Pagliai 2019 [296]	Duplicate
Paivarinta 2020 [297]	Wrong intervention
Park 2019 [298]	Wrong patient population
Pasanisi 2017 [299]	Wrong patient population
Pham 2022 [300]	Wrong intervention
PhoenixVAHealthCareSystem 2015 [301]	Wrong intervention
PhysiciansCommitteeforResponsibleMedicine 2020 [302]	Wrong patient population
PhysiciansCommitteeforResponsibleMedicine 2019 [303]	Wrong study design
PhysiciansCommitteeforResponsibleMedicine 2014 [304]	Wrong patient population
PhysiciansCommitteeforResponsibleMedicine 2003 [305]	Wrong patient population
PhysiciansCommitteeforResponsibleMedicine 2021 [306]	Wrong patient population
Piacquadio 2023 [307]	Wrong patient population
Pierce 2002 [308]	Wrong patient population

Pirke 1986 [309]	Wrong patient population
Poikunnel Chacko [310]	Wrong study design
Purdue University 2013, NCT01006343 [311]	Wrong intervention
Rauma 1993 [312]	Wrong patient population
Richter 1991 [313]	Wrong patient population
Rinott 2022 [314]	Wrong intervention
Rock 1997 [315]	Wrong patient population
Rogerson 2018 [316]	Wrong patient population
Sabino 2017 [317]	Wrong study design
Sacks 2002 [318]	Wrong study design
Sacks 1984 [319]	Wrong patient population
Sandkühler 2023 [320]	Wrong intervention
Sathiaraj 2023 [321]	Wrong intervention
Scherwitz 1995 [322]	Wrong study design
Schieren 2023 [323]	Wrong intervention
Sciarrillo 2023 [324]	Wrong patient population
Sela 2020 [325]	Wrong intervention
Shah 2017 [326]	Wrong study design
Shah 2022 [327]	Wrong intervention
Shin 2020 [328]	Wrong study design
Skoldstam 2005 [329]	Wrong study design
Slavícek 2001 [330]	Wrong patient population
Slavíček 2008 [331]	Wrong patient population
Slavíček 2007 [332]	Wrong patient population

Soroka 1998 [333]	Wrong patient population
Spiller 2003 [334]	Wrong patient population
St Michael's Hospital, Toronto 2014 [335]	Wrong intervention
StanfordUniversity 2019 [336]	Wrong patient population
Swapnali 2016 [337]	Wrong patient population
Tang 2021 [338]	Wrong intervention
TCTR20220119008 2022 [339]	Wrong study design
Tel-AvivSouraskyMedicalCenter 2018 [340]	Wrong patient population
Tel-AvivSouraskyMedicalCenter 2021 [341]	Wrong study design
TheClevelandClinic 2016 [342]	Duplicate
TheClevelandClinic 2013 [343]	Duplicate
Thomas 2022 [344]	Wrong intervention
Thomson 2005 [345]	Wrong patient population
Tsaban 2021 [346]	Wrong intervention
Turner-McGrievy 2022 [347]	Wrong intervention
Turner-McGrievy 2008 [348]	Wrong patient population
Turner-McGrievy 2014 [349]	Wrong study design
Turner-McGrievy 2014 [350]	Wrong patient population
Turner-McGrievy 2015 [351]	Wrong intervention
Turner-McGrievy 2016 [352]	Wrong outcomes
Turner-McGrievy 2023 [353]	Wrong intervention
Turner-McGrievy 2023 [354]	Wrong intervention
Turner-McGrievy 2015 [355]	Wrong intervention
Turner-McGrievy 2020 [356]	Wrong intervention

UCSF Benioff Children's Hospital Oakland 2016 [357]	Wrong intervention
UniversitätDuisburg-Essen 2014 [358]	Wrong patient population
UniversityofAlabamaatBirmingham 2006 [359]	Wrong patient population
UniversityofCampinas 2020 [360]	Wrong intervention
UniversityofRochester 2020 [361]	Wrong study design
UniversityofSouthCarolina 2021 [362]	Wrong patient population
UniversityofSouthCarolina 2018 [363]	Wrong patient population
UniversityofToronto 2003 [364]	Wrong study design
UniversityofToronto 2007 [365]	Wrong intervention
Varaeva 2018 [366]	Wrong patient population
Veleba 2019 [367]	Intervention duration <7 days
Veleba 2016 [368]	Wrong patient population
vonLossonczy 1978 [369]	Wrong patient population
Walrabenstein 2021 [370]	Wrong intervention
Walrabenstein 2023 [371]	Wrong intervention
Walrabenstein 2023 [372]	Wrong intervention
Walrabenstein 2022 [373]	Wrong intervention
Wang 2023 [374]	Wrong intervention
Warziski 2008 [375]	Wrong intervention
Washington University School of Medicine 2016 [376]	Wrong study design
Watts 1988 [377]	Wrong intervention
Wright 2017 [378]	Wrong patient population
Yadav 2014 [379]	Wrong patient population
Yadav 2016 [380]	Wrong patient population

Yamashita 1998 [381]	Wrong study design
YaskolkaMeir 2021 [382]	Wrong intervention
Zelicha 2022 [383]	Wrong intervention
Zhu 2021 [384]	Wrong patient population
Whole-Food Plant-Based Diet to Control Weight and MetaboInflammation in Overweight/Obese Men With Prostate Cancer, NCT05471414 [385]	Wrong intervention
Whole Food Plant-Based Diet for HIV-Associated Reduction in Cardiovascular Risk (PLANT-HART) [386]	Wrong patient population
Weight Management for the Remission of Type 2 Diabetes Using a Proprietary Meal Replacement System- Diabetes Remission Study (DRS) NCT05397028 [387]	Wrong intervention
Weight Loss Intervention With Lean Muscle Mass Retention NCT05607628 [388]	Wrong intervention
Twins Nutrition Study (TwiNS): Vegan vs. Omnivore NCT05297825 [389]	Wrong intervention
The Impact of Plant-Based Protein-rich Food Products With Varying Degree of Processing on the Human Gut Microbiome Composition and Human Metabolome [390]	Wrong patient population
The Effect of Vegetarian Diet on Patients With Metabolic Associated Fatty Liver Disease NCT05443581 [391]	Wrong patient population
The Effect of an Online Plant-Based Dietary Program on Cardiovascular Risk Factors in Persons With Type 2 Diabetes Mellitus: A Randomized Controlled Trial NCT05777746 [392]	Wrong intervention
The Effect of a 2-week Preoperative Vegan Diet Versus Omnivorous Diet on the Protein Turnover in the Osteoarthritic Knee [393]	Wrong patient population

The Effect of a 12-week Self-composed Vegan Diet With or Without Concurrent Resistance Exercise on Thigh Muscle Volume in Older Adults [394]	Wrong patient population
The Dietary Guidelines 3 Diet Patterns Study (DG3D): Phase 2 [395]	Wrong intervention
The CARING Study: Creating and Restoring Health Through Nutrition Guidance NCT05795439 [396]	Wrong patient population
Study of Nutrition in Postpartum and Early Life Feeding Study NCT06082921 [397]	Intervention duration <7 days
Singapore Healthy Alternative Protein Evaluation Study [398]	Wrong patient population
Role of Lean-pork Within a Plant-based Dietary Pattern [399]	Wrong patient population
Remotely Delivered Behavioral Weight Loss Using an Ad Libitum Plant-Based Diet Versus a Balanced Calorie Deficit Diet NCT05337150 [400]	Wrong intervention
Protein Quantity and Quality in Older Subjects NCT05301179 [401]	Wrong intervention
Plant-Focused Nutrition in Patients With Diabetes and Chronic Kidney Disease NCT05514184 [402]	Wrong intervention
Nutritious Eating With Soul Dissemination and Implementation [403]	Wrong intervention
Nutritional Intervention for Endometriosis [404]	Wrong patient population
Multimodal Project NCT05656716 [405]	Wrong study design
Low-Carbohydrate and Plant-Based Dietary Effects on Vascular Health [406]	Wrong patient population
Implementation of a Mediterranean Diet Program for Overweight or Obese Pregnant Women in a Low-resource Clinical Setting NCT05868954 [407]	Wrong intervention
Impact of a Mediterranean Diet on Cardiovascular Disease Risk Factors NCT06113484 [408]	Wrong intervention

Growth Study Using Else Toddler Nutritional Drink vs. a. Dairy Based Toddler Drink (Control) in Healthy Toddlers NCT05576870 [409]	Wrong intervention
Fasting-mimicking Diet in Treatment of Depressive Symptoms in IBD NCT05382897 [410]	Intervention duration <7 days
Fasting-mimicking Diet and Longevity Diet, Body Composition and Aging NCT05698654 [411]	Intervention duration <7 days
Effects of Pulses Through the Gut Microbiome and Bioavailability of Bioactive Compounds [412]	Wrong patient population
Effects of Lean Pork Loin Intake on Protein Homeostasis and Glucose Regulation in Prediabetic Adults NCT06025292 [413]	Intervention duration <7 days
Effects of Diet-Modulated Autologous Fecal Microbiota Transplantation on Weight Regain 2020 [414]	Wrong intervention
Effects of Beef Consumption on Skeletal Muscle Protein Homeostasis and Inflammatory Factors in Pre- and Postmenopausal Females NCT05714462 [415]	Intervention duration <7 days
Effect of Tirzepatide Plus Intensive Lifestyle Therapy on Body Weight and Metabolic Health in Latinos With Obesity NCT06009653 [416]	Wrong intervention
Effect of the Sustainable Diet on Gut Microbiota and the Metabolome: a Randomised Crossover Study [417]	Wrong patient population
Effect of Partial Dietary Replacement From Animal to Plant-Based Protein for Type 2 Diabetes Management NCT05706155 [418]	Wrong intervention
Effect of Low-Calorie Diet and Lifestyle Intervention on Reversal of T2DM NCT05925946 [419]	Wrong intervention
Effect of Intermittent Calorie Restriction on MASLD Patients With Abnormal Glucose Metabolism NCT04283942 [420]	Wrong intervention

Effect of a Vegetarian Meal on the Physiology of Insulin Response in Patients With Type 2 Diabetes Mellitus and Obesity NCT06152536 [421]	Wrong intervention
Effect of a Pulse-based USDA-diet on Healthspan [422]	Wrong patient population
Digestibility of Different Plant-based Proteins in Humans With Ileostomy NCT06142084 [423]	Intervention duration <7 days
Diets, Lipoproteins and Inflammation Markers [424]	Wrong patient population
Dietary Proteins: Metagenomic and Metabolomics Approaches for Human Biomarkers Identification NCT05611138 [425]	Wrong intervention
Dietary Intervention to Improve Kidney Transplant Outcomes NCT05449496 [426]	Wrong intervention
Behavioral Plant-Based Dietary Intervention in Latinos NCT05444595 [427]	Wrong intervention
Athlete Whey Protein Sensitivity: Prevalence and Performance NCT05482997 [428]	Wrong intervention
A Study of a Plant-Based Diet and Dietary Supplements in People With Smoldering Multiple Myeloma (SMM) NCT06055894 [429]	Wrong intervention

References

1. Acharya, S.D.; Brooks, M.M.; Evans, R.W.; Linkov, F.; Burke, L.E. Weight loss is more important than the diet type in improving adiponectin levels among overweight/obese adults. *Journal of the American College of Nutrition* **2013**, *32*, 264-271, doi:https://dx.doi.org/10.1080/07315724.2013.816607.
2. Acharya, S.D.; Elci, O.U.; Sereika, S.M.; Music, E.; Styn, M.A.; Turk, M.W.; Burke, L.E. Adherence to a behavioral weight loss treatment program enhances weight loss and improvements in biomarkers. *Patient preference & adherence* **2009**, *3*, 151-160.
3. Actrn. Effectiveness of exercises in non-weight positions combined with mobile health on pain, body mass index and exercise adherence in overweight and obese knee osteoarthritis patients: a randomized controlled trial. https://trialsearch.who.int/Trial2.aspx?TrialID=ACTRN12621001628831 **2021**.
4. Actrn. Do moderate protein, high carbohydrate predominantly plant-based diets assist in appetite control and improve short-term health outcomes in individuals aged 65-75 years? http://www.who.int/trialsearch/Trial2.aspx?TrialID=ACTRN12616001606471 **2016**.
5. Actrn. A dietary study investigating the beneficial effects of a traditional Mediterranean diet on weight, diabetes control and heart disease risk in people with Type2diabetes. http://www.who.int/trialsearch/Trial2.aspx?TrialID=ACTRN12607000394448 **2007**.
6. Actrn; National, H.; Medical Research, C. A dietary study investigating the beneficial effects of a traditional Mediterranean diet on weight, diabetes control and heart disease risk in people with Type 2 diabetes. **2007**. http://www.who.int/trialsearch/Trial2.aspx?TrialID=ACTRN12607000394448

7. Actrn; Monash University, Y.; St. Vincent's Hospital Melbourne Not, a. Lifestyle Intervention for early diabetes: A randomised controlled trial. **2011**. https://www.anzctr.org.au/Trial/Registration/TrialReview.aspx?id=336676&isReview=true
8. Actrn; Dr Nicholas, W. The BROAD study. A trial using the whole-foods, plant-based diet in a community programme for people with obesity, or overweight with ischaemic heart disease or diabetes. **2014**. https://www.anzctr.org.au/Trial/Registration/TrialReview.aspx?ACTRN=12614000395639
9. Actrn; Dr Nicholas, W. The EDGe (End Diabetes Gisborne) trial. Using the whole-foods, plant-based diet in a community programme for people with obesity and diabetes. **2017**. https://anzctr.org.au/Trial/Registration/TrialReview.aspx?ACTRN=12617000541303
10. Actrn; The University of Sydney, Y. Effects of animal and plant origin diet on sleep health in healthy adults. **2018**. https://anzctr.org.au/Trial/Registration/TrialReview.aspx?id=375760&isReview=true
11. Actrn; The University of, S. A randomised controlled trial to evaluate an intensive lifestyle program for reversal of coronary heart disease. **2020**. https://www.anzctr.org.au/Trial/Registration/TrialReview.aspx?ACTRN=12620001151921
12. Actrn; University of Newcastle, Y. Investigating the Effect of Plant Based Diets on Cardiovascular Disease Risk. **2021**. https://www.anzctr.org.au/Trial/Registration/TrialReview.aspx?id=381515&isReview=true
13. Actrn; Exercise Medicine Research Institute. Edith Cowan University, Y. The FASTEX trial: Fasting mimicking diet and exercise medicine as adjuvant therapies in the treatment of patients with breast cancer. **2023**. https://anzctr.org.au/Trial/Registration/TrialReview.aspx?ACTRN=12623000996662
14. Aldubayan, M.A.; Mao, X.; Laursen, M.F.; Pigsborg, K.; Christensen, L.H.; Roager, H.M.; Nielsen, D.S.; Hjorth, M.F.; Magkos, F. Supplementation with inulin-type fructans affects gut microbiota and attenuates some of the cardiometabolic benefits of a plant-based diet in individuals with overweight or obesity. *Frontiers in Nutrition* **2023**, *10*, 1108088, doi:https://dx.doi.org/10.3389/fnut.2023.1108088.
15. Arntzenius, A.C. Diet, lipoproteins and the progression of coronary atherosclerosis. The Leiden Intervention Trial. *Drugs* **1986**, *31 Suppl 1*, 61-65.
16. Arora, R.C.; Agarwal, N.; Arora, S.; Gupta, G.C. A study of high fat and cholesterol diet induced changes in plasma cholesterol and lipoprotein in healthy human volunteers--vegetarians. *Materia medica Polona. Polish journal of medicine and pharmacy* **1986**, *18*, 198-202.
17. Baden, M.Y.; Satija, A.; Hu, F.B.; Huang, T. Change in Plant-Based Diet Quality Is Associated with Changes in Plasma Adiposity-Associated Biomarker Concentrations in Women. *Journal of Nutrition* **2019**, *149*, 676-686, doi:https://dx.doi.org/10.1093/jn/nxy301.
18. Ball, D.; Maughan, R.J. Blood and urine acid-base status of premenopausal omnivorous and vegetarian women. *British Journal of Nutrition* **1997**, *78*, 683-693, doi:10.1079/BJN19970187.
19. Barbara Jakše, s.p. Low Fat Plant-based Diet Effects on Body Composition Indices. **2011**. https://clinicaltrials.gov/study/NCT02906072
20. Barnard, N.; Cohen, J.; Jenkins, D.J.; Turner-McGrievy, G.; Ferdowsian, H. Randomized clinical trial of a plant-based diet for glycemic, lipid, and weight control in type 2 diabetes: follow-up results. *Diabetes* **2007**, *56*, A448-A.
21. Barnard, N.D.; Alwarith, J.; Rembert, E.; Brandon, L.; Nguyen, M.; Goergen, A.; Horne, T.; do Nascimento, G.F.; Lakkadi, K.; Tura, A.; et al. A Mediterranean Diet and Low-Fat Vegan Diet to Improve Body Weight and Cardiometabolic Risk Factors: A Randomized, Cross-over Trial. *Journal of the American College of Nutrition* **2020**, doi:10.1080/07315724.2020.1869625.
22. Barnard, N.D.; Bunner, A.E.; Wells, C.L.; Gonzales, J.; Agarwal, U.; Bayat, E.; Xu, J. A dietary intervention for chronic diabetic neuropathy pain: A randomized controlled pilot study. *Diabetes/Metabolism Research and Reviews* **2015**, *31*, 33, doi:10.1002/dmrr.2727.
23. Barnard, N.D.; Cohen, J.; Jenkins, D.J.; Turner-McGrievy, G.; Gloede, L.; Green, A.; Ferdowsian, H. A low-fat vegan diet and a conventional diabetes diet in the treatment of type 2 diabetes: a randomized, controlled, 74-wk clinical trial. *American Journal of Clinical Nutrition* **2009**, *89*, 1588S-1596S, doi:https://dx.doi.org/10.3945/ajcn.2009.26736H.
24. Barnard, N.D.; Cohen, J.; Jenkins, D.J.; Turner-McGrievy, G.; Gloede, L.; Jaster, B.; Seidl, K.; Green, A.A.; Talpers, S. A low-fat vegan diet improves glycemic control and cardiovascular risk factors in a randomized clinical trial in individuals with type 2 diabetes. *Diabetes Care* **2006**, *29*, 1777-1783.
25. Barnard, N.D.; Gloede, L.; Cohen, J.; Jenkins, D.J.; Turner-McGrievy, G.; Green, A.A.; Ferdowsian, H. A low-fat vegan diet elicits greater macronutrient changes, but is comparable in adherence and acceptability, compared with a more conventional diabetes diet among individuals with type 2 diabetes. *Journal of the American Dietetic Association* **2009**, *109*, 263-272, doi:https://dx.doi.org/10.1016/j.jada.2008.10.049.

26. Barnard, N.D.; Kahleova, H.; Holtz, D.N.; Del Aguila, F.; Neola, M.; Crosby, L.M.; Holubkov, R. The Women's Study for the Alleviation of Vasomotor Symptoms (WAVS): a randomized, controlled trial of a plant-based diet and whole soybeans for postmenopausal women. *Menopause* **2021**, *28*, 1150-1156, doi:https://dx.doi.org/10.1097/GME.0000000000001812.
27. Barnard, N.D.; Kahleova, H.; Holtz, D.N.; Znayenko-Miller, T.; Sutton, M.; Holubkov, R.; Zhao, X.; Galandi, S.; Setchell, K.D.R. A dietary intervention for vasomotor symptoms of menopause: a randomized, controlled trial. *Menopause* **2023**, *30*, 80-87, doi:https://dx.doi.org/10.1097/GME.0000000000002080.
28. Barnard, N.D.; Levin, S.M.; Gloede, L.; Flores, R. Turning the Waiting Room into a Classroom: Weekly Classes Using a Vegan or a Portion-Controlled Eating Plan Improve Diabetes Control in a Randomized Translational Study. *Journal of the Academy of Nutrition & Dietetics* **2018**, *118*, 1072-1079, doi:https://dx.doi.org/10.1016/j.jand.2017.11.017.
29. Barnard, N.D.; Noble, E.P.; Ritchie, T.; Cohen, J.; Jenkins, D.J.; Turner-McGrievy, G.; Gloede, L.; Green, A.A.; Ferdowsian, H. D2 dopamine receptor Taq1A polymorphism, body weight, and dietary intake in type 2 diabetes. *Nutrition* **2009**, *25*, 58-65, doi:https://dx.doi.org/10.1016/j.nut.2008.07.012.
30. Barnard, N.D.; Scialli, A.R.; Bertron, P.; Hurlock, D.; Edmonds, K.; Talev, L. Effectiveness of a low-fat vegetarian diet in altering serum lipids in healthy premenopausal women. *American Journal of Cardiology* **2000**, *85*, 969-972.
31. Barnard, N.D.; Scialli, A.R.; Hurlock, D.; Bertron, P. Diet and sex-hormone binding globulin, dysmenorrhea, and premenstrual symptoms. *Obstetrics & Gynecology* **2000**, *95*, 245-250.
32. Barrett, H.L.; Gomez-Arango, L.F.; Wilkinson, S.A.; McIntyre, H.D.; Callaway, L.K.; Morrison, M.; Dekker Nitert, M. A Vegetarian Diet Is a Major Determinant of Gut Microbiota Composition in Early Pregnancy. *Nutrients* **2018**, *10*, 12, doi:https://dx.doi.org/10.3390/nu10070890.
33. Beilin, L.J. Vegetarian and other complex diets, fats, fiber, and hypertension. *American Journal of Clinical Nutrition* **1994**, *59*, 1130S-1135S, doi:10.1093/ajcn/59.5.1130S.
34. Beilin, L.J.; Armstrong, B.K.; Margetts, B.M.; Rouse, I.L.; Vandongen, R. Vegetarian diet and blood pressure. *Nephron* **1987**, *47 Suppl 1*, 37-41.
35. Beilin, L.J.; Burke, V. Vegetarian diet components, protein and blood pressure: which nutrients are important? *Clinical & Experimental Pharmacology & Physiology* **1995**, *22*, 195-198.
36. Beilin, L.J.; Rouse, I.L.; Armstrong, B.K.; Margetts, B.M.; Vandongen, R. Vegetarian diet and blood pressure levels: incidental or causal association? *American Journal of Clinical Nutrition* **1988**, *48*, 806-810, doi:https://dx.doi.org/10.1093/ajcn/48.3.806.
37. Belinova, L.; Kahleova, H.; Malinska, H.; Topolcan, O.; Vrzalova, J.; Oliyarnyk, O.; Kazdova, L.; Hill, M.; Pelikanova, T. Differential acute postprandial effects of processed meat and isocaloric vegan meals on the gastrointestinal hormone response in subjects suffering from type 2 diabetes and healthy controls: a randomized crossover study. *PLoS ONE [Electronic Resource]* **2014**, *9*, e107561, doi:https://dx.doi.org/10.1371/journal.pone.0107561.
38. Bendinelli, B.; Masala, G.; Bella, C.D.; Assedi, M.; Benagiano, M.; Pratesi, S.; Ermini, I.; Occhini, D.; Castaldo, M.; Saieva, C.; et al. Adipocytokine plasma level changes in a 24-month dietary and physical activity randomised intervention trial in postmenopausal women. *European Journal of Nutrition* **2023**, *62*, 1185-1194, doi:10.1007/s00394-022-03055-y.
39. Benson, S. Comparing diets for weight loss and improvement in biomarkers in men with prostate cancer on surveillance: a pilot study. *Journal of clinical oncology* **2017**, *35*, 159, doi:10.1200/JCO.2017.35.5_suppl.159
40. Bergeron, N.; Chiu, S.; Williams, P.T.; M King, S.; Krauss, R.M. Effects of red meat, white meat, and nonmeat protein sources on atherogenic lipoprotein measures in the context of low compared with high saturated fat intake: a randomized controlled trial. *American Journal of Clinical Nutrition* **2019**, *110*, 24-33, doi:10.1093/ajcn/nqz035.
41. Bernhart, J.A.; Turner-McGrievy, G.M.; Wilson, M.J.; Sentman, C.; Wilcox, S.; Rudisill, C. NEW Soul in the neighborhood-reach and effectiveness of a dissemination and implementation feasibility study. *Translational Behavioral Medicine* **2023**, *13*, 123-131, doi:https://dx.doi.org/10.1093/tbm/ibac080.
42. Beth Israel Deaconess Medical, C. Effect of a Plant Rich Diet on in People With Type 2 Diabetes and Obesity. **2019**. https://ClinicalTrials.gov/show/NCT02977039
43. Bhardwaj, S.; Misra, A.; Gulati, S.; Anoop, S.; Kamal, V.K.; Pandey, R.M. A randomized controlled trial to evaluate the effects of high Protein Complete (lActo) VEgetaRian (PACER) diet in non-diabetic obese Asian Indians in North India. *Heliyon* **2017**, *3*, e00472, doi:https://dx.doi.org/10.1016/j.heliyon.2017.e00472.
44. Bo, F.-R.; Rigshospitalet, D. Long-term Effects of the New Nordic Renal Diet in Patients With Moderate Chronic Kidney Disease. **2020**. https://ClinicalTrials.gov/show/NCT04579315

45. Bouzas, C.; Bibiloni, M.M.; Garcia, S.; Mateos, D.; Martínez-González, M.Á.; Salas-Salvadó, J.; Corella, D.; Schröder, H.; Martínez, J.A.; Alonso-Gómez, Á.M.; et al. Dietary quality changes according to the preceding maximum weight: A longitudinal analysis in the predimed-plus randomized trial. *Nutrients* **2020**, *12*, 1-15, doi:10.3390/nu12103023.
46. Braden, M.; Gwin, J.; Leidy, H. Effect of Protein Source From Lean Red Meat vs. Plant-Based Alternatives on Appetite Control, Satiety, and Energy Intake in Healthy Women With Overweight. *Current developments in nutrition* **2023**, *7*, doi:10.1016/j.cdnut.2023.100622.
47. Brestrich, M.; Claus, J.; Blümchen, G. Lactovegetarian diet: Influence on weight, lipids, fibrinogen and lipoprotein (a) of heart-patients in the course of their clinical rehabilitation. *Zeitschrift fur Kardiologie* **1996**, *85*, 418-427.
48. Brooke, O.G.; Brown, I.R.; Bone, C.D.; Carter, N.D.; Cleeve, H.J.; Maxwell, J.D.; Robinson, V.P.; Winder, S.M. Vitamin D supplements in pregnant Asian women: effects on calcium status and fetal growth. *British Medical Journal* **1980**, *280*, 751-754.
49. Bunner, A.E.; Agarwal, U.; Gonzales, J.F.; Valente, F.; Barnard, N.D. Nutrition intervention for migraine: a randomized crossover trial. *Journal of Headache & Pain* **2014**, *15*, 69, doi:https://dx.doi.org/10.1186/1129-2377-15-69.
50. Bunner, A.E.; Gonzalez, J.; Agarwal, U.; Valente, F.; Barnard, N.D. Nutrition intervention for diabetic neuropathy. *Diabetes* **2014**, *63*, A578, doi:10.2337/db14-2207-2292.
51. Bunner, A.E.; Wells, C.L.; Bayat, E.; Barnard, N.D. Nutrition intervention for diabetic neuropathy. *Endocrine Reviews* **2015**, *36*, Issue Supplement, i176, doi: https://doi.org/10.1093/edrv/36.supp.1
52. Bunner, A.E.; Wells, C.L.; Gonzales, J.; Agarwal, U.; Bayat, E.; Barnard, N.D. A dietary intervention for chronic diabetic neuropathy pain: a randomized controlled pilot study. *Nutrition & Diabetes* **2015**, *5*, e158, doi:https://dx.doi.org/10.1038/nutd.2015.8.
53. Bunner, A.; Gonzalez, J.; Valente, F.; Agarwal, U.; Barnard, N. A nutrition intervention for migraines. *Neurology* **2014**, *82*, 69, doi:https://doi.org/10.1186/1129-2377-15-69
54. Burke, L.E.; Choo, J.; Music, E.; Warziski, M.; Styn, M.A.; Kim, Y.; Sevick, M.A. PREFER study: A randomized clinical trial testing treatment preference and two dietary options in behavioral weight management - Rationale, design and baseline characteristics. *Contemporary Clinical Trials* **2006**, *27*, 34-48, doi:10.1016/j.cct.2005.08.002.
55. Burke, L.E.; Hudson, A.G.; Warziski, M.T.; Styn, M.A.; Music, E.; Elci, O.U.; Sereika, S.M. Effects of a vegetarian diet and treatment preference on biochemical and dietary variables in overweight and obese adults: a randomized clinical trial. *American Journal of Clinical Nutrition* **2007**, *86*, 588-596.
56. Burke, L.E.; Styn, M.A.; Steenkiste, A.R.; Music, E.; Warziski, M.; Choo, J. A randomized clinical trial testing treatment preference and two dietary options in behavioral weight management: Preliminary results of the impact of diet at 6 months - PREFER Study. *Obesity* **2006**, *14*, 2007-2017, doi:10.1038/oby.2006.235.
57. Burke, L.E.; Warziski, M.; Styn, M.A.; Music, E.; Hudson, A.G.; Sereika, S.M. A randomized clinical trial of a standard versus vegetarian diet for weight loss: the impact of treatment preference. *International Journal of Obesity* **2008**, *32*, 166-176.
58. Campbell, E.; Campbell, T.; Culakova, E.; Janelsins, M.; Shayne, M.; Mustian, K.; Lin, P.J.; Kamen, C.; Peppone, L. A whole-food, plant-based (WFPB) dietary intervention to improve cancer-related and cardiometabolic outcomes in metastatic breast cancer patients. *Supportive care in cancer* **2022**, *30*, S82-S83, doi:10.1007/s00520-022-07099-y.
59. Campbell, E.; Campbell, T.; Culakova, E.; Wixom, N.; Guido, J.; Blanchard, L.; Fetten, J.; Janelsins, M.; Mustian, K.; Peppone, L. A whole food, plant-based (WFPB) dietary intervention to improve cardiometabolic and cancer-related outcomes in women with breast cancer. *Cancer research* **2023**, *83*, doi:10.1158/1538-7445.SABCS22-P4-07-31.
60. Campbell, E.K.; Campbell, T.M.; Culakova, E.; Blanchard, L.M.; Wixom, N.; Guido, J.; Fetten, J.; Huston, A.; Shayne, M.; Janelsins, M.; et al. A Whole Food, Plant-Based Randomized Controlled Trial in Metastatic Breast Cancer: Feasibility, Nutrient, and Patient-Reported Outcomes. *Research Square* **2023**, *21*, 21, doi:https://dx.doi.org/10.21203/rs.3.rs-3606685/v1.
61. Campbell, T.; Campbell, E.; Culakova, E.; Janelsins, M.C.; Mustian, K.M.; Kamen, C.S.; Lin, P.J.; Guido, J.J.; Peppone, L.J. A whole-food, plant-based (WFPB) dietary intervention to improve cancer-related and cardiometabolic outcomes in metastatic breast cancer patients. *Journal of clinical oncology* **2022**, *40*, doi:10.1200/JCO.2022.40.16_suppl.e24116.
62. Campbell, T.; Campbell, E.; Culakova, E.; Wixom, N.; Guido, J.; Blanchard, L.; Janelsins, M.; Mustian, K.; Fetten, J.; Peppone, L. Feasibility of a Whole-Food, Plant-Based Intervention Among Women with

Metastatic Breast Cancer and its Effect on Patient-Reported Outcomes. *Cancer research* **2023**, *83*, doi:10.1158/1538-7445.SABCS22-P5-07-07.
63. Campbell, T.M.; Campbell, E.K.; Culakova, E.; Blanchard, L.; Wixom, N.; Guido, J.; Fetten, J.; Huston, A.; Shayne, M.; Janelsins, M.C.; et al. A Whole-Food, Plant-Based Randomized Controlled Trial in Metastatic Breast Cancer: weight, Cardiometabolic, and Hormonal Outcome. *Research Square* **2023**, doi:10.21203/rs.3.rs-3425125/v1.
64. Campbell, W.W.; Barton, M.L., Jr.; Cyr-Campbell, D.; Davey, S.L.; Beard, J.L.; Parise, G.; Evans, W.J. Effects of an omnivorous diet compared with a lactoovovegetarian diet on resistance-training-induced changes in body composition and skeletal muscle in older men. *American Journal of Clinical Nutrition* **1999**, *70*, 1032-1039.
65. ChiCtr; China-Japan Friendship, H. Effectiveness and safety of light vegetarian diet on functional constipation with gastrointestinal damp-heat pattern: an exploratory study. **2018**. https://www.chictr.org.cn/showproj.html?proj=31851
66. ChiCtr; China-Japan Friendship Hospital, Y. The diagnostic model for MAFLD based on non-invasive biomarkers in volatile organic compounds (VOCs) metabolomic profiling. **2023**. https://www.chictr.org.cn/hvshowproject.html?id=242513&v=1.0
67. Cleveland Clinic, F.; The Cleveland, C. Effects of Wolffia Globosa (Mankai) in Patients Undergoing Bariatric Surgery. **2019**. https://ClinicalTrials.gov/show/NCT03999632
68. Clinton, C.M.; O'Brien, S.; Law, J.; Renier, C.M.; Wendt, M.R. Whole-foods, plant-based diet alleviates the symptoms of osteoarthritis. *Arthritis* **2015**, *2015*, 708152, doi:10.1155/2015/708152.
69. Colombo, C.; Muti, P.; Pala, V.; Cavalleri, A.; Venturelli, E.; Locardi, M.; Berrino, F.; Secreto, G. Plant-based diet, serum fatty acid profile, and free radicals in postmenopausal women: the diet and androgens (DIANA) randomized trial. *International Journal of Biological Markers* **2005**, *20*, 169-176.
70. Cooper, R.S.; Goldberg, R.B.; Trevisan, M. The selective lipid-lowering effect of vegetarianism on low density lipoproteins in a cross-over experiment. *Atherosclerosis* **1982**, *44*, 293-305, doi:10.1016/0021-9150(82)90004-1.
71. Crimarco, A.; Dias, C.H.; Turner-McGrievy, G.M.; Wilson, M.; Adams, S.A.; Macauda, M.; Blake, C.E.; Younginer, N. Outcomes of a short term dietary intervention involving vegan soul food restaurants on African American adults' perceived barriers, benefits, and dietary acceptability of adopting a plant-based diet. *Food Quality and Preference* **2020**, *79*, 103788, doi:https://doi.org/10.1016/j.foodqual.2019.103788.
72. Ctri. Effect of Freedom from diabetes protocol on weight loss and HbA1c improvement in patient with Type 2 diabetes in India. https://trialsearch.who.int/Trial2.aspx?TrialID=CTRI/2023/06/053885 **2023**.
73. Ctri; Arun Daniel, J. The effect of different types of diet on the body weight and glycemic control of diabetics. **2018**. http://www.ctri.nic.in/Clinicaltrials/pmaindet2.php?trialid=20681
74. Ctri; Aiims, Y. To evaluate the effect and tolerance of a plant based diet in reducing the flare of clinical symptoms in patients with ulcerative colitis who are asymptomatic at time of study. **2022**. http://www.ctri.nic.in/Clinicaltrials/pmaindet2.php?trialid=50808
75. Ctri; All India Institute of Medical Sciences, Y. To evaluate the effect of a plant based diet in reducing the flare of clinical symptoms and its effect on the gut bacteria in patients with Crohns disease who are asymptomatic at time of study. **2022**. http://www.ctri.nic.in/Clinicaltrials/pmaindet2.php?trialid=50797
76. Ctri; Dr Spurti, R.P.Y. A comparative study between Vegetarian and Non-Vegetarian diets in male individuals. **2022**. http://www.ctri.nic.in/Clinicaltrials/pmaindet2.php?trialid=66073
77. Ctri; Krutuja Chaitravi Sudhir Hukeri, Y. Nutritional assessment of veganism v/s the non-vegetarian food habits in 20-60 year old individuals in India, in order to compare the disease prevalence in both the groups. **2022**. http://www.ctri.nic.in/Clinicaltrials/pmaindet2.php?trialid=67559
78. Ctri; Christian Medical college Vellore, Y. To study the role of 2 different diets in the management of type 2 diabetes. **2022**. http://www.ctri.nic.in/Clinicaltrials/pmaindet2.php?trialid=60473
79. Ctri; Freedom from diabetes research foundation, Y. Effect of Freedom from diabetes protocol on weight loss and HbA1c improvement in patient with Type 2 diabetes in India. **2023**. http://www.ctri.nic.in/Clinicaltrials/pmaindet2.php?trialid=55575
80. Ctri; Chellaram Diabetes Research Centre, Y. Effect Of 44-46% Carbohydrate (From Local And Traditional Sources) Diet with Regular Walk On Weight, Waist Circumference, Blood Pressure,Blood Glucose Levels, Blood lipid Levels. **2023**. http://www.ctri.nic.in/Clinicaltrials/pmaindet2.php?trialid=91258
81. Daniel-Gentry, J.; Dolecek, T.A.; Caggiula, A.W.; Van Horn, L.V.; Epley, L.; Randall, B.L. Increasing the use of meatless meals: a nutrition intervention substudy in the Multiple Risk Factor Intervention Trial (MRFIT). *Journal of the American Dietetic Association* **1986**, *86*, 778-781.

82. Daubenmier, J.J.; Weidner, G.; Marlin, R.; Crutchfield, L.; Dunn-Emke, S.; Chi, C.; Gao, B.; Carroll, P.; Ornish, D. Lifestyle and health-related quality of life of men with prostate cancer managed with active surveillance. *Urology* **2006**, *67*, 125-130.
83. David Grant, U.S.A.F.M.C. Facilitated Vegan Diet on Cardiometabolic Endpoints and Trimethylamine N-oxide. **2022**. https://clinicaltrials.gov/study/NCT05071196
84. Davis, B.C.; Jamshed, H.; Peterson, C.M.; Sabate, J.; Harris, R.D.; Koratkar, R.; Spence, J.W.; Kelly, J.H., Jr. An Intensive Lifestyle Intervention to Treat Type 2 Diabetes in the Republic of the Marshall Islands: Protocol for a Randomized Controlled Trial. *Frontiers in Nutrition* **2019**, *6*, 79, doi:https://dx.doi.org/10.3389/fnut.2019.00079.
85. de Vries-Postma, A.; Woldendorp, K.H.; Obenhuijsen, J. Nutrition & Lifestyle Intervention Program to Address Obesity and CVD Risks in Persons With SCI. *Archives of Physical Medicine and Rehabilitation* **2019**, *100*, e190-e191, doi:10.1016/j.apmr.2019.10.087.
86. Del Rocio Berglund, M.; Paxton, R.J.; Garcia-Prieto, C.; Hernandez, M.; Hajek, R.A.; Handy, B.C.; Brown, J.; Jones, L.A. Evaluation of a telephone-delivered dietary behavior intervention for ovarian cancer survivors. *FASEB Journal* **2012**, *26*, 626.6, doi:https://doi.org/10.1096/fasebj.26.1_supplement.626.6
87. Di Mauro, A.; Tuccinardi, D.; Watanabe, M.; Del Toro, R.; Monte, L.; Giorgino, R.; Rampa, L.; Rossini, G.; Kyanvash, S.; Soare, A.; et al. The Mediterranean diet increases glucagon-like peptide 1 and oxyntomodulin compared with a vegetarian diet in patients with type 2 diabetes: A randomized controlled cross-over trial. *Diabetes/Metabolism Research Reviews* **2021**, *37*, e3406, doi:https://dx.doi.org/10.1002/dmrr.3406.
88. Di Mauro, A.; Tuccinardi, D.; Watanabe, M.; Del Toro, R.; Monte, L.; Giorgino, R.; Rampa, L.; Rossini, G.; Kyanvash, S.; Soare, A.; et al. The Mediterranean diet increases glucagon-like peptide 1 and oxyntomodulin compared with a vegetarian diet in patients with type 2 diabetes: a randomized controlled cross-over trial. *Diabetes/Metabolism Research and Reviews* **2020**, doi:10.1002/dmrr.3406.
89. Dogra, S.; Getz, A.; Woolf, K.; Newman, J.D.; Xia, Y.; Slater, J.; Shah, B. Long-term Dietary and Weight Changes Following a Short-term Dietary Intervention Study: 4-year Follow-up of the Evade Cad Trial. *Circulation* **2020**, *142*, doi:10.1161/circ.142.suppl_3.15565.
90. Dressler, J.; Storz, M.A.; Muller, C.; Kandil, F.I.; Kessler, C.S.; Michalsen, A.; Jeitler, M. Does a Plant-Based Diet Stand Out for Its Favorable Composition for Heart Health? Dietary Intake Data from a Randomized Controlled Trial. *Nutrients* **2022**, *14*, 01, doi:https://dx.doi.org/10.3390/nu14214597.
91. Drexel, U. Evaluating a Remotely Delivered Plant-Based Behavioral Weight Loss Treatment. **2020**. https://clinicaltrials.gov/study/NCT04892030
92. Drks; Gesundheitspraxis, D. Does a whole-food, vegan diet provide the body with a sufficient supply of vitamin B12 and vitamin D? A follow-up study. **2013**. https://drks.de/search/en/trial/DRKS00004994
93. Drks; Klinik Buchinger Wilhelmi Gmb, H. Scientific documentation of the Buchinger Wilhelmi program (modified fasting, caloric restriction and vegetarian diet). **2016**. https://drks.de/search/en/trial/DRKS00010111
94. Drks; Fachhochschule des, M. Study on diet and health of vegetarian, vegan or omnivorous children and adolescents in Germany. **2018**. https://drks.de/search/en/trial/DRKS00012835
95. Drks; Psychotherapie und Systemneurowissenschaften, F.P.u.S.J.L.U.G. Unravelling the ´healthy´ in a healthy lifestyle: Dietary influences on subjective wellbeing and physical health. **2018**. https://drks.de/search/en/trial/DRKS00013702
96. Drks; Institut für Ernährungs- und Lebensmittelwissenschaften, A.E.R.F.-W.-U.B. Role of the immune system and the gut microbiota in neuroprotection: a dietary human intervention trial. **2019**. https://drks.de/search/en/trial/DRKS00015861
97. Drks; Klinik für Pädiatrie m. S. Onkologie / Hämatologie / Szt, A.G.I.M.Y. Planetary Health nutrition for students in the school canteen of a sports school in Berlin - Side project. **2023**. https://drks.de/search/en/trial/DRKS00030940
98. Drks; Universitätsklinikum Freiburg - Klinik für Innere Medizin, I.I.U.N.Y. Effects of a vegan diet compared to a meat-rich diet on the hemogram and inflammatory profile in healthy individuals: a randomized-controlled study. **2023**. https://drks.de/search/de/trial/DRKS00031541
99. Drks; Deutsche Sporthochschule Köln, N. The influence of a vegan diet on performance and the promotion of regeneration in competitive athletes. **2023**. https://drks.de/search/de/trial/DRKS00031633
100. Drks; Council for Agricultural, R.; Economics, Y. Food systems transformation towards healthy and sustainable dietary behaviour. **2023**. https://drks.de/search/de/trial/DRKS00031713

101. Eberhard, J.; Ruiz, K.; Tan, J.; Jayasinghe, T.; Khan, S.; Eroglu, E.; Adler, C.; Simpson, S.J.; Le Couteur, D.G.; Raubenheimer, D.; et al. A semi-vegetarian higher fat diet improves periodontal health - a randomised clinical trial. *Journal of Clinical Periodontology* **2022**, *49*, 143-144, doi:10.1111/jcpe.13636.
102. Eberhard, J.; Ruiz, K.; Tan, J.; Jayasinghe, T.N.; Khan, S.; Eroglu, E.; Adler, C.; Simpson, S.J.; Le Couteur, D.G.; Raubenheimer, D.; et al. A randomized clinical trial to investigate the effect of dietary protein sources on periodontal health. *Journal of Clinical Periodontology* **2022**, *49*, 388-400, doi:https://dx.doi.org/10.1111/jcpe.13587.
103. Eberhard, J.; Ruiz, K.; Tan, J.; Jayasinghe, T.N.; Khan, S.; Eroglu, E.; Adler, C.; Simpson, S.J.; Le Couteur, D.G.; Raubenheimer, D.; et al. A randomised clinical trial to investigate the effect of dietary protein sources on periodontal health. *Journal of Clinical Periodontology* **2022**, *49*, 388-400, doi:https://dx.doi.org/10.1111/jcpe.13587.
104. Elkan, A.C.; Sjöberg, B.; Kolsrud, B.; Ringertz, B.; Hafström, I.; Frostegård, J. Gluten-free vegan diet induces decreased LDL and oxidized LDL levels and raised atheroprotective natural antibodies against phosphorylcholine in patients with rheumatoid arthritis: A randomized study. *Arthritis Research and Therapy* **2008**, *10*, doi:10.1186/ar2388.
105. Elkoustaf, R.A.; Ismail, M.; Batiste, C.; Mercer, A.; Aldaas, O.; Newton, D.; Permanente, K. Diet and ischemic burden: A comparative analysis of a plant based regimen versus a mediterranean regimen in patients with coronary artery disease. *Journal of the American College of Cardiology* **2017**, *69*, 48, doi:10.1016/S0735-1097(17)33437-X.
106. Ellsworth, D.L.; Costantino, N.S.; Blackburn, H.L.; Engler, R.J.; Vernalis, M.N. Cardiac interventions differing in lifestyle modification intensity improve insulin resistance through changes in lipoprotein profiles. *Circulation* **2016**, *133*, 108, doi:https://doi.org/10.1161/circ.133.suppl_1.p108
107. Ellsworth, D.; Mamula, K.A.; Blackburn, H.L.; Engler, R.; Vernalis, M. Cardiac lifestyle interventions differing in dietary stringency improve insulin resistance through changes in lipoprotein profiles. *Journal of the American College of Cardiology* **2015**, *65*, A1450.
108. Essentia, H.; Blue Cross Blue Shield of Michigan, F. The Impact of Dietary Restriction of Animal Protein and Fat on Osteoarthritis. **2010**. https://ClinicalTrials.gov/show/NCT01939262
109. Fan, R.; Xu, M.; Wang, J.; Zhang, Z.; Chen, Q.; Li, Y.; Gu, J.; Cai, X.; Guo, Q.; Bao, L.; et al. Sustaining Effect of Intensive Nutritional Intervention Combined with Health Education on Dietary Behavior and Plasma Glucose in Type 2 Diabetes Mellitus Patients. *Nutrients* **2016**, *8*, 13, doi:https://dx.doi.org/10.3390/nu8090560.
110. Ferdowsian, H.R.; Barnard, N.D.; Hoover, V.J.; Katcher, H.I.; Levin, S.M.; Green, A.A.; Cohen, J.L. A multicomponent intervention reduces body weight and cardiovascular risk at a GEICO corporate site. *American journal of health promotion : AJHP* **2010**, *24*, 384-387, doi:10.4278/ajhp.081027-QUAN-255.
111. Fleming, J.; Kris-Etherton, P.; Petersen, K.; Baer, D. The dose-response effect of a mediterranean style diet with lean beef on lipids and lipoproteins. *Circulation* **2019**, *139*, doi:10.1161/circ.139.suppl_1.045.
112. Flynn, M.M.; Fenton, M. Comparison of weight loss with a National Cancer Institute diet and a plant-based olive oil diet in overweight women with invasive breast cancer. *Cancer Research* **2009**, *69*, doi:10.1158/0008-5472.SABCS-5080.
113. Gardner, C.D.; Coulston, A.; Chatterjee, L.; Rigby, A.; Spiller, G.; Farquhar, J.W. The effect of a plant-based diet on plasma lipids in hypercholesterolemic adults: a randomized trial. *Annals of Internal Medicine* **2005**, *142*, 725-733.
114. Garousi, N.; Tamizifar, B.; Pourmasoumi, M.; Feizi, A.; Askari, G.; Clark, C.C.T.; Entezari, M.H. Effects of lacto-ovo-vegetarian diet vs. standard-weight-loss diet on obese and overweight adults with non-alcoholic fatty liver disease: a randomised clinical trial. *Archives of Physiology & Biochemistry* **2023**, *129*, 975-983, doi:https://dx.doi.org/10.1080/13813455.2021.1890128.
115. Garousi, N.; Tamizifar, B.; Pourmasoumi, M.; Feizi, A.; Askari, G.; Clark, C.C.T.; Entezari, M.H. Effects of lacto-ovo-vegetarian diet vs. standard-weight-loss diet on obese and overweight adults with non-alcoholic fatty liver disease: a randomised clinical trial. *Archives of Physiology & Biochemistry* **2021**, 1-9, doi:https://dx.doi.org/10.1080/13813455.2021.1890128.
116. Gonciulea, A.R.; Sellmeyer, D. The effect of dietary protein source on serum lipids. *Endocrine Reviews* **2015**, *36*, i1–i1599, doi:https://doi.org/10.1093/edrv/36.supp.1
117. Gonciulea, A.R.; Sellmeyer, D.E. The effect of dietary protein source on serum lipids: Secondary data analysis from a randomized clinical trial. *Journal of Clinical Lipidology* **2017**, *11*, 46-54, doi:https://dx.doi.org/10.1016/j.jacl.2016.09.014.

118. Guagnano, M.T.; D'Angelo, C.; Caniglia, D.; Di Giovanni, P.; Celletti, E.; Sabatini, E.; Speranza, L.; Bucci, M.; Cipollone, F.; Paganelli, R. Improvement of Inflammation and Pain after Three Months' Exclusion Diet in Rheumatoid Arthritis Patients. *Nutrients* **2021**, *13*, doi:10.3390/nu13103535.
119. Gupta, S.; Bishnoi, R. A Study of Body Mass Index According to Food Habits Among the Woman Aged 20-50 Years. *European Journal of Molecular and Clinical Medicine* **2023**, *10*, 1694-1699.
120. Haag, L.; Richardson, J.; Haig, C.; Cunningham, Y.; Fraser, H.; Brosnahan, N.; Ibbotson, T.; Ormerod, J.; White, C.; McIntosh, E.; et al. The Remote Diet Intervention to REduce long Covid symptoms Trial (ReDIRECT) - dietary profile of the study participants at baseline. *Proceedings of the Nutrition Society* **2023**, *82*, E226, doi:10.1017/S0029665123002963.
121. Hakala, P.; Karvetti, R.L. Weight reduction on lactovegetarian and mixed diets. Changes in weight, nutrient intake, skinfold thicknesses and blood pressure. *European Journal of Clinical Nutrition* **1989**, *43*, 421-430.
122. Hartmann, A.M.; D'Urso, M.; Dell'Oro, M.; Koppold, D.A.; Steckhan, N.; Michalsen, A.; Kandil, F.I.; Kessler, C.S. Post Hoc Analysis of a Randomized Controlled Trial on Fasting and Plant-Based Diet in Rheumatoid Arthritis (NutriFast): Nutritional Supply and Impact on Dietary Behavior. *Nutrients* **2023**, *15*, 07, doi:https://dx.doi.org/10.3390/nu15040851.
123. Hartmann, A.M.; Dell'Oro, M.; Spoo, M.; Fischer, J.M.; Steckhan, N.; Jeitler, M.; Haupl, T.; Kandil, F.I.; Michalsen, A.; Koppold-Liebscher, D.A.; et al. To eat or not to eat-an exploratory randomized controlled trial on fasting and plant-based diet in rheumatoid arthritis (NutriFast-Study). *Frontiers in Nutrition* **2022**, *9*, 1030380, doi:https://dx.doi.org/10.3389/fnut.2022.1030380.
124. Haub, M.D.; Wells, A.M.; Campbell, W.W. Beef and soy-based food supplements differentially affect serum lipoprotein-lipid profiles because of changes in carbohydrate intake and novel nutrient intake ratios in older men who resistive-train. *Metabolism: Clinical & Experimental* **2005**, *54*, 769-774.
125. Hentges, D.J. Fecal flora of volunteers on controlled diets. *American Journal of Clinical Nutrition* **1978**, *31*, S123-S124, doi:10.1093/ajcn/31.10.s123.
126. Hrachovinová, T.; Kahleová, H.; Hackerová, P.; Pelikánová, T. Changes in eating behavior, depression and quality of life in patients with type 2 diabetes after a 3-months-intervention with low-fat vegetarian diet and conventional diabetic diet. *Diabetologia* **2009**, *52*, S392, doi:10.1007/s00125-009-1445-1.
127. Huber, H.; Ashton, N.J.; Weinhold, L.; Schmid, M.; Coenen, M.; Stoffel-Wagner, B.; Blennow, K.; Stehle, P.; Zetterberg, H.; Simon, M.C. Plasma Biomarker Concentrations of Alzheimer's Disease Are Associated with Insulin Resistance-Results from a Pilot RCT in Adults with Diabetes Risk. *Diabetes* **2023**, *72*, doi:10.2337/db23-1518-P.
128. Huber, H.; Mantri, A.; Seel, W.; Schieren, A.; Stoffel-Wagner, B.; Coenen, M.; Nöthen, M.; Schmid, M.; Krawitz, P.; Stehle, P.; et al. A 6-week intervention with a nordic-like dietary pattern increased gut microbial diversity only in obese subjects showing a diet-improved lipid metabolism. *Clinical Nutrition ESPEN* **2021**, *46*, S774-S775, doi:10.1016/j.clnesp.2021.09.654.
129. Huber, H.; Mantri, A.; Seel, W.; Stoffel-Wagner, B.; Coenen, M.; Nothen, M.; Schmid, M.; Weinhold, L.; Krawitz, P.; Holst, J.J.; et al. Impact of diet and body mass index on parameters of the gut-brain axis in obese subjects: results from a dietary intervention trial. *Clinical nutrition ESPEN* **2023**, *54*, 718, doi:10.1016/j.clnesp.2022.09.754.
130. Huber, H.; Stoffel-Wagner, B.; Coenen, M.; Weinhold, L.; Schmid, M.; Stehle, P.; Simon, M.C. Short-term impact of a vegetarian and the nordic diet on metabolic risk factors: a human intervention study in adults with a risk phenotype for cardio metabolic diseases. *Clinical Nutrition ESPEN* **2021**, *46*, S562, doi:10.1016/j.clnesp.2021.09.065.
131. Huber, H.; Stoffel-Wagner, B.; Coenen, M.; Weinhold, L.; Schmid, M.; Stehle, P.; Simon, M.C. Impact of vegetarian and the nordic diet on glucose metabolism: A human intervention study. *Diabetes* **2021**, *70*, doi:10.2337/db21-251-OR.
132. Hunt, J.R.; Matthys, L.A.; Johnson, L.K. Zinc absorption, mineral balance, and blood lipids in women consuming controlled lactoovovegetarian and omnivorous diets for 8 wk. *American Journal of Clinical Nutrition* **1998**, *67*, 421-430.
133. Hyder, J.A.; Thomson, C.A.; Natarajan, L.; Madlensky, L.; Pu, M.; Emond, J.; Kealey, S.; Rock, C.L.; Flatt, S.W.; Pierce, J.P. Adopting a plant-based diet minimally increased food costs in WHEL Study. *American Journal of Health Behavior* **2009**, *33*, 530-539.
134. Imperial College, L. Application of New Technologies and Tools to Nutrition Research. **2012**. https://ClinicalTrials.gov/show/NCT01684917
135. Irct20140208016529N; Vice Chancellor for Research, I.U.o.M.S. the effect of lacto ovo vegetarian and normal diets in nonalcoholic fatty liver disease. **2018**. http://en.irct.ir/trial/28492

136. Irct20150909023957N; Vice-Chancellor in Research Affairs -Medical University of Isfahan, Y. The effect of replacing animal-based proteins with plant-based proteins on metabolic syndrome treatment. **2022**. https://irct.behdasht.gov.ir/trial/63377
137. Irct20190427043387N. MIND diet and cognitive function. http://www.who.int/trialsearch/Trial2.aspx?TrialID=IRCT20190427043387N1 **2019**.
138. Irct20190717044244N; Ahvaz University of Medical, S. Fasting mimicking diet compared with low calorie diet among premenopausal obese women. **2019**. http://en.irct.ir/trial/40881
139. Irct20230821059213N; Vice chancellor for research, S.B.U.o.M.s.-S.o.N.N. The effect of time-restricted feeding (TRF) combined with a Lacto-Ovo-Vegetarian (LOV) diet on controlling of non-alcoholic fatty liver disease. **2023**. http://en.irct.ir/trial/72231
140. Isrctn; Karl und Veronica Carstens-Stiftung, Y. BrainFit-Nutrition: Intervention study for people with mild cognitive impairment using computerised cognitive training tools and a nutrition intervention. **2021**. https://www.isrctn.com/ISRCTN10560738
141. Isrctn; Universidade Católica Portuguesa, N.; Universidade do, P.; Sense Test, S.d.E.d.A.S.a.P.A.L.A.C.d.É.e.T.C.S.e.H.r.C. ChangeEat! – Household dinners with alternative proteins to reduce red and processed meat consumption in young adult couples. **2023**. https://www.isrctn.com/ISRCTN53814211
142. Isrctn; University Hospital of Zurich, Y. Determining the effects of vegan and omnivorous diets on the effects of exercise and the intestinal bacteria population in ultra-marathoners. **2020**. https://www.isrctn.com/ISRCTN69541705
143. Jenkins, D.J.; Jones, P.J.; Abdullah, M.M.; Lamarche, B.; Faulkner, D.; Patel, D.; Sahye-Pudaruth, S.; Paquette, M.; Bashyam, B.; Pichika, S.C.; et al. Low-carbohydrate vegan diets in diabetes for weight loss and sustainability: a randomized controlled trial. *The American journal of clinical nutrition* **2022**, *116*, 1240-1250, doi:10.1093/ajcn/nqac203.
144. Jenkins, D.J.; Kendall, C.W.; Faulkner, D.A.; Kemp, T.; Marchie, A.; Nguyen, T.H.; Wong, J.M.; de Souza, R.; Emam, A.; Vidgen, E.; et al. Long-term effects of a plant-based dietary portfolio of cholesterol-lowering foods on blood pressure. *European Journal of Clinical Nutrition* **2008**, *62*, 781-788, doi:10.1038/sj.ejcn.1602768.
145. Johansson, G.; Widerström, L. Change from mixed diet to lactovegetarian diet: influence on IgA levels in blood and saliva. *Scandinavian journal of dental research* **1994**, *102*, 350-354.
146. Johnstone, A.M.; Fyfe, C.; Horgan, G.W. Weight loss and appetite with vegetarian and meat rich high protein diets. *Obesity Reviews* **2010**, *11*, 41, doi:10.1111/j.1467-789X.2010.00763-4.x.
147. Jprn, U.; Factory of, H.; Sports Science, U.o.T.Y. Effects of a dietary weight loss program including the replacement of animal proteins with plant proteins on phosphorus metabolism: A randomized controlled trial. **2022**. https://center6.umin.ac.jp/cgi-open-bin/ctr_e/ctr_view.cgi?recptno=R000054804
148. Jprn, U.; Team, I.B.D.R.P. Effect of a plant-based diet in inflammatory bowel disease. **2016**. https://center6.umin.ac.jp/cgi-open-bin/ctr_e/ctr_view.cgi?recptno=R000022040
149. Jprn, U.; Asama General, H. Efficacy of making one meal a vegetarian diet : a randomized, open-label study. **2018**. https://center6.umin.ac.jp/cgi-open-bin/ctr_e/ctr_view.cgi?recptno=R000036446
150. Jprn, U.; Kagome Co, L. Evaluation of the effect of plant-based food on influenza virus infection: a randomized, placebo-controlled, double-blind, parallel group comparison study. **2019**. https://www.isrctn.com/ISRCTN69541705
151. Jprn, U. Effects of a dietary weight loss program including the replacement of animal proteins with plant proteins on phosphorus metabolism: a randomized controlled trial. https://trialsearch.who.int/Trial2.aspx?TrialID=JPRN-UMIN000048081 **2022**.
152. Kafyra, M.; Kalafati, I.P.; Katsareli, E.A.; Lambrinou, S.; Varlamis, I.; Kaliora, A.C.; Dedoussis, G.V. The iMPROVE Study; Design, Dietary Patterns, and Development of a Lifestyle Index in Overweight and Obese Greek Adults. *Nutrients* **2021**, *13*, 03, doi:https://dx.doi.org/10.3390/nu13103495.
153. Kahleova, H. The Effect of a Dietary Intervention on Insulin Requirements in Type 1 Diabetes-A 12-Week Randomized Clinical Trial. *Diabetes* **2023**, *72*, doi:10.2337/db23-606-P.
154. Kahleova, H.; Berrien-Lopez, R.; Holtz, D.; Green, A.; Sheinberg, R.; Gujral, H.; Holubkov, R.; Barnard, N.D. Nutrition for Hospital Workers During a Crisis: Effect of a Plant-Based Dietary Intervention on Cardiometabolic Outcomes and Quality of Life in Healthcare Employees During the COVID-19 Pandemic. *American Journal of Lifestyle Medicine* **2022**, *16*, 399-407, doi:https://dx.doi.org/10.1177/15598276211050339.
155. Kahleova, H.; Hill, M.; Pelikanova, T. Vegetarian vs. conventional diabetic diet - A 1-year follow-up. *Cor et Vasa* **2014**, *56*, e140-e144, doi:10.1016/j.crvasa.2013.12.004.
156. Kahleova, H.; Klementova, M.; Herynek, V.; Skoch, A.; Herynek, S.; Hill, M.; Mari, A.; Pelikanova, T. The Effect of a Vegetarian vs Conventional Hypocaloric Diabetic Diet on Thigh Adipose Tissue Distribution in

Subjects with Type 2 Diabetes: A Randomized Study. *Journal of the American College of Nutrition* **2017**, *36*, 364-369, doi:https://dx.doi.org/10.1080/07315724.2017.1302367.

157. Kahleova, H.; Tintera, J.; Thieme, L.; Veleba, J.; Klementova, M.; Kudlackova, M.; Malinska, H.; Oliyarnyk, O.; Markova, I.; Haluzik, M.; et al. A plant-based meal affects thalamus perfusion differently than an energy- and macronutrient-matched conventional meal in men with type 2 diabetes, overweight/obese, and healthy men: a three-group randomized crossover study. *Clinical nutrition (Edinburgh, Scotland)* **2020**, 40, 1822-1833, doi:10.1016/j.clnu.2020.10.005.

158. Kahleova, H.; Matoulek, M.; Bratova, M.; Malinska, H.; Kazdova, L.; Hill, M.; Pelikanova, T. Vegetarian diet-induced increase in linoleic acid in serum phospholipids is associated with improved insulin sensitivity in subjects with type 2 diabetes. *Nutrition & Diabetes* **2013**, *3*, e75, doi:https://dx.doi.org/10.1038/nutd.2013.12.

159. Kahleova, H.; Matoulek, M.; Hill, M.; Pelikanova, T. Vegetarian diet increases resting energy expenditure more than conventional diet in subjects with type 2 diabetes after aerobic exercise. *Diabetes* **2011**, *60*, A217, doi:10.2337/db11-716-867.

160. Kahleova, H.; Matoulek, M.; Kazdova, L.; Hill, M.; Pelikanova, T. The effect of vegetarian diet on fatty acid composition of serum phopholipids and the association with insulin sensitivity and visceral fat in subjects with type 2 diabetes. *Diabetologia* **2011**, *54*, S359-S360, doi:10.1007/s00125-011-2276-4.

161. Kahleova, H.; Matoulek, M.; Malinska, H.; Oliyarnik, O.; Kazdova, L.; Neskudla, T.; Skoch, A.; Hajek, M.; Hill, M.; Kahle, M.; et al. Vegetarian diet improves insulin resistance and oxidative stress markers more than conventional diet in subjects with Type 2 diabetes. *Diabetic Medicine* **2011**, *28*, 549-559, doi:10.1111/j.1464-5491.2010.03209.x.

162. Kahleova, H.; Matoulek, M.; Malinska, H.; Oliyarnik, O.; Kazdova, L.; Pelikánová, T. Vegetarian diet improves plasma concentrations of adipokines and oxidative stress markers more than conventional diabetic diet in subjects with type 2 diabetes. *Diabetologia* **2010**, *53*, S380, doi:10.1007/s00125-010-1872-z.

163. Kahleova, H.; Neskudla, T.; Pelikanova, T. Does a low-fat vegetarian diet improve insulin resistance and beta cell function in individuals with type 2 diabetes? *Diabetologia* **2009**, *52*, S321-S322, doi:10.1007/s00125-009-1445-1.

164. Kahleova, H.; Sutton, M.; Maracine, C.; Nichols, D.; Monsivais, P.; Holubkov, R.; Barnard, N.D. Vegan Diet and Food Costs Among Adults With Overweight: A Secondary Analysis of a Randomized Clinical Trial. *JAMA Network Open* **2023**, *6*, e2332106, doi:https://dx.doi.org/10.1001/jamanetworkopen.2023.32106.

165. Kahleova, H.; Tintera, J.; Thieme, L.; Veleba, J.; Klementova, M.; Kudlackova, M.; Malinska, H.; Oliyarnyk, O.; Markova, I.; Haluzik, M.; et al. A plant-based meal affects thalamus perfusion differently than an energy- and macronutrient-matched conventional meal in men with type 2 diabetes, overweight/obese, and healthy men: A three-group randomized crossover study. *Clinical Nutrition* **2021**, *40*, 1822-1833, doi:https://dx.doi.org/10.1016/j.clnu.2020.10.005.

166. Kahleova, H.; Tonstad, S.; Rosmus, J.; Fisar, P.; Mari, A.; Hill, M.; Pelikanova, T. The effect of a vegetarian versus conventional hypocaloric diet on serum concentrations of persistent organic pollutants in patients with type 2 diabetes. *Nutrition Metabolism & Cardiovascular Diseases* **2016**, *26*, 430-438, doi:https://dx.doi.org/10.1016/j.numecd.2016.01.008.

167. Kahleova, H.; Tura, A.; Hill, M.; Holubkov, R.; Barnard, N.D. A Plant-Based Dietary Intervention Improves Beta-Cell Function and Insulin Resistance in Overweight Adults: A 16-Week Randomized Clinical Trial. *Nutrients* **2018**, *10*, 9, doi:https://dx.doi.org/10.3390/nu10020189.

168. Kahleova, H.; Tura, A.; Klementova, M.; Thieme, L.; Haluzik, M.; Pavlovicova, R.; Hill, M.; Pelikanova, T. A plant-based meal stimulates incretin and insulin secretion more than an energy-and macronutrient-matched standard meal in type 2 diabetes: A randomized crossover study. *Nutrients* **2019**, *11*, doi:10.3390/nu11030486.

169. Kahleova, H.; Znayenko-Miller, T.; Smith, K.; Holubkov, R.; Barnard, N. The effect of a dietary intervention on insulin requirements in type 1 diabetes: a 12-week randomised clinical trial. *Diabetologia* **2023**, *66*, S103, doi:10.1007/s00125-023-05969-6.

170. Karlsson, J.; Hallgren, P.; Kral, J.; Lindroos, A.K.; Sjostrom, L.; Sullivan, M. Predictors and effects of long-term dieting on mental well-being and weight loss in obese women. *Appetite* **1994**, *23*, 15-26.

171. Kct; Kyungpook National, U. Evaluation of clinical effects and persistent organic pollutants (POPs)-related mechanisms of vegan diet among patients with diabetes. **2016**.
http://cris.nih.go.kr/cris/en/search/search_result_st01.jsp?seq=5905

172. Kestin, M.; Rouse, I.L.; Correll, R.A.; Nestel, P.J. Cardiovascular disease risk factors in free-living men: comparison of two prudent diets, one based on lactoovovegetarianism and the other allowing lean meat. *American Journal of Clinical Nutrition* **1989**, *50*, 280-287.

173. Kies, C.V. Mineral utilization of vegetarians: impact of variation in fat intake. *American Journal of Clinical Nutrition* **1988**, *48*, 884-887, doi:https://dx.doi.org/10.1093/ajcn/48.3.884.
174. Kim, M.S.; Hwang, S.S.; Park, E.J.; Bae, J.W. Strict vegetarian diet improves the risk factors associated with metabolic diseases by modulating gut microbiota and reducing intestinal inflammation. *Environmental Microbiology Reports* **2013**, *5*, 765-775, doi:10.1111/1758-2229.12079.
175. Kim, S.H.; Cho, S.W.; Hwang, S.S.; Ahn, M.; Lee, D.; Kang, S.W. Increased whole grain, fruits and vegetable intake reduced oxidative stress in high school students. *Korean journal of nutrition* **2012**, *45*, 452-461, doi:10.4163/kjn.2012.45.5.452.
176. Klementova, M.; Thieme, L.; Haluzik, M.; Pavlovicova, R.; Hill, M.; Pelikanova, T.; Kahleova, H. A Plant-Based Meal Increases Gastrointestinal Hormones and Satiety More Than an Energy- and Macronutrient-Matched Processed-Meat Meal in T2D, Obese, and Healthy Men: A Three-Group Randomized Crossover Study. *Nutrients* **2019**, *11*, 12, doi:https://dx.doi.org/10.3390/nu11010157.
177. Koebnick, C.; Plank-Habibi, S.; Wirsam, B.; Gruendel, S.; Hahn, A.; Meyer-Kleine, C.; Leitzmann, C.; Zunft, H.J. Double-blind, randomized feedback control fails to improve the hypocholesterolemic effect of a plant-based low-fat diet in patients with moderately elevated total cholesterol levels. *European Journal of Clinical Nutrition* **2004**, *58*, 1402-1409.
178. Krenek, A.; Mathews, A.; Guo, J.; Chung, S.; Courville, A.; Aggarwal, M. Recipe for Heart Health: a Randomized Crossover Trial Evaluating the Effects of Extra Virgin Olive Oil Within a Vegan Diet on Cardiometabolic Risk Factors. *Current developments in nutrition* **2023**, *7*, doi:10.1016/j.cdnut.2023.100155.
179. Kudláčková, M.; Kahleová, H.; Tintěra, J.; Klementová, M.; Thieme, L.; Veleba, J.; Malinská, H.; Mráz, M.; Haluzik, M.; Pavlovičová, R.; et al. A plant-based meal affects thalamus perfusion differently than an energy- and macronutrient-matched conventional meal in type 2 diabetes, obese, and healthy men. *Diabetologia* **2020**, *63*, S265, doi:10.1007/s00125-020-05221-5.
180. Landry, M.J.; Ward, C.P.; Cunanan, K.M.; Durand, L.R.; Perelman, D.; Robinson, J.L.; Hennings, T.; Koh, L.; Dant, C.; Zeitlin, A.; et al. Cardiometabolic Effects of Omnivorous vs Vegan Diets in Identical Twins: A Randomized Clinical Trial. *JAMA Network Open* **2023**, *6*, e2344457, doi:https://dx.doi.org/10.1001/jamanetworkopen.2023.44457.
181. Lederer, A.K.; Hannibal, L.; Hettich, M.; Behringer, S.; Spiekerkoetter, U.; Steinborn, C.; Gründemann, C.; Zimmermann-Klemd, A.M.; Müller, A.; Simmet, T.; et al. Vitamin B12 Status Upon Short-Term Intervention with a Vegan Diet-A Randomized Controlled Trial in Healthy Participants. *Nutrients* **2019**, *11*, doi:10.3390/nu11112815.
182. Lee, J.K.; Kim, H. Effects of the lifestyle modification program to reduce serum lipoprotein(a) and other cardiovascular risk factors in Korean college women. *Gazzetta Medica Italiana Archivio per le Scienze Mediche* **2018**, *177*, 468-474, doi:10.23736/S0393-3660.17.03592-6.
183. Lee, K.S.; Lee, J.K.; Yeun, Y.R. Effects of a 10-Day Intensive Health Promotion Program Combining Diet and Physical Activity on Body Composition, Physical Fitness, and Blood Factors of Young Adults: A Randomized Pilot Study. *Medical Science Monitor* **2017**, *23*, 1759-1767.
184. Lee, Y.M.; Kim, S.A.; Lee, I.K.; Kim, J.G.; Park, K.G.; Jeong, J.Y.; Jeon, J.H.; Shin, J.Y.; Lee, D.H. Effect of a brown rice based vegan diet and conventional diabetic diet on glycemic control of patients with type 2 diabetes: A 12-week randomized clinical trial. *PLoS ONE [Electronic Resource]* **2016**, *11*, doi:10.1371/journal.pone.0155918.
185. Leslie, W.S.; Lean, M.E.; Baillie, H.M.; Hankey, C.R. Weight management: a comparison of existing dietary approaches in a work-site setting. *International Journal of Obesity & Related Metabolic Disorders: Journal of the International Association for the Study of Obesity* **2002**, *26*, 1469-1475.
186. Link, V.M.; Hall, K.; Belkaid, Y. Highly controlled nutritional interventions uncover defined immune and microbiome responses in human. *Journal of Immunology* **2023**, *210*, doi:10.4049/jimmunol.210.Supp.66.01.
187. Lisevick, A.; Cartmel, B.; Harrigan, M.; Li, F.; Sanft, T.; Fogarasi, M.; Irwin, M.L.; Ferrucci, L.M. Effect of the lifestyle, exercise, and nutrition (Lean) study on long-term weight loss maintenance in women with breast cancer. *Nutrients* **2021**, *13*, doi:10.3390/nu13093265.
188. Lowe, D.A.; Wu, N.; Rohdin-Bibby, L.; Moore, A.H.; Kelly, N.; Liu, Y.E.; Philip, E.; Vittinghoff, E.; Heymsfield, S.B.; Olgin, J.E.; et al. Effects of time-restricted eating on weight loss and other metabolic parameters in women and men with overweight and obesity: the TREAT randomized clinical trial. *JAMA Internal Medicine* **2020**, *180*, doi:10.1001/jamainternmed.2020.4153.
189. Lukaszuk, J.M.; Robertson, R.J.; Arch, J.E.; Moore, G.E.; Yaw, K.M.; Kelley, D.E.; Rubin, J.T.; Moyna, N.M. Effect of creatine supplementation and a lacto-ovo-vegetarian diet on muscle creatine concentration. *International Journal of Sport Nutrition & Exercise Metabolism* **2002**, *12*, 336-348.

190. Maastricht University Medical, C.; National Cattlemen's Beef, A.; Vion Food, G. The Impact of a Whole-food Animal-based Versus Plant-based Protein Rich Meal on Muscle Protein Synthesis. **2021**. https://ClinicalTrials.gov/show/NCT05151887
191. Malek, E.; Sanati, F.; Lauren, A.; Metheny, L.; Gallogly, M.; Retuerto, M.; Ghannoum, M. Mycobiome supporting diet to reduce gastrointestinal (GI)toxicity associated with autologous stem cell transplant (ASCT) for patients with multiple myeloma (MM). *Blood* **2021**, *138*, 3948, doi:10.1182/blood-2021-148226.
192. Margetts, B.M.; Beilin, L.J.; Vandongen, R.; Armstrong, B.K. A randomized controlled trial of the effect of dietary fibre on blood pressure. *Clinical Science* **1987**, *72*, 343-350.
193. Marniemi, J.; Seppanen, A.; Hakala, P. Long-term effects on lipid metabolism of weight reduction on lactovegetarian and mixed diet. *International Journal of Obesity* **1990**, *14*, 113-125.
194. Martineta, M.; Agustina, R.; Febriyanti, E.; Putri, M.; Purnamasari, D. A balanced-sustainable calorie-restricted diet effect using "eats up"application on waist circumference and inflammatory marker among Indonesian obese women: Randomized clinical trial. *Annals of Nutrition and Metabolism* **2019**, *75*, 62, doi:10.1159/000501751.
195. Masarei, J.R.; Rouse, I.L.; Lynch, W.J.; Robertson, K.; Vandongen, R.; Beilin, L.J. Effects of a lacto-ovo vegetarian diet on serum concentrations of cholesterol, triglyceride, HDL-C, HDL2-C, HDL3-C, apoprotein-B, and Lp(a). *American Journal of Clinical Nutrition* **1984**, *40*, 468-478.
196. McDougall, J.; Bruce, B.; Spiller, G.; Westerdahl, J.; McDougall, M. Effects of a very low-fat, vegan diet in subjects with rheumatoid arthritis. *Journal of Alternative and Complementary Medicine* **2002**, *8*, 71-75, doi:10.1089/107555302753507195.
197. McGill University Health Centre/Research Institute of the McGill University Health, C.; João Pedro Ferreira, M.D. Plant-Based Meat vs Animal "Red" Meat Trial. **2020**. https://ClinicalTrials.gov/show/NCT04510324
198. Memorial Sloan Kettering Cancer, C. A Study of a Plant-Based Diet in People With Monoclonal Gammopathy of Undetermined Significance (MGUS) or Smoldering Multiple Myeloma (SMM). **2021**. https://ClinicalTrials.gov/show/NCT04920084
199. Memorial Sloan Kettering Cancer, C.; American Cancer Society, I. A Study of the Body's Response to Exercise and a Plant-Based Diet in Overweight Postmenopausal Women With Breast Cancer. **2020**. https://ClinicalTrials.gov/show/NCT04298086
200. Mia, F.B.; Vorster, H.H. Coronary heart disease risk factors in Indian adolescents - The role of diet. *Cardiovascular Journal of Southern Africa* **2000**, *11*, 68-75.
201. Mirela, Z.; University of Split, S.o.M. Vitamin B12 Pregnancy Supplementation. **2011**. https://ClinicalTrials.gov/show/NCT03522428
202. Mishra, S.; Barnard, N.D.; Gonzales, J.; Xu, J.; Agarwal, U.; Levin, S. Nutrient intake in the GEICO multicenter trial: the effects of a multicomponent worksite intervention. *European Journal of Clinical Nutrition* **2013**, *67*, 1066-1071, doi:https://dx.doi.org/10.1038/ejcn.2013.149.
203. Mishra, S.; Barnard, N.D.; Xu, J.; Trap, C. A plant-based diet reduces body weight and cardiovascular risk: The geico multicenter trial. *Diabetes* **2012**, *61*, A192, doi:10.2337/db12-656-835.
204. Mishra, S.; Xu, J.; Agarwal, U.; Gonzales, J.; Levin, S.; Barnard, N.D. A multicenter randomized controlled trial of a plant-based nutrition program to reduce body weight and cardiovascular risk in the corporate setting: the GEICO study. *European Journal of Clinical Nutrition* **2013**, *67*, 718-724, doi:https://dx.doi.org/10.1038/ejcn.2013.92.
205. Mocanu, C.A.; Ghica, T.D.; Simionescu, T.P.; Mocanu, A.E.; Mircescu, G.; Garneata, L. POS-262 Low-Protein Diets in Chronic Kidney Disease. *Kidney International Reports* **2022**, *7*, S115-S116, doi:10.1016/j.ekir.2022.01.281.
206. Mohammad, A.M.; Tuller, E.R.; Dyroff, S.; Patterson, E.L.; Hunter, M.I. Weight intervention in endometrial cancer: A pilot program. *Gynecologic Oncology* **2019**, *154*, 169, doi:10.1016/j.ygyno.2019.04.395.
207. Moore, W.J.; McGrievy, M.E.; Turner-McGrievy, G.M. Dietary adherence and acceptability of five different diets, including vegan and vegetarian diets, for weight loss: The New DIETs study. *Eating Behaviors* **2015**, *19*, 33-38, doi:https://dx.doi.org/10.1016/j.eatbeh.2015.06.011.
208. Morgan-Bathke, M.E.; Jensen, M.D. Preliminary evidence for reduced adipose tissue inflammation in vegetarians compared with omnivores. *Nutrition Journal* **2019**, *18*, doi:10.1186/s12937-019-0470-2.
209. Murphy, K.; Dyer, K.; Hyde, B.; Davis, C.; Hodgson, J.; Woodman, R. Considerations for the adoption of a Mediterranean diet beyond the Mediterranean Sea: Lessons from Australian trials. *Proceedings of the Nutrition Society* **2020**, *79*, doi:10.1017/S0029665120000920.

210. Muti, P.; Awad, A.B.; Schunemann, H.; Fink, C.S.; Hovey, K.; Freudenheim, J.L.; Wu, Y.W.; Bellati, C.; Pala, V.; Berrino, F. A plant food-based diet modifies the serum beta-sitosterol concentration in hyperandrogenic postmenopausal women. *Journal of Nutrition* **2003**, *133*, 4252-4255.
211. Najafabadi, M.S.; Moludi, J.; Salimi, Y.; Saber, A. A comparison of the portfolio low-carbohydrate diet and the ketogenic diet in overweight and obese women with polycystic ovary syndrome: study protocol for a randomized controlled trial. *Trials [Electronic Resource]* **2023**, *24*, 509, doi:https://dx.doi.org/10.1186/s13063-023-07569-6.
212. Nct. Plant-Based, American Heart Assoc. or Mediterranean Diets In 9-18 yo With BMI >95%, Cholesterol >169 and Their Parents. https://clinicaltrials.gov/show/NCT02857543 **2016**.
213. Nct. Olive Oil v Prostate Cancer Foundation Diet for Treatment of Prostate Cancer. https://clinicaltrials.gov/show/NCT03084913 **2017**.
214. Nct. New DIETs: new Dietary Interventions Enhancing the Treatment for Weight Loss. https://clinicaltrials.gov/show/NCT01742572 **2012**.
215. Nct. Mediterranean Lifestyle Intervention in Patients With Obstructive Sleep Apnea. https://clinicaltrials.gov/show/NCT02515357 **2015**.
216. Nct. Mediterranean Diet, Circuit Resistance Training, Empagliflozin in Elderly With Type 2 Diabetes: a Study Protocol. https://clinicaltrials.gov/show/NCT03560375 **2018**.
217. Nct. Low-Fat Vegan Diet Versus a Mediterranean Diet on Body Weight. https://clinicaltrials.gov/show/NCT03698955 **2018**.
218. Nct. Impact of a Mediterranean Diet on Cardiovascular Disease Risk Factors: a Randomized Clinical Trial. https://clinicaltrials.gov/ct2/show/NCT06113484 **2023**.
219. Nct. IIT Whole-Food Plant-Based Diet (WFPBD). https://clinicaltrials.gov/show/NCT05471414 **2022**.
220. Nct. Healthy Eating for Reproductive Health (HERHealth). https://clinicaltrials.gov/show/NCT01509066 **2012**.
221. Nct. Fighting Immunosenescence and Promoting Immunity by a Fasting-mimicking Diet Elderly. https://clinicaltrials.gov/show/NCT04928963 **2021**.
222. Nct. Fasting-mimicking Diet Combined With Traditional Chinese Medicine for Phlegm-dampness Type Obesity Patients. https://clinicaltrials.gov/show/NCT04261764 **2020**.
223. Nct. Fasting-mimicking Diet and Periodontitis (FMD). https://clinicaltrials.gov/ct2/show/NCT06074861 **2023**.
224. Nct. Ectopic Fat in Singaporean Women - the Culprit Leading to Gestational Diabetes, Metabolic Syndrome, and Type 2 Diabetes (TANGO Study). https://clinicaltrials.gov/show/NCT05259475 **2022**.
225. Nct. Dietary Management of Gestational Diabetes. https://clinicaltrials.gov/show/NCT03681054 **2018**.
226. Nct. Complex Effects of Dietary Manipulation on Metabolic Function, Inflammation and Health. https://clinicaltrials.gov/show/NCT02706262 **2016**.
227. Nct; Physicians Committee for Responsible, M. Plant-Based Dietary Intervention in Type 2 Diabetes. **2006**. https://clinicaltrials.gov/study/NCT00276939
228. Nct; University of, P. Preference and Vegetarian Diet in Weight Loss Treatment. **2006**. https://clinicaltrials.gov/study/NCT00330629
229. Nct; Institute for, C.; Experimental, M. Does a Low-Fat Vegetarian Diet Improve Insulin Resistance in Individuals With Type 2 Diabetes? **2008**. https://clinicaltrials.gov/study/NCT00883038
230. Nct; Physicians Committee for Responsible, M. Practice Based Nutrition Intervention. **2010**. https://clinicaltrials.gov/study/NCT01222429
231. Nct; National Institute for, H.; Welfare, F.Y.; Academy of, F.; University of, O. Health Effects of a Nordic Diet Rich in Plant-based Foods and Fish. **2011**. https://clinicaltrials.gov/study/NCT01412346
232. Nct; Physicians Committee for Responsible, M. A Nutritional Intervention for Arthritis. **2012**. https://clinicaltrials.gov/study/NCT01544101
233. Nct; Physicians Committee for Responsible, M. A Nutritional Intervention for Migraines. **2012**. https://clinicaltrials.gov/study/NCT01547494
234. Nct; University of British Columbia, Y. Food Security and Nutrition in Rural Cambodia. **2012**. https://www.clinicaltrials.gov/study/NCT01593423
235. Nct; Physicians Committee for Responsible, M. A Nutritional Intervention for Diabetic Neuropathy. **2012**. https://clinicaltrials.gov/study/NCT01690962
236. Nct; Physicians Committee for Responsible, M. A Nutritional Intervention for Migraines-2. **2012**. https://clinicaltrials.gov/study/NCT01699009
237. Nct; Physicians Committee for Responsible, M. Practice Based Nutrition Intervention-2. **2012**. https://clinicaltrials.gov/study/NCT01700868

238. Nct; University of South, C. Healthy Eating for Reproductive Health: Greenville. **2012**. https://clinicaltrials.gov/study/NCT01716429
239. Nct. Low Fat Vegan or American Heart Association Diets & Cardiovascular Risk in Obese 9-18 y.o. With Elevated Cholesterol. https://clinicaltrials.gov/show/NCT01817491 **2013**.
240. Nct; Physicians Committee for Responsible, M. Plant-Based Dietary Intervention in Type 2 Diabetes-2. **2013**. https://clinicaltrials.gov/study/NCT01931631
241. Nct; Physicians Committee for Responsible, M. A Nutritional Intervention for Diabetic Neuropathy (WCCR-DN2). **2013**. https://clinicaltrials.gov/study/NCT01953757
242. Nct; University of, A. Vegetarian High Protein Weight Loss Diets. **2014**. https://clinicaltrials.gov/study/NCT02080325
243. Nct; New York University School of, M. Vegan vs AHA Diet on Inflammation and Glucometabolic Profile in Patients With CAD. **2014**. https://clinicaltrials.gov/study/NCT02135939
244. Nct; Tel-Aviv Sourasky Medical Center, Y. The Effect of Plant-based Diets (Vegetarian and Vegan) on Endothelial Function and Atherogenic Parameters. **2014**. https://clinicaltrials.gov/study/NCT02153138
245. Nct; Diabetes Foundation, I. High Protein Atkin's Complete (Lacto) VEgetaRian Diet (PACER) and Weight Loss. **2015**. https://clinicaltrials.gov/study/NCT02562209
246. Nct; Physicians Committee for Responsible, M. A Nutritional Intervention in Police Officers. **2016**. https://clinicaltrials.gov/study/NCT02651480
247. Nct; Azienda Ospedaliera Specializzata in Gastroenterologia Saverio de, B. Lacto-ovo-vegetarian Diet Riched in Omega-3 Fatty Acids in Menopausal Women. **2016**. https://clinicaltrials.gov/study/NCT02816814
248. Nct; Region Örebro, C. Vegetarian Diet in Patients With Ischemic Heart Disease. **2016**. https://clinicaltrials.gov/study/NCT02942628
249. Nct; University of, R. A Whole-food, Plant-Based Nutrition Intervention in Women With Metastatic Breast Cancer. **2017**. https://clinicaltrials.gov/study/NCT03045289
250. Nct; Texas Woman's, U. Effects of a 4-week Raw, Plant-based Diet on Anthropometric and Cardiovascular Risk Factors. **2017**. https://clinicaltrials.gov/study/NCT03134235
251. Nct; University of, L. The Effects of an 8-week Vegan Diet on TMAO Levels and Post-challenge Glucose Levels in Individuals With Dysglycaemia. **2017**. https://clinicaltrials.gov/study/NCT03134235
252. Nct; Azienda Ospedaliero-Universitaria, C. Meat-based Versus Pesco-vegetarian Diet and Colorectal Cancer. **2018**. https://clinicaltrials.gov/study/NCT03416777
253. Nct. Evaluation of the Plant-based Approaches to Stop Obesity Diet for the Treatment of Overweight and Obesity. https://clinicaltrials.gov/show/NCT03608176 **2018**.
254. Nct; Vascular Institute of, T. Effects of Plant-Based Diet on Peripheral Arterial Disease. **2019**. https://clinicaltrials.gov/study/NCT03798938
255. Nct; Charite University, B.G. Plant-based Nutrition for Patients With Cardiovascular Risk Factors. **2019**. https://clinicaltrials.gov/study/NCT03901183
256. Nct; Purdue University, Y. Effects of Replacing Starchy Vegetables and Refined Grains With Beef in a Vegetarian Diet on Cardio-metabolic Disease Risk Factors. **2019**. https://clinicaltrials.gov/study/NCT03925142
257. Nct; Azienda Ospedaliero-Universitaria, C. Gut Microbiota and Behcet's Syndrome: a Dietary Intervention Trial (MAMBA Study). **2019**. https://clinicaltrials.gov/study/NCT03962335
258. Nct; Edith Cowan, U. Vegetarian Diet in Ulcerative Colitis. **2019**. https://clinicaltrials.gov/study/NCT04018040
259. Nct; University of, R. Effects of a Plant-Based Diet on Insulin Requirements and Obesity Markers in Obese Adults With T2DM. **2019**. https://clinicaltrials.gov/study/NCT04048642
260. Nct; Physicians Committee for Responsible, M. Feasibility & Implementation of a Plant-Based Weight-Loss Program in an Office-Based Setting. **2019**. https://clinicaltrials.gov/study/NCT04091516
261. Nct; Antonio Di, M. GLP-1 and Oxyntomodulin Release in Relation to Diet in Type 2 Diabetes Patients. **2019**. https://clinicaltrials.gov/study/NCT04105608
262. Nct; University of, R. Effects of Whole Food, Plant-Based Nutrition on Chronic Kidney Disease With Proteinuria. **2019**. https://clinicaltrials.gov/study/NCT04171778
263. Nct; Per Bendix, J. The Function of Nutrition Rich Vegan Drink on Inflammatory Bowel Disease. **2019**. https://www.clinicaltrials.gov/study/NCT04297852
264. Nct; Griffin, H. The Impact of Consumption of Eggs in the Context of Plant-Based Diets on Endothelial Function, Diet Quality, and Cardio-Metabolic Risk Factors in Adults at Risk for Type 2 Diabetes. **2020**. https://clinicaltrials.gov/study/NCT04316429

265. Nct; University of, P. The Link Between Diets and Health Indicators. **2020**. https://clinicaltrials.gov/study/NCT04347213
266. Nct; Montefiore Medical, C. The Impact of Dietary Pattern on Erectile Function. **2020**. https://www.clinicaltrials.gov/study/NCT04349059
267. Nct; Fu Jen Catholic University, Y. Vegan Diet, Amla Fruits and Uric Acid. **2021**. https://clinicaltrials.gov/study/NCT04801745
268. Nct; Purdue University, Y. Effects of Proportioning Meat and Plant-based Protein-rich Foods on Cardiovascular Disease Risk Factors (S58). **2021**. https://clinicaltrials.gov/study/NCT04820829
269. Nct; Yes, K.T.O.K.U. Therapeutic Exercise and Vegan Diet on Pain and Quality of Life. **2021**. https://clinicaltrials.gov/study/NCT04923022
270. Nct; David Grant, U.S.A.F.M.C.Y. Facilitated Vegan Diet on Cardiometabolic Endpoints and Trimethylamine N-oxide. **2021**. https://clinicaltrials.gov/study/NCT05071196
271. Nct; Koç University, N. Metabolic Effects of Plant-based Diet. **2021**. https://clinicaltrials.gov/study/NCT05351853
272. Nct; State University of New York at Buffalo, N. A Pilot, Feasibility Study of Intermittent Caloric Restriction Plus Plant-based Diet in Cancer Patients Receiving Chemotherapy. **2022**. https://clinicaltrials.gov/study/NCT05359848
273. Nct; Optimal Health Research, Y. Diet Effect on Cancer Treatment Outcome. **2022**. https://clinicaltrials.gov/study/NCT05410002
274. Nct; Wageningen University, Y.; Rijksoverheid; Maastricht, U. Vegan Diets: the Short-term Effects on Daily Muscle Protein Synthesis Rates as Compared to Omnivorous Diets in Older Adults Assessed by D2O. **2022**. https://clinicaltrials.gov/study/NCT05624333
275. Nct; University of Jena, Y. Improvement of the Selenium Supply in a Vegan Diet by Different Selenium Sources. **2023**. https://clinicaltrials.gov/study/NCT05814874
276. Nct; Indiana University, Y. The Impact of Beef on Muscle Fatigue in Older Adults. **2023**. https://clinicaltrials.gov/study/NCT05860088
277. Nct; Westlake University, Y.; Hangzhou Third People's, H. The PRECISION-T2D Study: Precision Nutrition Study for Type 2 Diabetes. **2023**. https://clinicaltrials.gov/study/NCT05885828
278. Nct; University of Exeter, N. Vegan and Omnivorous Diets and Skeletal Muscle Turnover in Healthy Older Adults. **2023**. https://clinicaltrials.gov/study/NCT05985369
279. Nct; Amsterdam University of Applied Sciences, Y. Improvements by a Protein-Rich Plant-Based Diet and Resistance Exercise Program for Active Ageing. **2023**. https://clinicaltrials.gov/study/NCT06172725
280. Neuenschwander, M.; Hoffmann, G.; Schwingshackl, L.; Schlesinger, S. Impact of different dietary approaches on blood lipid control in patients with type 2 diabetes mellitus: a systematic review and network meta-analysis. *European Journal of Epidemiology* **2019**, *34*, 837-852, doi:https://dx.doi.org/10.1007/s10654-019-00534-1.
281. Neumann, C.G.; Jiang, L.; Weiss, R.E.; Grillenberger, M.; Gewa, C.A.; Siekmann, J.H.; Murphy, S.P.; Bwibo, N.O. Meat supplementation increases arm muscle area in Kenyan schoolchildren. *British Journal of Nutrition* **2013**, *109*, 1230-1240, doi:https://dx.doi.org/10.1017/S0007114512003121.
282. Nicholson, A.S.; Sklar, M.; Barnard, N.D.; Gore, S.; Sullivan, R.; Browning, S. Toward improved management of NIDDM: A randomized, controlled, pilot intervention using a lowfat, vegetarian diet. *Preventive Medicine* **1999**, *29*, 87-91.
283. Njike, V.Y.; Kela, G.C.M.; Treu, J.A.; Ayettey, R.G.; Kussaga, F.M.; Khan, N.; Comerford, B.; Agboola, O. Egg Consumption in the Context of Plant-Based Diets and Diet Quality in Adults at Risk for Type 2 Diabetes: A Randomized Single Blind Cross-over Controlled Trial. *Journal of the American Nutrition Association* **2023**, *42*, 130-139, doi:10.1080/07315724.2021.2006824.
284. Nl; Reade, D.-N.S.D.J.v.B.S.S.V. Plants for Joints RA. **2019**. http://www.who.int/trialsearch/Trial2.aspx?TrialID=NL7800
285. Nl; Reade, D.-N.S.D.J.v.B.S.S.V. Plants for Joints OA. **2019**. http://www.who.int/trialsearch/Trial2.aspx?TrialID=NL7801
286. Nl. Plants for Joints RA. http://www.who.int/trialsearch/Trial2.aspx?TrialID=NL7800 **2019**.
287. Nl. Plants for Joints OA. http://www.who.int/trialsearch/Trial2.aspx?TrialID=NL7801 **2019**.
288. Ochsner Health, S.; Fresenius Medical Care North, A.; DaVita, D.; Food Care, I. A Plant Based High Protein Diet to Improve Nutritional Outcomes in Peritoneal Dialysis Patients. **2015**. https://ClinicalTrials.gov/show/NCT02360748

289. Ohio State University Comprehensive Cancer, C. Nutrition and Exercise Trial: Improving Diet and Physical Activity Patterns in Overweight Cancer Survivors. **2017**. https://ClinicalTrials.gov/show/NCT03489213
290. Ohio, U. Retrospective Evaluation of Athens Complete Health Improvement Program (CHIP) Database. **2011**. https://ClinicalTrials.gov/show/NCT03025451
291. Olsen, T.; Stolt, E.; Øvrebø, B.; Elshorbagy, A.; Tore, E.C.; Lee-Ødegård, S.; Troensegaard, H.; Johannessen, H.; Doeland, B.; Vo, A.A.D.; et al. Dietary sulfur amino acid restriction in humans with overweight and obesity: a translational randomized controlled trial. **2023**, doi:10.1101/2023.09.09.23295155.
292. Oregon Research, I.; National Heart, L.; Blood, I. Lifestyle Management for Women With CHD and NIDDM. **1993**. https://ClinicalTrials.gov/show/NCT00005441
293. Ornish, D.; Brown, S.E.; Scherwitz, L.W.; Billings, J.H.; Armstrong, W.T.; Ports, T.A.; McLanahan, S.M.; Kirkeeide, R.L.; Brand, R.J.; Gould, K.L. Can lifestyle changes reverse coronary heart disease? The Lifestyle Heart Trial. *Lancet* **1990**, *336*, 129-133.
294. Ornish, D.M.; Lee, K.L.; Fair, W.R.; Pettengill, E.B.; Carroll, P.R. Dietary trial in prostate cancer: Early experience and implications for clinical trial design. *Urology* **2001**, *57*, 200-201.
295. Pagliai, G.; Dinu, M.; Cesari, F.; Gori, A.M.; Giusti, B.; Marcucci, R.; Casini, A.; Sofi, F. Randomized controlled dietary intervention trial comparing mediterranean and vegetarian diets for cardiovascular prevention: Preliminary results. *European Heart Journal* **2016**, *37*, 340, doi:10.1093/eurheartj/ehw432.
296. Pagliai, G.; Russo, E.; Niccolai, E.; Dinu, M.; Di Pilato, V.; Magrini, A.; Bartolucci, G.; Baldi, S.; Menicatti, M.; Giusti, B.; et al. Influence of a 3-month low-calorie Mediterranean diet compared to the vegetarian diet on human gut microbiota and SCFA: the CARDIVEG Study. *European Journal of Nutrition* **2019**, doi:10.1007/s00394-019-02050-0.
297. Paivarinta, E.; Itkonen, S.T.; Pellinen, T.; Lehtovirta, M.; Erkkola, M.; Pajari, A.M. Replacing Animal-Based Proteins with Plant-Based Proteins Changes the Composition of a Whole Nordic Diet-A Randomised Clinical Trial in Healthy Finnish Adults. *Nutrients* **2020**, *12*, 28, doi:https://dx.doi.org/10.3390/nu12040943.
298. Park, J.E.; Miller, M.; Rhyne, J.; Wang, Z.; Hazen, S.L. Differential effect of short-term popular diets on TMAO and other cardio-metabolic risk markers. *Nutrition Metabolism & Cardiovascular Diseases* **2019**, *29*, 513-517, doi:https://dx.doi.org/10.1016/j.numecd.2019.02.003.
299. Pasanisi, P.; Bruno, E.; Venturelli, E.; Morelli, D.; Peissel, B.; Manoukian, S. A randomized controlled lifestyle intervention in BRCA mutation carriers. *European journal of cancer* **2017**, *72*, S11, 162
300. Pham, K.; Joseph, S.K.Y.; Hill, R.; Haynes, A.; Pak, S.; Shah, S.A. Impact of Vegan Meal Kits on Cardiometabolic Endpoints: a Randomized, Controlled Clinical Trial. *Circulation* **2022**, *146*, doi:10.1161/circ.146.suppl_1.15086.
301. Phoenix, V.A.H.C.S.; Rouxbe Cooking, S.; Carl, T.H.M.R.F. Pilot Study on the Effects of a Plant-Strong Diet on Cardiovascular Risk Factors. **2015**. https://ClinicalTrials.gov/show/NCT03330548
302. Physicians Committee for Responsible, M. Womens Study to Alleviate Vasomotor Symptoms. **2020**. https://ClinicalTrials.gov/show/NCT04587154
303. Physicians Committee for Responsible, M. Feasibility & Implementation of a Plant-Based Weight-Loss Program in an Office-Based Setting. **2019**. https://ClinicalTrials.gov/show/NCT04091516
304. Physicians Committee for Responsible, M.; George Washington, U. Plant-Based Dietary Intervention in Type 2 Diabetes-2. **2014**. https://ClinicalTrials.gov/show/NCT01931631
305. Physicians Committee for Responsible, M.; National Institute of, D.; Digestive; Kidney, D.; Diabetes Action, R.; Education, F.; George Washington, U.; University of, T. Plant-Based Dietary Intervention in Type 2 Diabetes. **2003**. https://ClinicalTrials.gov/show/NCT00276939
306. Physicians Committee for Responsible, M.; Yale, U. Effect of a Dietary Intervention on Intracellular Lipid, Insulin Sensitivity, and Glycemic Control in Type 2 Diabetes. **2021**. https://ClinicalTrials.gov/show/NCT04088981
307. Piacquadio, K.; Gwin, J.; Leidy, H. Effect of Including Lean Red Meat in a Plant-Based Dietary Pattern on Biomarkers of Cardiometabolic Disease Risk. *Current developments in nutrition* **2023**, *7*, doi:10.1016/j.cdnut.2023.100790.
308. Pierce, J.P.; Faerber, S.; Wright, F.A.; Rock, C.L.; Newman, V.; Flatt, S.W.; Kealey, S.; Jones, V.E.; Caan, B.J.; Gold, E.B.; et al. A randomized trial of the effect of a plant-based dietary pattern on additional breast cancer events and survival: The Women's Healthy Eating and Living (WHEL) Study. *Controlled Clinical Trials* **2002**, *23*, 728-756, doi:10.1016/S0197-2456(02)00241-6.
309. Pirke, K.M.; Schweiger, U.; Laessle, R. Dieting influences the menstrual cycle vegetarian versus nonvegetarian diet. *Fertility and sterility* **1986**, *46*, 1083-1088.

310. Poikunnel Chacko, J.; Shetty, P.; Shetty, G.B. Evaluation of the physiological and psychological impact of easter lent fasting in obese individuals: emphasis on anthropometry, lipid profiles, liver functions, and mood. *International Journal Of Community Medicine And Public Health* **2025**, *12*, 2140-2146, doi:10.18203/2394-6040.ijcmph20251367.
311. Purdue, U. Dietary Pork, Appetite and Weight Loss in Human. https://ClinicalTrials.gov/show/NCT01006343
312. Rauma, A.L.; Nenonen, M.; Helve, T.; Hanninen, O. Effect of a strict vegan diet on energy and nutrient intakes by Finnish rheumatoid patients. *European Journal of Clinical Nutrition* **1993**, *47*, 747-749.
313. Richter, E.A.; Kiens, B.; Raben, A.; Tvede, N.; Pedersen, B.K. Immune parameters in male athletes after a lacto-ovo vegetarian diet and a mixed western diet. *Medicine and Science in Sports and Exercise* **1991**, *23*, 517-521.
314. Rinott, E.; Meir, A.Y.; Tsaban, G.; Zelicha, H.; Kaplan, A.; Knights, D.; Tuohy, K.; Scholz, M.U.; Koren, O.; Stampfer, M.J.; et al. The effects of the Green-Mediterranean diet on cardiometabolic health are linked to gut microbiome modifications: a randomized controlled trial. *Genome Medicine* **2022**, *14*, 29, doi:10.1186/s13073-022-01015-z.
315. Rock, C.L.; Flatt, S.W.; Wright, F.A.; Faerber, S.; Newman, V.; Kealey, S.; Pierce, J.P. Responsiveness of carotenoids to a high vegetable diet intervention designed to prevent breast cancer recurrence. *Cancer Epidemiology, Biomarkers & Prevention* **1997**, *6*, 617-623.
316. Rogerson, D.; Maçãs, D.; Milner, M.; Liu, Y.; Klonizakis, M. Contrasting effects of short-term mediterranean and vegan diets on microvascular function and cholesterol in younger adults: A comparative pilot study. *Nutrients* **2018**, *10*, doi:10.3390/nu10121897.
317. Sabino, J.; Vieira-Silva, S.; Machiels, K.; Joossens, M.; Falony, G.; Ferrante, M.; Van Assche, G.A.; Van Der Merwe, S.; Matthys, C.; Raes, J.; et al. Therapeutic manipulation of the gut microbiota through diet to reduce intestinal inflammation: Results from the fit trial. *Gastroenterology* **2017**, *152*, S1.
318. Sacks, F.M.; Katan, M. Randomized clinical trials on the effects of dietary fat and carbohydrate on plasma lipoproteins and cardiovascular disease. *American Journal of Medicine* **2002**, *113 Suppl 9B*, 13S-24S.
319. Sacks, F.M.; Wood, P.G.; Kass, E.H. Stability of blood pressure in vegetarians receiving dietary protein supplements. *Hypertension* **1984**, *6*, 199-201.
320. Sandkühler, J.F.; Kersting, X.; Faust, A.; Königs, E.K.; Altman, G.; Ettinger, U.; Lux, S.; Philipsen, A.; Müller, H.; Brauner, J. The effects of creatine supplementation on cognitive performance—a randomised controlled study. *BMC Medicine* **2023**, *21*, doi:10.1186/s12916-023-03146-5.
321. Sathiaraj, E.; Afshan, K.; R, S.; Jadoni, A.; Murugan, K.; Patil, S.; Naik, R. Effects of a Plant-Based High-Protein Diet on Fatigue in Breast Cancer Patients Undergoing Adjuvant Chemotherapy - a Randomized Controlled Trial. *Nutrition & Cancer* **2023**, *75*, 846-856, doi:10.1080/01635581.2022.2159044.
322. Scherwitz, L.W.; Brusis, O.A.; Kesten, D.; Safian, P.A.; Hasper, E.; Berg, A.; Siegrist, J. [Life style changes in patients with myocardial infarct in the framework of intramural and ambulatory rehabilitation--results of a German pilot study]. *Zeitschrift fur Kardiologie* **1995**, *84*, 216-221.
323. Schieren, A.; Huber, H.; Mantri, A.; Seel, W.; Stoffel-Wagner, B.; Coenen, M.; Nöthen, M.; Schmid, M.; Weinhold, L.; Krawitz, P.; et al. Effects Of Dietary Intervention On Plasma Lipid Profile Is Linked To Changes In The Microbiome Composition. *Clinical nutrition ESPEN* **2023**, *54*, 499, doi:10.1016/j.clnesp.2022.09.125.
324. Sciarrillo, C.; Guo, J.; Hengist, A.; Darcey, V.; Hall, K. Diet Order Affects Energy Intake and Weight Change During a Crossover Low-carbohydrate vs. Low-fat Diet Study. *Current developments in nutrition* **2023**, *7*, doi:10.1016/j.cdnut.2023.100754.
325. Sela, I.; Meir, A.Y.; Brandis, A.; Krajmalnik-Brown, R.; Zeibich, L.; Chang, D.; Dirks, B.; Tsaban, G.; Kaplan, A.; Rinott, E.; et al. Wolffia globosa–mankai plant-based protein contains bioactive vitamin b12 and is well absorbed in humans. *Nutrients* **2020**, *12*, 1-17, doi:10.3390/nu12103067.
326. Shah, B.; Ganguzza, L.; Slater, J.; Newman, J.D.; Allen, N.; Fisher, E.; Larigakis, J.; Ujueta, F.; Gianos, E.; Guo, Y.; et al. The effect of a vegan versus AHA DiEt in coronary artery disease (EVADE CAD) trial: study design and rationale. *Contemporary clinical trials communications* **2017**, *8*, 90-98, doi:10.1016/j.conctc.2017.09.003.
327. Shah, U.A.; Castro, F.; Anuraj, A.; Schach, E.; Derkach, A.; Joseph, N.S.; Adintori, P.A.; Guttentag, L.; Blaslov, J.; Cross, J.R.; et al. A Randomized Placebo Controlled Study of a Plant-Based Dietary Versus Supplement Versus Placebo Intervention in Patients with Monoclonal Gammopathy of Undetermined Significance (MGUS) and Smoldering Multiple Myeloma (SMM) - the Nutrition Prevention (NUTRIVENTION-3) Study. *Blood* **2022**, *140*, 5052-5055, doi:10.1182/blood-2022-159794.

328. Shin, P.K.; Park, S.J.; Kim, M.S.; Kwon, D.Y.; Kim, M.J.; Kim, K.C.; Chun, S.; Lee, H.J.; Choi, S.W. A traditional korean diet with a low dietary inflammatory index increases anti-inflammatory il-10 and decreases pro-inflammatory nf-κb in a small dietary intervention study. *Nutrients* **2020**, *12*, 1-11, doi:10.3390/nu12082468.
329. Skoldstam, L.; Brudin, L.; Hagfors, L.; Johansson, G. Weight reduction is not a major reason for improvement in rheumatoid arthritis from lacto-vegetarian, vegan or Mediterranean diets. *Nutrition Journal* **2005**, *4*, 15, 1-6
330. Slavícek, J.; Kittnar, O.; Dohnalová, A.; Trojan, S.; Novák, V.; Tichý, J.A.; Trefný, Z.M. Effect of a 10-day animal fat-free diet on cholesterol and glucose serum levels, blood pressure and body weight in 50-year-old volunteers. *Sborník lékarský* **2001**, *102*, 519-525.
331. Slavíček, J.; Kittnar, O.; Fraser, G.E.; Medová, E.; Konečná, J.; Žižka, R.; Dohnalová, A.; Novák, V. Lifestyle decreases risk factors for cardiovascular diseases. *Central European Journal of Public Health* **2008**, *16*, 161-164.
332. Slavíček, J.; Kittnar, O.; Medová, E.; Konečná, J.; Žižka, R. Lifestyle modification helps to reduce the risk factors of cardiovascular disease. *Cor et Vasa* **2007**, *49*, 88-91.
333. Soroka, N.; Silverberg, D.S.; Greemland, M.; Birk, Y.; Blum, M.; Peer, G.; Iaina, A. Comparison of a vegetable-based (soya) and an animal-based low-protein diet in predialysis chronic penal failure patients. *Nephron* **1998**, *79*, 173-180, doi:10.1159/000045021.
334. Spiller, G.A.; Miller, A.; Olivera, K.; Reynolds, J.; Miller, B.; Morse, S.J.; Dewell, A.; Farquhar, J.W. Effects of plant-based diets high in raw or roasted almonds, or roasted almond butter on serum lipoproteins in humans. *Journal of the American College of Nutrition* **2003**, *22*, 195-200.
335. St. Michael's Hospital, T.; University of, T.; Laval, U.; University of, M.; Canola Council of, C. Canola-Mediterranean Diet Study in T2DM. **2014**. https://ClinicalTrials.gov/show/NCT03718988
336. Stanford, U. SWAP-MEAT: Study With Appetizing Plant Food - Meat Eating Alternatives Trial. **2019**. https://ClinicalTrials.gov/show/NCT02245399
337. Swapnali; Kisan, R. A study of dietary habits and early renal damage in diabetes mellitus patients. *Indian Journal of Clinical Biochemistry* **2016**, *31*, S111, doi:10.1007/s12291-016-0628-z.
338. Tang, M.; Matz, K.L.; Berman, L.M.; Davis, K.N.; Melanson, E.L.; Frank, D.N.; Hendricks, A.E.; Krebs, N.F. Effects of Complementary Feeding With Different Protein-Rich Foods on Infant Growth and Gut Health: Study Protocol. *Frontiers in Pediatrics* **2021**, *9*, 793215, doi:https://dx.doi.org/10.3389/fped.2021.793215.
339. Tctr; Rarchadapisek Research Funds, N. Effect of Whole Food Plant-based Meal for Dinner on BMI among Overweight & Obese Patients. **2022**. https://www.thaiclinicaltrials.org/show/TCTR20220119008
340. Tel-Aviv Sourasky Medical, C. Mediterranean Diet, Circuit Resistance Training, Empagliflozin in Elderly With Type 2 Diabetes: a Study Protocol. **2018**. https://ClinicalTrials.gov/show/NCT03560375
341. Tel-Aviv Sourasky Medical, C.; KaMa, H.T.C.f.H.-P.T. Effect of Vegan Diet and Lifestyle Changes on Indolent Lymphoma During Controlled Waiting Period. **2021**. https://ClinicalTrials.gov/show/NCT04957693
342. The Cleveland, C. Plant-Based, American Heart Assoc. or Mediterranean Diets In 9-18 yo With BMI >95%, Cholesterol >169 and Their Parents. **2016**. https://ClinicalTrials.gov/show/NCT02857543
343. The Cleveland, C. Low Fat Vegan or American Heart Association Diets & Cardiovascular Risk in Obese 9-18 y.o. With Elevated Cholesterol. **2013**. https://ClinicalTrials.gov/show/NCT01817491
344. Thomas, M.S.; Huang, L.; Garcia, C.; Sakaki, J.R.; Blesso, C.N.; Chun, O.K.; Fernandez, M.L. The Effects of Eggs in a Plant-Based Diet on Oxidative Stress and Inflammation in Metabolic Syndrome. *Nutrients* **2022**, *14*, doi:10.3390/nu14122548.
345. Thomson, C.A.; Rock, C.L.; Giuliano, A.R.; Newton, T.R.; Cui, H.; Reid, P.M.; Green, T.L.; Alberts, D.S. Longitudinal changes in body weight and body composition among women previously treated for breast cancer consuming a high-vegetable, fruit and fiber, low-fat diet. *European Journal of Nutrition* **2005**, *44*, 18-25, doi:10.1007/s00394-004-0487-x.
346. Tsaban, G.; Yaskolka Meir, A.; Rinott, E.; Zelicha, H.; Kaplan, A.; Shalev, A.; Katz, A.; Rudich, A.; Tirosh, A.; Shelef, I.; et al. The effect of green Mediterranean diet on cardiometabolic risk; a randomised controlled trial. *Heart* **2020**, *23*, 23, doi:https://dx.doi.org/10.1136/heartjnl-2020-317802.
347. Turner-McGrievy, B.; Liese, A.; Wilcox, S.; Friedman, D.; Sarzynski, M.; Bailey, S.; Carswell, J.; Wilson, M. The DG3D Study: 12-Week Randomized Weight Loss and Diet Quality Intervention Among African Americans. *Obesity (Silver Spring, Md.)* **2022**, *30*, 37, doi:10.1002/oby.23625.
348. Turner-McGrievy, G.M.; Barnard, N.D.; Cohen, J.; Jenkins, D.J.; Gloede, L.; Green, A.A. Changes in nutrient intake and dietary quality among participants with type 2 diabetes following a low-fat vegan diet

or a conventional diabetes diet for 22 weeks. *Journal of the American Dietetic Association* **2008**, *108*, 1636-1645, doi:https://dx.doi.org/10.1016/j.jada.2008.07.015.
349. Turner-McGrievy, G.M.; Davidson, C.R.; Wilcox, S. Does the type of weight loss diet affect who participates in a behavioral weight loss intervention? A comparison of participants for a plant-based diet versus a standard diet trial. *Appetite* **2014**, *73*, 156-162, doi:https://dx.doi.org/10.1016/j.appet.2013.11.008.
350. Turner-McGrievy, G.M.; Davidson, C.R.; Wingard, E.E.; Billings, D.L. Low glycemic index vegan or low-calorie weight loss diets for women with polycystic ovary syndrome: a randomized controlled feasibility study. *Nutrition Research* **2014**, *34*, 552-558, doi:https://dx.doi.org/10.1016/j.nutres.2014.04.011.
351. Turner-McGrievy, G.M.; Davidson, C.R.; Wingard, E.E.; Wilcox, S.; Frongillo, E.A. Comparative effectiveness of plant-based diets for weight loss: a randomized controlled trial of five different diets. *Nutrition* **2015**, *31*, 350-358, doi:https://dx.doi.org/10.1016/j.nut.2014.09.002.
352. Turner-McGrievy, G.M.; Leach, A.M.; Wilcox, S.; Frongillo, E.A. Differences in Environmental Impact and Food Expenditures of Four Different Plant-based Diets and an Omnivorous Diet: Results of a Randomized, Controlled Intervention. *Journal of Hunger and Environmental Nutrition* **2016**, *11*, 382-395, doi:10.1080/19320248.2015.1066734.
353. Turner-McGrievy, G.M.; Wilcox, S.; Frongillo, E.A.; Murphy, E.A.; Hutto, B.; Wilson, M.; Davey, M.; Bernhart, J.A.; Okpara, N.; Bailey, S.; et al. Effect of a Plant-Based vs Omnivorous Soul Food Diet on Weight and Lipid Levels Among African American Adults: a Randomized Clinical Trial. *JAMA Network Open* **2023**, *6*, e2250626, doi:10.1001/jamanetworkopen.2022.50626.
354. Turner-McGrievy, G.M.; Wilson, M.J.; Carswell, J.; Okpara, N.; Aydin, H.; Bailey, S.; Davey, M.; Hutto, B.; Wilcox, S.; Friedman, D.B.; et al. A 12-Week Randomized Intervention Comparing the Healthy US, Mediterranean, and Vegetarian Dietary Patterns of the US Dietary Guidelines for Changes in Body Weight, Hemoglobin A1c, Blood Pressure, and Dietary Quality among African American Adults. *Journal of Nutrition* **2023**, *153*, 579-587, doi:10.1016/j.tjnut.2022.11.020.
355. Turner-McGrievy, G.M.; Wirth, M.D.; Shivappa, N.; Wingard, E.E.; Fayad, R.; Wilcox, S.; Frongillo, E.A.; Hebert, J.R. Randomization to plant-based dietary approaches leads to larger short-term improvements in Dietary Inflammatory Index scores and macronutrient intake compared with diets that contain meat. *Nutrition Research* **2015**, *35*, 97-106, doi:https://dx.doi.org/10.1016/j.nutres.2014.11.007.
356. Turner-McGrievy, G.; Wilcox, S.; Frongillo, E.A.; Murphy, A.; Hutto, B.; Williams, K.; Crimarco, A.; Wilson, M.; Davey, M. The Nutritious Eating with Soul (NEW Soul) Study: Study design and methods of a two-year randomized trial comparing culturally adapted soul food vegan vs. omnivorous diets among African American adults at risk for heart disease. *Contemporary Clinical Trials* **2020**, *88*, 105897, doi:https://dx.doi.org/10.1016/j.cct.2019.105897.
357. Oakland, U.B.C.s.H.; Dairy Management, I. The Mediterranean Full-Fat Dairy Study. **2016**. https://ClinicalTrials.gov/show/NCT02781675
358. Universität, D.-E.; Kliniken, E.-M.; Immanuel Hospital Berlin, B.G.; Charite University, B.G.; University of, W.H. Comprehensive Lifestyle Modification for Patients With Hypertension and Metabolic Syndrome: a Multicenter Randomized Controlled Trial. **2014**. https://ClinicalTrials.gov/show/NCT02099968
359. University of Alabama at, B.; United States Department of, D.; Loma Linda, U.; Brenda Davis Nutrition Consultation, S.; Canvasback Missions, I. Lifestyle Intervention to Treat Diabetes in the Marshall Islands. **2006**. https://ClinicalTrials.gov/show/NCT03862963
360. University of Campinas, B. Fasting Mimicking Diet and Beige/Brown Adipose Tissue in Humans. **2020**. https://ClinicalTrials.gov/show/NCT04385615
361. University of, R. Effects of a Plant-Based Diet on Insulin Requirements and Obesity Markers in Obese Adults With T2DM. **2020**. https://ClinicalTrials.gov/show/NCT04048642
362. University of South, C. The Dietary Guidelines 3 Diets Study. **2021**. https://ClinicalTrials.gov/show/NCT04981847
363. University of South, C.; Academy of, N.; Dietetics. The Plant-Based and Soul-Full Study (PASS). **2018**. https://ClinicalTrials.gov/show/NCT03565718
,364. University of, T.; Canada Research Chairs Endowment of the Federal Government of, C.; Natural, S.; Engineering Research Council, C.; Loblaw Companies, L.; Almond Board of, C.; Unilever, R. Effects of a Long Term Dietary Portfolio and of Sequential Removal of Food Components From the Diet. **2003**. https://ClinicalTrials.gov/show/NCT00438893
365. University of, T.; Canadian Institutes of Health, R.; Loblaw Companies, L.; Solae, L.L.C.; Unilever, R. Portfolio 5 - Multicentre Dietary Advice on Serum Lipids in Hyperlipidemia. **2007**. https://ClinicalTrials.gov/show/NCT00438425

366. Varaeva, Y.R.; Starodubova, A.V.; Kosyura, S.D.; Livantsova, E.N. Short-term effects of different dietary approaches on body weight and body composition among obese female. *Obesity Facts* **2018**, *11*, 135-136, doi:10.1159/000489691.
367. Veleba, J.; Klementova, M.; Belinova, L.; Haluzik, M.; Pavlovicova, R.; Hill, M.; Pelikanova, T.; Kahleova, H. A plant-based meal increases gastrointestinal hormones and satiety more than an energy-and macronutrient-matched standard meal in T2D, obese, and healthy participants: A three-group randomized crossover study. *Diabetes* **2019**, *68*, doi:10.2337/db19-1914-P.
368. Veleba, J.; Matoulek, M.; Hill, M.; Pelikanova, T.; Kahleova, H. "A Vegetarian vs. Conventional Hypocaloric Diet: The Effect on Physical Fitness in Response to Aerobic Exercise in Patients with Type 2 Diabetes." A Parallel Randomized Study. *Nutrients* **2016**, *8*, 26, doi: https://doi.org/10.3390/nu8110671
369. von Lossonczy, T.O.; Ruiter, A.; Bronsgeest-Schoute, H.C.; van Gent, C.M.; Hermus, R.J. The effect of a fish diet on serum lipids in healthy human subjects. *American Journal of Clinical Nutrition* **1978**, *31*, 1340-1346.
370. Walrabenstein, W.; Van Schaardenburg, D. A randomized controlled trial (RCT) of a multidisciplinary lifestyle program in patients with (increased risk for) rheumatoid arthritis and osteoarthritis: Design and inclusion rate. *Complementary Medicine Research* **2021**, *28*, 8, doi:10.1159/000514476.
371. Walrabenstein, W.; Wagenaar, C.A.; van de Put, M.; van der Leeden, M.; Gerritsen, M.; Twisk, J.W.R.; van der Esch, M.; van Middendorp, H.; Weijs, P.J.M.; Roorda, L.D.; et al. A multidisciplinary lifestyle program for metabolic syndrome-associated osteoarthritis: the "Plants for Joints" randomized controlled trial. *Osteoarthritis and cartilage* **2023**, doi:10.1016/j.joca.2023.05.014.
372. Walrabenstein, W.; Wagenaar, C.A.; van der Leeden, M.; Turkstra, F.; Twisk, J.W.R.; Boers, M.; van Middendorp, H.; Weijs, P.J.M.; van Schaardenburg, D. A multidisciplinary lifestyle program for rheumatoid arthritis: the 'Plants for Joints' randomized controlled trial. *Rheumatology* **2023**, *62*, 2683-2691, doi:https://dx.doi.org/10.1093/rheumatology/keac693.
373. Walrabenstein, W.; Wagenaar, C.; Van Der Leeden, M.; Turkstra, F.; Twisk, J.; Boers, M.; Van Middendorp, H.; Weijs, P.; Van Schaardenburg, D. Effect of a Multidisciplinary Lifestyle Program in Patients with Rheumatoid Arthritis. *Annals of the rheumatic diseases* **2022**, *81*, 1326-1327, doi:10.1136/annrheumdis-2022-eular.1689.
374. Wang, T.; Cassidy, S.; Kroeger, C.; Mitra, S.; Ribeiro, R.; Masedunskas, A.; Huang, R.; Fontana, L. Impact of an Intensive Lifestyle Program on Low Attenuation Plaque and Myocardial Perfusion in Coronary Heart Disease: a Randomised Clinical Trial Protocol. *Current developments in nutrition* **2023**, *7*, doi:10.1016/j.cdnut.2023.100188.
375. Warziski, M.T.; Sereika, S.M.; Styn, M.A.; Music, E.; Burke, L.E. Changes in self-efficacy and dietary adherence: the impact on weight loss in the PREFER study. *Journal of Behavioral Medicine* **2008**, *31*, 81-92.
376. Washington University School of, M.; Barnes-Jewish, H. Effects of a Calorie Restricted, Very Low Fat Plant-based Diet and Multi-component Exercise Program on Metabolic Health. **2016**. https://ClinicalTrials.gov/show/NCT02706288
377. Watts, G.F.; Ahmed, W.; Quiney, J.; Houlston, R.; Jackson, P.; Iles, C.; Lewis, B. Effective lipid lowering diets including lean meat. *British medical journal (Clinical research ed.)* **1988**, *296*, 235-237, doi:10.1136/bmj.296.6617.235.
378. Wright, N.; Wilson, L.; Smith, M.; Duncan, B.; McHugh, P. The BROAD study: A randomised controlled trial using a whole food plant-based diet in the community for obesity, ischaemic heart disease or diabetes. *Nutrition & Diabetes* **2017**, *7*, e256, doi:https://dx.doi.org/10.1038/nutd.2017.3.
379. Yadav, V.; Marracci, G.; Kim, E.; Spain, R.; Cameron, M.; Overs, S.; McDougall, J.; Lovera, J.; Bourdette, D. Effects of a low fat plant based diet in multiple sclerosis (MS): Results of a 1-year long randomized controlled (RC) study. *Neurology* **2014**, *82*, 10 Supplement, P6.152
380. Yadav, V.; Marracci, G.; Kim, E.; Spain, R.; Cameron, M.; Overs, S.; Riddehough, A.; Li, D.K.; McDougall, J.; Lovera, J.; et al. Low-fat, plant-based diet in multiple sclerosis: a randomized controlled trial. *Multiple Sclerosis and Related Disorders* **2016**, *9*, 80-90, doi:10.1016/j.msard.2016.07.001.
381. Yamashita, T.; Sasahara, T.; Pomeroy, S.E.; Collier, G.; Nestel, P.J. Arterial compliance, blood pressure, plasma leptin, and plasma lipids in women are improved with weight reduction equally with a meat-based diet and a plant-based diet. *Metabolism: Clinical and Experimental* **1998**, *47*, 1308-1314, doi:10.1016/S0026-0495(98)90297-9.
382. Yaskolka Meir, A.; Rinott, E.; Tsaban, G.; Zelicha, H.; Kaplan, A.; Rosen, P.; Shelef, I.; Youngster, I.; Shalev, A.; Blüher, M.; et al. Effect of green-Mediterranean diet on intrahepatic fat: the DIRECT PLUS randomised controlled trial. *Gut* **2021**, *70*, 2085-2095, doi:10.1136/gutjnl-2020-323106.

383. Zelicha, H.; Kloting, N.; Kaplan, A.; Yaskolka Meir, A.; Rinott, E.; Tsaban, G.; Chassidim, Y.; Bluher, M.; Ceglarek, U.; Isermann, B.; et al. The effect of high-polyphenol Mediterranean diet on visceral adiposity: the DIRECT PLUS randomized controlled trial. *BMC Medicine* **2022**, *20*, 327, doi:10.1186/s12916-022-02525-8.
384. Zhu, R.; Fogelholm, M.; Poppitt, S.D.; Silvestre, M.P.; Moller, G.; Huttunen-Lenz, M.; Stratton, G.; Sundvall, J.; Raman, L.; Jalo, E.; et al. Adherence to a Plant-Based Diet and Consumption of Specific Plant Foods-Associations with 3-Year Weight-Loss Maintenance and Cardiometabolic Risk Factors: A Secondary Analysis of the PREVIEW Intervention Study. *Nutrients* **2021**, *13*, 01, doi:https://dx.doi.org/10.3390/nu13113916.
385. Whole-Food Plant-Based Diet to Control Weight and MetaboInflammation in Overweight/Obese Men With Prostate Cancer. https://classic.clinicaltrials.gov/show/NCT05471414
386. Whole Food Plant-Based Diet for HIV-Associated Reduction in Cardiovascular Risk (PLANT-HART). https://classic.clinicaltrials.gov/show/NCT05796882
387. Weight Management for the Remission of Type 2 Diabetes Using a Proprietary Meal Replacement System-Diabetes Remission Study (DRS). https://classic.clinicaltrials.gov/show/NCT05397028
388. Weight Loss Intervention With Lean Muscle Mass Retention (WLMR) Study. **2022**. https://classic.clinicaltrials.gov/show/NCT05607628
389. Twins Nutrition Study (TwiNS): Vegan vs. Omnivore. https://classic.clinicaltrials.gov/show/NCT05297825
390. The Impact of Plant-Based Protein-rich Food Products With Varying Degree of Processing on the Human Gut Microbiome Composition and Human Metabolome. https://classic.clinicaltrials.gov/show/NCT05885750
391. The Effect of Vegetarian Diet on Patients With Metabolic Associated Fatty Liver Disease. https://classic.clinicaltrials.gov/show/NCT05443581
392. The Effect of an Online Plant-Based Dietary Program on Cardiovascular Risk Factors in Persons With Type 2 Diabetes Mellitus: A Randomized Controlled Trial. https://classic.clinicaltrials.gov/show/NCT05777746
393. The Effect of a 2-week Preoperative Vegan Diet Versus Omnivorous Diet on the Protein Turnover in the Osteoarthritic Knee. https://classic.clinicaltrials.gov/show/NCT06130956
394. The Effect of a 12-week Self-composed Vegan Diet With or Without Concurrent Resistance Exercise on Thigh Muscle Volume in Older Adults. https://classic.clinicaltrials.gov/show/NCT05809466
395. The Dietary Guidelines 3 Diet Patterns Study (DG3D): Phase 2. https://classic.clinicaltrials.gov/show/NCT05254496
396. The CARING Study: Creating and Restoring Health Through Nutrition Guidance. https://classic.clinicaltrials.gov/show/NCT05795439
397. Study of Nutrition in Postpartum and Early Life Feeding Study. https://classic.clinicaltrials.gov/show/NCT06082921
398. Singapore Healthy Alternative Protein Evaluation Study. https://classic.clinicaltrials.gov/show/NCT05446753
399. Role of Lean-pork Within a Plant-based Dietary Pattern. https://classic.clinicaltrials.gov/show/NCT05581953
400. Remotely Delivered Behavioral Weight Loss Using an Ad Libitum Plant-Based Diet Versus a Balanced Calorie Deficit Diet. https://classic.clinicaltrials.gov/show/NCT05337150
401. Protein Quantity and Quality in Older Subjects. https://classic.clinicaltrials.gov/show/NCT05301179
402. Plant-Focused Nutrition in Patients With Diabetes and Chronic Kidney Disease. https://classic.clinicaltrials.gov/show/NCT05514184
403. Nutritious Eating With Soul Dissemination and Implementation. https://classic.clinicaltrials.gov/show/NCT05659966
404. Nutritional Intervention for Endometriosis. https://classic.clinicaltrials.gov/show/NCT05175248
405. Multimodal Project. https://classic.clinicaltrials.gov/show/NCT05656716
406. Low-Carbohydrate and Plant-Based Dietary Effects on Vascular Health. https://classic.clinicaltrials.gov/show/NCT05414851
407. Implementation of a Mediterranean Diet Program for Overweight or Obese Pregnant Women in a Low-resource Clinical Setting. https://classic.clinicaltrials.gov/show/NCT05868954
408. Impact of a Mediterranean Diet on Cardiovascular Disease Risk Factors. https://classic.clinicaltrials.gov/show/NCT06113484
409. Growth Study Using Else Toddler Nutritional Drink vs. a. Dairy Based Toddler Drink (Control) in Healthy Toddlers. https://classic.clinicaltrials.gov/show/NCT05576870

410. Fasting-mimicking Diet in Treatment of Depressive Symptoms in IBD. https://classic.clinicaltrials.gov/show/NCT05382897
411. Fasting-mimicking Diet and Longevity Diet, Body Composition and Aging. https://classic.clinicaltrials.gov/show/NCT05698654
412. Effects of Pulses Through the Gut Microbiome and Bioavailability of Bioactive Compounds. https://classic.clinicaltrials.gov/show/NCT05999136
413. Effects of Lean Pork Loin Intake on Protein Homeostasis and Glucose Regulation in Prediabetic Adults.https://classic.clinicaltrials.gov/show/NCT06025292
414. Effects of Diet-Modulated Autologous Fecal Microbiota Transplantation on Weight Regain. *Gastroenterology* **2020**, doi:10.1053/j.gastro.2020.08.041.
415. Effects of Beef Consumption on Skeletal Muscle Protein Homeostasis and Inflammatory Factors in Pre- and Postmenopausal Females. https://classic.clinicaltrials.gov/show/NCT05714462
416. Effect of Tirzepatide Plus Intensive Lifestyle Therapy on Body Weight and Metabolic Health in Latinos With Obesity. https://classic.clinicaltrials.gov/show/NCT06009653
417. Effect of the Sustainable Diet on Gut Microbiota and the Metabolome: a Randomised Crossover Study. https://classic.clinicaltrials.gov/show/NCT05231317
418. Effect of Partial Dietary Replacement From Animal to Plant-Based Protein for Type 2 Diabetes Management. https://classic.clinicaltrials.gov/show/NCT05706155
419. Effect of Low-Calorie Diet and Lifestyle Intervention on Reversal of T2DM. https://classic.clinicaltrials.gov/show/NCT05925946
420. Effect of Intermittent Calorie Restriction on MASLD Patients With Abnormal Glucose Metabolism. ttps://classic.clinicaltrials.gov/show/NCT04283942
421. Effect of a Vegetarian Meal on the Physiology of Insulin Response in Patients With Type 2 Diabetes Mellitus and Obesity. https://classic.clinicaltrials.gov/show/NCT06152536
422. Effect of a Pulse-based USDA-diet on Healthspan. https://classic.clinicaltrials.gov/show/NCT05577858
423. Digestibility of Different Plant-based Proteins in Humans With Ileostomy. https://classic.clinicaltrials.gov/show/NCT06142084
424. Diets, Lipoproteins and Inflammation Markers. https://classic.clinicaltrials.gov/show/NCT05423457
425. Dietary Proteins: Metagenomic and Metabolomics Approaches for Human Biomarkers Identification. https://classic.clinicaltrials.gov/show/NCT05611138
426. Dietary Intervention to Improve Kidney Transplant Outcomes. Dietary Intervention to Improve Kidney Transplant Outcomes. https://classic.clinicaltrials.gov/show/NCT05449496
427. Behavioral Plant-Based Dietary Intervention in Latinos. https://classic.clinicaltrials.gov/show/NCT05444595
428. Athlete Whey Protein Sensitivity: Prevalence and Performance. https://classic.clinicaltrials.gov/show/NCT05482997
429. A Study of a Plant-Based Diet and Dietary Supplements in People With Smoldering Multiple Myeloma (SMM). https://classic.clinicaltrials.gov/show/NCT06055894
