# Supplementary material for: Health Effects of Plant-Based Diets in People with Overweight or Obesity: A Systematic Review and Meta-Analysis
Source: Nutrients. 2026 Jun 19;18(12):1987. doi: 10.3390/nu18121987 (PMC13304861; doi:10.3390/nu18121987)
Supplement: Supplementary file 1 [file nutrients-18-01987-s001.zip › Supplementary File S3_Charachteristics of included studies.pdf]

Supplementary file S3

Table S3. Characteristics of included studies

**Author, publication year**	**Barnard 2005** [1]
**Title**	The effects of a low-fat, plant-based dietary intervention on body weight, metabolism, and insulin sensitivity
**DOI**	10.1016/j.amjmed.2005.03.039
**Trial identifier (if registered)**	Not mentioned
**Methods**	**Study design:** parallel randomized controlled trial<br>**Unit of randomisation:** individual<br>**Blinding:** All physical and metabolic measurements were made by clinician's blind to group assignment.<br>**Number of study arms:** 2
**Participants**	**Country where trial was performed:** United States of America<br>**Location/Setting**: Washington<br>**Number of study centres:** 1<br><br>**Sample size (No. of participants entering the study):** n = 59 (29/30)<br>**Dropouts/withdrawals:** control group n = 2<br>**Sex**: female<br><br>**Inclusion criteria (as reported):** overweight or obese (body mass index [BMI] 26-44 kg/m^2) postmenopausal women<br><br>**Exclusion criteria (as reported):** possible hormonal effects on metabolic measures, unstable medical status, history of eating disorder or substance abuse, severe mental illness, previously diagnosed diabetes, physical conditions affecting body weight (e.g., Cushing's disease), recent use of oestrogens, medications affecting appetite or body weight, or tobacco use<br><br>**Characteristics of participants (e.g. occupation, health status):** postmenopausal women<br>**Age of participants:** mean age 56,5
**Interventions/**	**Intervention:** low-fat vegan diet<br>**Comparator:** National Cholesterol Education Program Step II (NCEP) diet<br><br>**Duration of intervention:** 14 weeks<br>**Duration of follow-up:** -<br>**Run-in period:** -
**Outcomes**	**Reported outcomes in full text of publication:**<br>weight, BMI, body fat (%), lean mass (kg), waist (cm, in), hip (cm, in), waist-hip ratio, resting metabolic rate, thermic effect of food, fasting glucose, mean glucose, fasting insulin, mean insulin, insulin sensitivity, change in energy intake, change in resting metabolism, change in thermic effect of food, change in energy expenditure<br>**Timing of outcome assessment:** not applicable
**Publication details**	**Language of publication:** English<br>**Funding:** -

	**Conflict of interest** (quote)**:** not mentioned<br>**Publication status:** full article in peer-reviewed journal
**Stated aim for study**	**Quote:** "We therefore conducted a randomized, controlled trial to quantify the short-term effect of a low-fat, vegan diet on body weight, body composition, metabolism, and insulin sensitivity in overweight, postmenopausal women, using for comparison a more moderate low-fat diet."
**Note**	**Study start date:** not mentioned<br>**Study end date:** not mentioned

**Author, publication year**	**Barnard 2021** [2]
**Title**	A Mediterranean Diet and Low-Fat Vegan Diet to Improve Body Weight and Cardiometabolic Risk Factors: A Randomized, Cross-over Trial
**DOI**	10.1080/07315724.2020.1869625
**Trial identifier (if registered)**	NCT03698955
**Methods**	**Study design:** randomized crossover trial<br>**Unit of randomisation:** individual<br>**Blinding:** The statistician was blinded to the hypothesized effects of interventions and group assignment.<br>**Number of study arms:** 2
**Participants**	**Country where trial was performed:** United States of America<br>**Location/Setting**: Washington, DC<br>**Number of study centres:** 1<br><br>**Sample size (No. of participants entering the study):** n = 62 (30/32)<br>**Dropouts/withdrawals:** 4 participants dropped out during the vegan phase; 6 participants dropped out during the Mediterranean phase<br>**Sex**: both<br><br>**Inclusion criteria (as reported):** adults with BMI 28-40 kg/m^2<br><br>**Exclusion criteria (as reported): T**ype 1 diabetes, smoking, alcohol or drug abuse, pregnancy or lactation, and current use of a vegan or mediterranean diet<br>**Characteristics of participants (e.g. occupation, health status):** healthy individuals<br>**Age of participants:** mean age 57.4
**Interventions/**	**Intervention:** low fat vegan diet<br>**Comparator:** mediterranean diet<br><br>**Duration of intervention:** 36 weeks (2 periods)<br>**Duration of follow-up:** -<br>**Run-in period:** -
**Outcomes**	**Reported outcomes in full text of publication:**<br>Dietary intake physical activity, anthropometric variables and body composition, parameters of glucose control and insulin resistance, blood lipids, blood pressure<br>**Timing of outcome assessment:** not applicable
**Publication details**	**Language of publication:** English

	**Funding:** "This work was funded by the Physicians Committee for Responsible Medicine."<br>**Conflict of interest** (quote)**:** not mentioned<br>**Publication status:** full article in peer-reviewed journal
**Stated aim for study**	**Quote:** "The present study directly compared a Mediterranean and a vegan diet for their effects on weight and cardiometabolic parameters, using a cross-over design. Based on the findings of prior studies, it tested the hypothesis that, compared with a Mediterranean diet, a low-fat vegan diet results in greater changes in body weight, total and LDL-cholesterol concentrations, and insulin sensitivity."
**Note**	**Study start date:** February 2019<br>**Study end date:** October 2019

**Author, publication year**	**Jenkins 2014** [3]
**Title**	Effect of a 6-month vegan low-carbohydrate ('Eco-Atkins') diet on cardiovascular risk factors and body weight in hyperlipidaemic adults: a randomised controlled trial
**DOI**	doi:10.1136/bmjopen-2013-003505
**Trial identifier (if registered)**	NCT00256516
**Methods**	**Study design:** parallel randomized controlled trial<br>**Unit of randomisation:** individual<br>**Blinding:** "Neither the dietitians nor participants could be blinded, but equal emphasis was placed on the potential importance for health of both diets. The analytical technicians were blinded to diet allocation, as was the statistician, up to analysis of the primary outcome."<br>**Number of study arms:** 2
**Participants**	**Country where trial was performed:** Canada<br>**Location/Setting**: Canada<br>**Number of study centres:** 1<br>**Sample size (No. of participants entering the study):** n = 50 (22/22)<br>**Dropouts/withdrawals:** n = 3<br>**Sex**: both<br><br>**Inclusion criteria (as reported):** healthy men and postmenopausal women between the ages of 21 and 70 years, with a high-normal or raised LDL-C concentration (>131 mg/dL [to convert to millimoles per litre, multiply by 0.0259] at diagnosis), TG concentration higher than 44 mg/dL (to convert to millimoles per litre, multiply by 0.0113) but lower than 442 mg/dL, a body mass index (BMI) (calculated as weight in kilograms divided by height in meters squared) higher than 27 and who were not currently involved in a weight-loss program<br><br>**Exclusion criteria (as reported):** lipid-lowering medications, hormone therapy, alcohol consumption of more than 2 drinks/d, tobacco use, major cardiovascular event or surgery in the preceding 6 months, diabetes, untreated hypothyroidism, blood pressure (BP) higher than 140/90 mm Hg, renal or liver disease, cancer (excluding nonmelanoma skin cancer), or any food allergies<br><br>**Characteristics of participants (e.g. occupation, health status):** healthy men and postmenopausal women, BMI > 27 kg/m^2

	**Age of participants:** mean age 57
**Interventions/**	**Intervention:** low-carbohydrate plant-based diet<br>**Comparator:** high-carbohydrate lacto-ovo vegetarian diet<br><br>**Duration of intervention:** 1 month (0 week, 2 week, 4 week)<br>**Duration of follow-up:** 6 months (ad libitum phase)<br>**Run-in period:** -
**Outcomes**	**Reported outcomes in full text of publication:**<br>weight, BMI, blood pressure, cholesterol, triglycerides, glucose, exercise, medications, HOMA-IR, satiety, apolipoproteins<br>**Timing of outcome assessment:** not applicable
**Publication details**	**Language of publication:** English<br>**Funding:** "This study was supported by Solae, LLC, Loblaw Companies Limited, and the Canadian Research Chair Program of the Federal Government of Canada."<br>**Conflict of interest** (quote)**:** not mentioned<br>**Publication status:** full article in peer-reviewed journal
**Stated aim for study**	**Quote: "**In view of the apparent success of low-carbohydrate diets for weight loss and the demonstration that relatively high-carbohydrate diets low in animal products lower CHD risk factors,3,9-11 we determined the effect of a low-carbohydrate weight-loss diet, without the use of animal products, on serum lipid concentrations compared with a higher carbohydrate diet.**"**
**Note**	**Study start date:** April 2005<br>**Study end date:** November 2006

**Author, publication year**	**Kahleova 2021** [4]
**Title**	A plant-based diet in overweight adults in a 16-week randomized clinical trial: The role of dietary acid load
**DOI**	10.1016/j.clnesp.2021.05.015
**Trial identifier (if registered)**	NCT03698955
**Methods**	**Study design:** parallel randomized controlled trial<br>**Unit of randomisation:** individual<br>**Blinding:** "The participants were not blinded to their group assignment. The statistician was blinded to the interventions and group assignment."<br>**Number of study arms:** 2
**Participants**	**Country where trial was performed:** United States of America<br>**Location/Setting**: Washington, D.C.<br>**Number of study centres:** 1<br><br>**Sample size (No. of participants entering the study):** n = 223 (117/106)<br>**Dropouts/withdrawals:** Mostly for reasons unrelated to the study, 22 participants dropped out (5 from the intervention and 17 from the control group), leaving 223 (91.0%) study completers, who were included in the repeated measure ANOVA model.<br>**Sex**: both

	**Inclusion criteria (as reported):** aged 25-75 years, with a BMI between 28 and 40 kg/m^2<br><br>**Exclusion criteria (as reported):** history of diabetes, pregnancy or lactation, recent or current smoking, alcohol or drug abuse, current use of a vegan diet<br><br>**Characteristics of participants (e.g. occupation, health status):** BMI 28-40 kg/m^2<br>**Age of participants:** mean age 54.7
**Interventions/**	**Intervention:** low-fat vegan diet<br>**Comparator:** no diet changes<br><br>**Duration of intervention:** 16 weeks<br>**Duration of follow-up:** -<br>**Run-in period:** -
**Outcomes**	**Reported outcomes in full text of publication:** METs; weight; BMI; fat mass; lean mass; VAT volume; HbA1c; fasting plasma insulin; insulin at 30′, 60′, 120′, 180′; fasting plasma glucose; glucose at 30′, 60′, 120′, 180′; glucose sensitivity; rate sensitivity; PREDIM; HOMA; total cholesterol; triglycerides; HDL cholesterol; LDL cholesterol<br>**Timing of outcome assessment:** not applicable
**Publication details**	**Language of publication:** English<br>**Funding:** This work was funded by the Physicians Committee for Responsible Medicine.<br>**Conflict of interest** (quote)**:** " All authors except for AT and RH work or have worked for the Physicians Committee for Responsible Medicine in Washington, DC, a nonprofit organization providing educational, research, and medical services related to nutrition. Dr. Barnard is an Adjunct Professor of Medicine at the George Washington University School of Medicine. He serves without compensation as President of the Physicians Committee for Responsible Medicine and the Barnard Medical Center in Washington, DC. He writes books and articles and gives lectures related to nutrition and health and has received royalties and honoraria from these sources. "<br>**Publication status:** full article in peer-reviewed journal
**Stated aim for study**	**Quote:** "We previously evaluated the effects of a plant-based diet on a variety of metabolic indices in a 16-week randomized trial [8] and now evaluate the potential role of dietary acid load in changes in body weight, body composition, and insulin sensitivity."
**Note**	**Study start date:** February 2017<br>**Study end date:** February 2019

**Author, publication year**	**Kahleova 2018 (a)** [5]
**Title**	A Plant-Based High-Carbohydrate, Low-Fat Diet in Overweight Individuals in a 16-Week Randomized Clinical Trial: The Role of Carbohydrates
**DOI**	10.3390/nu10091302
**Trial identifier (if registered)**	NCT02939638
**Methods**	**Study design:** open parallel randomized controlled trial<br>**Unit of randomisation:** individual

	**Blinding:** "The participants were not blinded to their group assignment."<br>**Number of study arms:** 2
**Participants**	**Country where trial was performed:** United States of America<br>**Location/Setting**: Washington D.C.<br>**Number of study centres:** 1<br><br>**Sample size (No. of participants entering the study):** n = 75 (38/37)<br>**Dropouts/withdrawals:** 2 participants dropped out from the control group owing to personal reasons and 1 participant dropped out of the intervention group due to a family emergency.<br>**Sex**: both<br><br>**Inclusion criteria (as reported):** adults with a BMI between 28 and 40 kg/m^2<br><br>**Exclusion criteria (as reported):** comorbidities or recent use of medications that alter appetite or body weight, pregnancy, recent smoking or recreational drug use, evidence of an eating disorder, alcohol consumption above two drinks a day, unwillingness to comply with study requirements<br><br>**Characteristics of participants (e.g. occupation, health status):** BMI 28-40 kg/m^2<br>**Age of participants:** 53.2 ± 12.6
**Interventions/**	**Intervention:** low-fat vegan diet<br>**Comparator:** no diet changes<br>**Duration of intervention:** 16 weeks<br>**Duration of follow-up:** -<br>**Run-in period:**-
**Outcomes**	**Reported outcomes in full text of publication:** weight; fat mass; VAT volume; HOMA; changes in body composition<br>**Timing of outcome assessment:** not applicable
**Publication details**	**Language of publication:** English<br>**Funding:** "This work was funded by the Physicians Committee for Responsible Medicine."<br>**Conflict of interest** (quote)**:** "Barnard is an Adjunct Associate Professor of Medicine at the George Washington University School of Medicine and serves without financial compensation as president of the Physicians Committee for Responsible Medicine and Barnard Medical Center. He writes books and gives lectures related to nutrition and health and has received royalties and honoraria from these sources. Kahleova is the Director of Clinical Research of the Physicians Committee for Responsible Medicine, a nonprofit organization conducting research and education in nutrition. Dort has worked for the Physicians Committee for Responsible Medicine. Holubkov does not declare any conflict of interest."<br>**Publication status:** full article in peer-reviewed journal
**Stated aim for study**	**Quote:** "Our hypothesis was that high carbohydrate and fiber intakes in the context of a plant-based diet will be associated with weight loss, reduction in fat mass, and decrease in insulin resistance."
**Note**	**Study start date:** October 2016<br>**Study end date:** June 2017

**Author, publication year**	**Neacsu 2014** [6]
**Title**	Appetite control and biomarkers of satiety with vegetarian (soy) and meat-based high-protein diets for weight loss in obese men: a randomized crossover trial
**DOI**	10.3945/ajcn.113.077503
**Trial identifier (if registered)**	NCT02080325
**Methods**	**Study design:** crossover randomized controlled trial<br>**Unit of randomisation:** individual<br>**Blinding:**<br>**Number of study arms:** 2
**Participants**	**Country where trial was performed:** United Kingdom<br>**Location/Setting**: Aberdeen<br>**Number of study centres:** 1<br><br>**Sample size (No. of participants entering the study):** n = 40 (20/20)<br>**Dropouts/withdrawals:** No subjects withdrew from the study<br>**Sex**: male<br><br>**Inclusion criteria (as reported):** overweight or obese men [BMI (in kg/m^2) > 27]<br><br>**Exclusion criteria (as reported):** all subjects should not have existing medical conditions or medications that could influence their appetite or mood<br><br>**Characteristics of participants (e.g. occupation, health status):** overweight or obese men, BMI > 27<br>**Age of participants:** mean age 51
**Interventions/**	**Intervention:** Vegetarian HPWL (soy protein or soy-textured vegetable protein)<br>**Comparator:** Meat-HPWL<br>**Duration of intervention:** 2 weeks<br>**Duration of follow-up:** -<br>**Run-in period:** -
**Outcomes**	**Reported outcomes in full text of publication:** albumin, cholesterol, hydroxybutyrate, LDL cholesterol, triglyceride, LDL:HDL cholesterol ratio, total HDL cholesterol ratio, HDL cholesterol, glucose, urea, creatine, total bilirubin, plasma amino acid concentration<br>**Timing of outcome assessment:** not applicable
**Publication details**	**Language of publication:** English<br>**Funding:** no mentioned<br>**Conflict of interest** (quote)**:** not mentioned<br>**Publication status:** full article in peer-reviewed journal
**Stated aim for study**	**Quote:** "The current study compares hunger and appetite response in healthy obese men who consumed a fixed diet for weight loss as a Soy-HPWL or a Meat-HPWL diet in a controlled dietary intervention setting."
**Note**	**Study start date:** December 2009<br>**Study end date:** December 2010

Author, publication year	**Li 2016** [7]
**Title**	Effects of Dietary Protein Source and Quantity during Weight Loss on Appetite, Energy Expenditure, and Cardio-metabolic Responses
**DOI**	10.3390/nu8020063
**Trial identifier (if registered)**	NCT01005563
**Methods**	**Study design:** parallel randomized controlled trial<br>**Unit of randomisation:** individual<br>**Blinding:** not mentioned<br>**Number of study arms:** 2
**Participants**	**Country where trial was performed:** United States of America<br>**Location/Setting**: Lafayette, Louisiana<br>**Number of study centres:** 1<br><br>**Sample size (No. of participants entering the study):** n = 34 (17/17)<br>**Dropouts/withdrawals:** meat diet group: 5 (financial issues, underestimated the commitment to the study, dietary non-compliance vegetarian group: 8 (relocation, financial issues, underestimated the commitment to the study, dietary non-compliance)<br>**Sex**: both<br><br>**Inclusion criteria (as reported):** 21 years and older; body mass index (BMI) range 27.0–36.9 kg/m^2; non-smoking; weight stable (_3 kg) and stable habitual physical activity patterns during previous 3 months; energy need for weight maintenance 2000–3150 kcal/day; not dietary restrained, 14 on hunger scale; post-menopausal, or regularly menstruating women not pregnant or lactating; no acute illness; absence of diabetes mellitus, hypertension, and chronic diseases or use of medications known to influence protein or energy metabolism; blood profile (from the screening visit) within 10% of clinical normalcy (glucose, lipid-lipoprotein profile, liver function enzymes, creatinine); and willingness to eat study foods and able to travel to the testing facility.<br><br>**Exclusion criteria (as reported):** not mentioned in the text, NCT said: Age: < 21 years, Body mass index: outside of the 27.0-36.9 kg/m^2 range, Smoker (currently or within the last 6 months), Gained or lost > 3.0 kg (7 lbs) within the last 3 months, Energy need for weight maintenance < 1950 or > 2750 kcal/day, Dietary restrained (≥ 14 on Three Factor Eating Questionnaire), Pregnant, lactating, or non-menstruating women, Clinically diagnosed as a diabetic, or with liver or kidney disease/dysfunction, or osteoporosis, Clinically abnormal blood profiles as identified by our study physician, Arthur Rosen, MD, Hypertensive, Taking medications (currently or within the last 3 months) known to influence appetite or metabolism, Allergies to eggs, Lactose intolerance, Unwillingness to eat study foods, Inability to travel to testing facility<br><br>**Characteristics of participants (e.g. occupation, health status):** female and male, BMI 27.0–36.9 kg/m^2<br>**Age of participants:** mean age 53.5
**Interventions/**	**Intervention:** Vegetarian HPWL (Soy-HPWL)<br>**Comparator:** Meat-HPWL<br>**Duration of intervention:** 14 weeks

	**Duration of follow-up:** -<br>**Run-in period:** -
**Outcomes**	**Reported outcomes in full text of publication:** total cholesterol, HDL cholesterol, LDL cholesterol, triacylglycerol, APO-A1, APO-B, fasting glucose, fasting insulin, HOMA-IR, HOMA-ß, glucose AUC, insulin AUC, blood pressure status, fasting renal status, resting energy expenditure<br>**Timing of outcome assessment:** not applicable
**Publication details**	**Language of publication:** English<br>**Funding:** not mentioned<br>**Conflict of interest** (quote)**:** "The authors did not have any personal or financial conflicts of interest. The funding sponsors had no role in the design of the study; in the collection, analyses, or interpretation of data; in the writing of the manuscript, and in the decision to publish the results."<br>**Publication status:** full article in peer-reviewed journal
**Stated aim for study**	**Quote:** "The purpose of this study was to assess the effects of dietary protein intakes across the ADMR with beef/pork or soy/legume as the predominant protein source on daily and postprandial appetitive responses in overweight/obese adults who were acclimated to an energy-restricted diet."
**Note**	**Study start date:** January 2010<br>**Study end date:** September 2011

**Author, publication year**	**Mahon 2007** [8]
**Title**	Protein Intake during Energy Restriction: Effect on Body Composition and Markers of Metabolic and Cardiovascular Health in Postmenopausal Women
**DOI**	10.1080/07315724.2007.10719600
**Trial identifier (if registered)**	Not mentioned
**Methods**	**Study design:** parallel randomized controlled trial<br>**Unit of randomisation:** individual<br>**Blinding:** not mentioned<br>**Number of study arms:** 3
**Participants**	**Country where trial was performed:** United States of America<br>**Location/Setting**: Cleveland<br>**Number of study centres:** 1<br><br>**Sample size (No. of participants entering the study):** n = 43 (29/14)<br>**Dropouts/withdrawals:** "4 women dropped out during PRE resulting", "1 woman dropped out for personal reasons, another was dismissed due to her need to take medication that made her ineligible to participate, and 1 dropped due to non-adherence to the diet"<br>**Sex**: female<br><br>**Inclusion criteria (as reported):** postmenopausal women, BMI 29.6 ± 0.8 kg/m^2<br><br>**Exclusion criteria (as reported):** male, BMI < 25 and > 34 kg/m^2, age < 50 y or > 80 y, < 2 y postmenopausal, smoker and clinically abnormal kidney, liver, or heart function. Subjects could not be individuals with diabetes or unstable thyroid

	disease, in abnormal protein or hematological status, nor receiving insulin replacement therapy or anti-inflammatory steroid medications.<br><br>**Characteristics of participants (e.g. occupation, health status):** postmenopausal healthy women<br>**Age of participants:** mean age 58
**Interventions/**	**Intervention:** lacto-ovo vegetarian basal diet plus 250 kcal/d of either beef (BEEF), chicken (CHICKEN), or carbohydrate/fat foods (CARB (lacto-ovo)<br>**Comparator:** no diet changes<br>**Duration of intervention:** 9 weeks<br>**Duration of follow-up:** -<br>**Run-in period:** -
**Outcomes**	**Reported outcomes in full text of publication:** body mass, fat mass, body fat, fat-free mass, BMI, cholesterol, LDL, HDL, triacylglycerol, CHOL:HDL, CRP<br>**Timing of outcome assessment:** not applicable
**Publication details**	**Language of publication:** English<br>**Funding:** not mentioned<br>**Conflict of interest** (quote)**:** not mentioned<br>**Publication status:** full article in peer-reviewed journal
**Stated aim for study**	**Quote:** "The primary purpose of this study was to compare the short term effects of two ER, MHP diets that differed in protein source (either beef or chicken as the primary protein source, both 26% of energy) vs. a lacto-ovo vegetarian LP diet that differed in protein quantity (16% of energy) on changes in body mass and body composition. Secondary aims included analyses of lipoprotein-lipid profile, CRP, glucose, insulin, leptin and adiponectin concentrations in overweight and mildly obese postmenopausal women."
**Note**	**Study start date:** not mentioned<br>**Study end date:** not mentioned

**Author, publication year**	**Sofi 2018** [9]
**Title**	Low-Calorie Vegetarian Versus Mediterranean Diets for Reducing Body Weight and Improving Cardiovascular Risk Profile: CARDIVEG Study (Cardiovascular Prevention With Vegetarian Diet)
**DOI**	10.1161/CIRCULATIONAHA.117.030088
**Trial identifier (if registered)**	NCT02641834
**Methods**	**Study design:** crossover randomized controlled trial<br>**Unit of randomisation:** individual<br>**Blinding:** not mentioned<br>**Number of study arms**: 2
**Participants**	**Country where trial was performed:** Italy<br>**Location/Setting**: Florence<br>**Number of study centres:** 1<br><br>**Sample size (No. of participants entering the study):** n = 118 (60/58)<br>**Dropouts/withdrawals:** "During the study, 18 (15.3%) participants reported a less-than-optimal compliance to the prescribed diets and were excluded at different time points from the study"<br>**Sex**: both

	**Inclusion criteria (as reported):** Eligibility criteria included being overweight (body mass index [BMI] ≥25 kg/m^2) and the simultaneous presence of ≥1 of the following criteria defined by the guidelines for cardiovascular disease prevention of the European Society of Cardiology:15 total cholesterol levels >190 mg/dL, low-density lipoprotein (LDL) cholesterol levels > 115 mg/dL, triglyceride levels >150 mg/dL, and glucose levels >110 but <126 mg/dL<br><br>**Exclusion criteria (as reported):** "Participants were excluded if they were taking medications for any reason, had a serious illness or an unstable condition, were pregnant or nursing, were participating or had participated in a weight loss treatment program in the last 6 months, or were following or had followed a food profile that, to a certain extent,<br>excluded meat, poultry, or fish in the last 6 months."<br><br>**Characteristics of participants (e.g. occupation, health status):** -<br>**Age of participants:** mean age 51.1
**Interventions/**	**Intervention:** AHA Diet<br>**Comparator:** PB no added fat diet<br>**Duration of intervention:** 3 months<br>**Duration of follow-up:** -<br>**Run-in period:** 2 weeks
**Outcomes**	**Reported outcomes in full text of publication:** weight, BMI, fat mass, changes in biochemical parameters, changes in oxidative stress parameters, changes in inflammatory parameters<br>**Timing of outcome assessment:** not applicable
**Publication details**	**Language of publication:** English<br>**Funding:** not mentioned<br>**Conflict of interest** (quote)**:** not mentioned<br>**Publication status:** full article in peer-reviewed journal
**Stated aim for study**	**Quote:** "Our aim was to compare, in a population of omnivorous individuals living in a low-risk (for cardiovascular disease) European country, the effects of a 3-month period on a low-calorie Vd compared with a low-calorie Mediterranean diet (MD) on several markers of cardiovascular disease risk"
**Note**	**Study start date:** March 2014<br>**Study end date:** June 2015

**Author, publication year**	**Macknin 2015** (children) [10]
**Title**	Plant-based No Added Fat or American Heart Association Diets, Impact on Cardiovascular Risk in Obese Hypercholesterolemic Children and Their Parents
**DOI**	10.1016/j.jpeds.2014.12.058
**Trial identifier (if registered)**	NCT02641834
**Methods**	**Study design:** prospective randomized controlled trial<br>**Unit of randomisation:** individual<br>**Blinding:** not mentioned<br>**Number of study arms:** 3
**Participants**	**Country where trial was performed:** United States of America

	**Location/Setting**:<br>**Number of study centres:** 1<br><br>**Sample size (No. of participants entering the study):** n = 28 (14/14)<br>**Dropouts/withdrawals:** "The sample size of 15 adults and children per group was calculated to substantially exceed, even with a 20% drop-out rate, the 6–7 patients per group required to provide a power of 90% at a significance level of 0.05 to detect the within-group changes from baseline in total cholesterol described previously (mean ± standard deviation decrease of 60 ± 26 mg/dL) versus a null hypothesis mean decrease of 25 ± 26mg/dL."<br>**Sex**: both<br><br>**Inclusion criteria (as reported):** children ages 9-18 with BMI>95% and total cholesterol > 169 mg/dL and one of their parents (obese, hypercholesterolemic children age 9-18 old and one of their patients)<br><br>**Exclusion criteria (as reported):** Excluded: not meeting inclusion criteria, n = 14; declined to participate, n = 13; interested but enrolment closed, n = 37; consented but withdrew before randomization, n = 1); excluded from analysis who did not attend last session, therefore no end of study data, n = 2)<br><br>**Characteristics of participants (e.g. occupation, health status):** obese, hypercholesterolemic children, BMI > 95% and total cholesterol > 169 mg/dL<br>**Age of participants:** mean age 15
**Interventions/**	**Intervention:** plant-based no added fat diet (PB)<br>**Comparator:** American Heart Association Diet (AHA)<br>**Duration of intervention:** 4 weeks<br>**Duration of follow-up:** -<br>**Run-in period:** n-
**Outcomes**	**Reported outcomes in full text of publication:** BMI, blood pressure, weight, waist circumferences, PAQ, cholesterol, triglyceride, HDL, LDL, glucose, CRP, alanine aminotransferase, AST, IL-6, MPO, HgbA1c, insulin<br>**Timing of outcome assessment:** not applicable
**Publication details**	**Language of publication:** English<br>**Funding: "**Supported by National Center for Advancing Translational Sciences of the National Institutes of Health (UL1TR000439) and Research Program Committee (2012-1063 4) and Pediatric Research Fund Grants from the Cleveland Clinic.<br>**Conflict of interest** (quote)**:** " The authors declare no conflicts of interest."<br>**Publication status:** full article in peer-reviewed journal
**Stated aim for study**	**Quote:** "Our hypothesis was that both groups would show improvement in the studied outcomes and the improvements might be greater for the PB than AHA."
**Note**	**Study start date:** April 2013<br>**Study end date:** May 2013

Table S3.1. Characteristics of studies awaiting classification

Table S3.1.2. Registered trials without publication

**Study identifier**	**NCT03475368 2018** [11]

**Study title:**	Interactions Between Diet, Intestinal Microbiota and Metabolomics<br>**Acronym: –**
**Official title:**	Interactions Between Diet, Intestinal Microbiota and Metabolomics
**Methods:**	**Type of trial:** interventional<br>**Allocation:** randomised<br>**Intervention model:** parallel assignment<br>**Masking:** open label<br>**Primary purpose:** treatment
**Participants:**	**Age:** 18 years to 60 years Adults<br>**Enrollment:** 60<br>**Inclusion criteria:**<br>• No major comorbidities with a life expectancy less than 12 months;<br>• BMI between 29 and 33 kg/m^2<br>• Serum cholesterol 200-260 mg/dL<br>• Serum triglycerides > 150 mg/dL<br><br>**Exclusion criteria:**<br>• Antibiotics or prebiotics in the 3 months before the enrolment;<br>• Use of statins or other medications for lowering cholesterol;<br>• Menopause;<br>• Previous history of cancer
**Interventions**	**Intervention(s):**<br>• a vegetarian diet (i.e. without animal products, except milk and eggs)<br>**Comparator(s):**<br>• low carbs diet (i.e. with a limited amount of carbohydrates)<br>• a traditional Mediterranean diet (i.e. with low glycaemic index carbohydrates and vegetables)
**Starting date**	**Trial start date:** 2018-04-01<br>**Trial completion date:** 2019-04-01<br>**Status:** unknown
**Contact information**	**Responsible party/principal investigator:** Gioacchino Leandro, Azienda Ospedaliera Specializzata in Gastroenterologia Saverio de Bellis, Italy
**Stated purpose of study**	**Quote:** "Therefore the clinical Nutrition Clinic of the IRCCS De Bellis in Castellana Grotte (BA) proposes to check if changes in the intestinal microbiota correlate, not only with anthropometric and clinical-laboratory parameters, but also with the typical symptoms of irritable bowel syndrome (IBS), a functional pathology very widespread with the advent of the modern era, in which, a diet rich in sugars and proteins of animal origin and poor in plant foods, is unfortunately common also in the areas of the Mediterranean basin."
**Note**	Recruitment Status: unknown<br>First Posted: 23 March, 2018<br>Last Update Posted: 01 May, 2018

**Study identifier**	**NCT04222894 2020** [12]
**Study title:**	Hospital Workplace Nutrition Study<br>**Acronym: –**
**Official title:**	Hospital Workplace Nutrition Study (Sibley Memorial Hospital)

**Methods:**	**Type of trial:** interventional<br>**Allocation:** randomised<br>**Intervention model:** Parallel Assignment<br>**Masking:** None (Open Label)<br>**Primary purpose:** Treatment
**Participants:**	**Age:** 18 Years and older (Adult, Older Adult)<br>**Enrollment:** 22<br>**Inclusion criteria:**<br>• Employee of Sibley hospital<br>• Male or female<br>• Age at least 18 years<br>• Have a BMI > 25 kg/m^2<br>• Ability and willingness to participate in all components of the study<br>• A willingness to follow a plant-based diet for the duration of the study<br>• A willingness to attend weekly classes for the duration of the study<br>• A willingness to keep physical activity level consistent throughout the duration of the study<br>**Exclusion criteria:**<br>• Diabetes mellitus type 1 or history of any endocrine condition that would affect body weight, such as a pituitary abnormality or Cushing's syndrome<br>• Smoking during the past six months<br>• Alcohol consumption of more than 2 drinks per day or the equivalent, episodic increased drinking (e.g., more than 2 drinks per day on weekends), or a history of alcohol abuse or dependency followed by any current use<br>• Current or unresolved past drug abuse<br>• Pregnancy or plans to become pregnant in the next 12 weeks<br>• Intention to leave hospital employment in the next 12 weeks<br>• Unstable medical or psychiatric status<br>• Evidence of an eating disorder<br>• Lack of English fluency<br>• Inability to maintain current medication regimen
**Interventions**	**Intervention(s):**<br>• Plan-based/vegan diet<br>Weekly instructions will be given to the participants in the intervention group about following vegan diet.<br>**Comparator(s):**<br>• Control diet<br>Participants will be asked to continue their usual diets for the 12-week study period.
**Starting date**	**Trial start date:**<br>**Trial completion date:**
**Contact information**	**Responsible party/principal investigator:** Neal D Barnard, MD, President
**Stated purpose of study**	**Quote:** "The purpose of this study is to evaluate the effects of a plant-based diet on body weight, blood pressure, and plasma lipid concentrations, as part of a hospital workplace program."
**Note**	Recruitment Status: Completed<br>First Posted: 10 January 2020<br>Last Update Posted: 19 April 2022

**Study identifier**	**CTRI/2020/08/027057 2020** [13]

**Study title:**	To study the role of whole food, plant-based diet in reducing weight in obese patients in comparison with the standard weight reducing diet<br>**Acronym: –**
**Official title:**	Role of a plant-based diet in weight reduction among obese patients
**Methods:**	**Type of trial:** Interventional (Nutraceutical)<br>**Allocation:** Randomized (Stratified block randomization)<br>**Intervention model:** Parallel Group Trial<br>**Masking:** Open Label<br>**Primary purpose:** Obesity, unspecified
**Participants:**	**Age:** 18-99 years (Gender: both)<br>**Enrollment: 50**<br>**Inclusion criteria:**<br>• obesity defined as BMI greater than 30 kg/m^2;<br>• All consenting adult patients presenting to CMCH Vellore General Medicine OPD with class II obesity defined as BMI greater than 30 kg/m^2,<br>• Patient giving a written consent for the study<br>**Exclusion criteria:**<br>Children (less than 18 years) Patients with diagnosed with obesity due to secondary cause as primary hypothyroidism, Cushing's syndrome, or any syndromic obesity. Patients with chronic kidney disease with eGFR less than 30ml per min<br>Patients on Warfarin, acitrom, or any vitamin K antagonists. Patients presenting with obesity for bariatric surgery. Pregnant women and women who gave birth within 3 months. Non consenting adults.
**Interventions**	**Intervention(s):**<br>• Whole food plant based diet for 12 weeks<br>**Comparator(s):**<br>• Standard weight reducing diet for 12 weeks<br>• Primary outcomes<br>Change in weight<br>• Secondary outcomes:<br>Change in BMI<br>Change in waist circumference<br>Change in waist to hip ratio<br>Change in HbA1c<br>Change in inflammatory marker hsCRP<br>Change in fasting lipid profile<br>Change in blood pressure<br>Change in insulin resistance with HOMA-IR system<br>Compliance to both the diets
**Starting date**	**Trial start date:** 14/08/2020 (Date of first enrollment (India)<br>**Trial completion date:** No Date Specified
**Contact information**	**Responsible party/principal investigator:** Dr Jim Ounny John, MD Student; Dr Samuel George Hansdak Professor and Guide (scientific query); Dr Samuel George Hansdak Professor and Guide (public query)
**Stated purpose of study**	**Quote:** "Plant based diet could have benefits in Obesity as suggested by various human studies.This is a study aimed at comparing the effect of plant-based diet and the standard weight reducing diet advised to subjects with obesity."
**Note**	Recruitment Status: Not Yet Recruiting (Global, Not Yet Recruiting (India)<br>First Posted: No Date Specified<br>Last Update Posted: No Date Specified

Study identifier	**APOA2 Gene, Diet, Inflammation and Gut Health** [14]
**Study title:**	**Acronym: –**
**Official title:**	APOA2 Gene, Diet, Inflammation and Gut Health
**Methods:**	**Type of trial:** interventional<br>**Allocation:** randomised<br>**Intervention model:** Crossover Assignment<br>**Masking:** Single (Investigator)<br>**Primary purpose:** Prevention
**Participants:**	**Age:** 18 Years and older (Adult, Older Adult)<br>**Enrolment:** 37<br>**Inclusion criteria:**<br>• Men and women<br>• 18 years or older<br>• Women who are not pregnant<br>• A BMI ranging between 27 and 34<br>**Exclusion criteria:**<br>• Unexplained elevation in serum transaminases (i.e. >1.5 times the upper limit of normal) or with evidence of active liver disease, including primary biliary cirrhosis or pre-existing gallbladder disease<br>• Severe renal dysfunction (serum creatinine > 2.0 mg/dL)<br>• Excessive alcohol consumption (> 2 drinks/day)<br>• Preexisting cardiovascular disease (CVD)<br>• Stable exertional angina pectoris requiring sublingual nitro-glycerine within the prior 3 months<br>• Uncontrolled type 2 diabetes (T2D) (fasting glucose > 126 mg/dL) or other significant endocrine disease.<br>• Uncontrolled hypertension (systolic blood pressure >1 80 mmHg or diastolic blood pressure > 100 mmHg).<br>• History of pancreatitis within 1 yr. prior to screening.<br>• Subjects on lipid lowering or diabetes medications.<br>• Smoking<br>• Pregnancy<br>• Body mass index (BMI) below 27 or greater than 34 kg/m^2<br>• Participants will also be excluded for drug abuse, extreme dietary habits, multiple food allergies, extreme levels of physical or athletic activity, or by changes in body weight >20 lbs. during the last 6 months<br>• Current use of antibiotics or during the previous 4 weeks.<br>• Inability to follow any of the experimental diets (including being vegetarian) or to perform the sampling required for this study<br>• Use of herbal supplements that may alter the gut microflora<br>• Autoimmune diseases<br>• Recent colonoscopy (within the previous two months)<br>• Use of antidiarrheal medication<br>• Thyroid diseases<br>• Use of omega-3 supplements (unless it is discontinued one month prior to the beginning of the study).
**Interventions**	**Intervention(s):**<br>• Plant Diet: during one-week participants will receive plant products enriched in fibre and complex carbohydrates<br>**Comparator(s):**

	• Animal Diet: during one-week participants will receive food products enriched in animal products and with high content of fat and protein
**Starting date**	**Trial start date:** 2017-10-13<br>**Trial completion date:** 2021-12-31
**Contact information**	**Responsible party/principal investigator:** Jose M Ordovas, PH, JM-USDA HNRCA at Tufts University
**Stated purpose of study**	**Quote: "**The main purpose of this four-week study is to examine diet induced gene-nutrient interaction, with a focus on gut health, gut microbiota and inflammation in individuals who have either the CC or the TT form within a specific variant of the APOA2 Gene. The (2) one-week study diets, one plant based and the other animal based are separated by a (1) week return to your regular habitual without probiotic or prebiotic food products**."**
**Note**	Recruitment Status: Unknown<br>First Posted: 26 October 2017<br>Last Update Posted: 19 April 2021

Table S3.1.3. Conference abstracts and posters

**Study**	**Gratz 2020** [15]<br>Comparison of meat versus soya based high-protein diets on faecal microbiota and microbial metabolites
**Methods**	**Study design:** clinical trial
**Participants**	**Inclusion criteria:** obese men (n = 20)<br>**Exclusion criteria: –**<br>**Setting:** not mentioned<br>**Age:** not mentioned<br>**Country where trial was performed:** not mentioned
**Interventions**	**Intervention(s):**<br>• High-protein weight loss diet, beef<br>**Comparator(s):**<br>• High-protein weight loss diet, soya<br>**Duration of intervention:** 2 weeks
**Outcomes**	**Reported outcomes in the abstract:**<br>• faecal microbial DNA was extracted for qPCR bacterial profiling, short chain fatty acid (SCFA) analysis by GC and metabolite analysis by LC-MS/MS
**Identification**	**Trial identifier:** not mentioned (Proceedings of the Nutrition Society (2020), 79 (OCE3), E781)<br>**Trial terminated early:** no
**Publication details**	**Language of publication:** English<br>**Funding**: not mentioned<br>**Publication status:** abstract
**Stated aim for study**	**Quote:** "High-protein, low carbohydrate diets are popular and efficient weight loss regimes, but we have previously shown that meat based high-protein diets had a negative effect on faecal metabolites and gut microbiota. We therefore conducted a study to investigate whether vegetarian high-protein diets would have the same effect."
**Note**	No primary outcomes, other outcome: microbiota composition

**Study**	**Keller 2020** [16]

	Impact of a Boxed Meal Facilitated Plant-based Diet on Cardiometabolic Endpoints: A Clinical Trial
**Methods**	**Study design:** prospective, controlled, parallel-designed trial
**Participants**	**Inclusion criteria:**<br>• n = 32<br>• BMI > 25 kg/m^2<br>**Exclusion criteria: –**<br>**Setting:** not mentioned<br>**Age:** not mentioned (average: 33.3 ± 7.8 years)<br>**Country where trial was performed:** not mentioned
**Interventions**	**Intervention(s):**<br>• boxed meal facilitated PBD (f-PBD)<br>"Participants had the option to consume breakfast and lunch according to their adopted diet at the military dining facility. Dinner was facilitated by shipping a commercially available plant-based or standard omnivorous boxed meal kit to participants as appropriate."<br>**Comparator(s):**<br>• Standard Omnivorous Diet (f-SOD)<br>**Duration of intervention:** 4 weeks
**Outcomes**	**Reported outcomes in the abstract:**<br>• changes in weight, BMI, Change in LDL-c, Systolic BP
**Identification**	**Trial identifier:** not mentioned<br>**Trial terminated early:** no
**Publication details**	**Language of publication:** English<br>**Funding**: not mentioned<br>**Publication status:** abstract
**Stated aim for study**	**Quote:** "We compared cardiometabolic changes between a boxed meal facilitated PBD (f-PBD) and Standard Omnivorous Diet (f-SOD)."
**Note**	–

References

1. Barnard, N.D.; Scialli, A.R.; Turner-McGrievy, G.; Lanou, A.J.; Glass, J. The effects of a low-fat, plant-based dietary intervention on body weight, metabolism, and insulin sensitivity. *American Journal of Medicine* **2005**, *118*, 991-997.
2. Barnard, N.D.; Alwarith, J.; Rembert, E.; Brandon, L.; Nguyen, M.; Goergen, A.; Horne, T.; do Nascimento, G.F.; Lakkadi, K.; Tura, A.; et al. A Mediterranean Diet and Low-Fat Vegan Diet to Improve Body Weight and Cardiometabolic Risk Factors: A Randomized, Cross-over Trial. *J Am Coll Nutr* **2021**, 1-13, doi:10.1080/07315724.2020.1869625.
3. Jenkins, D.J.; Wong, J.M.; Kendall, C.W.; Esfahani, A.; Ng, V.W.; Leong, T.C.; Faulkner, D.A.; Vidgen, E.; Paul, G.; Mukherjea, R.; et al. Effect of a 6-month vegan low-carbohydrate ('Eco-Atkins') diet on cardiovascular risk factors and body weight in hyperlipidaemic adults: a randomised controlled trial. *BMJ Open* **2014**, *4*, e003505, doi:10.1136/bmjopen-2013-003505.
4. Kahleova, H.; McCann, J.; Alwarith, J.; Rembert, E.; Tura, A.; Holubkov, R.; Barnard, N.D. A plant-based diet in overweight adults in a 16-week randomized clinical trial: The role of dietary acid load. *Clin Nutr ESPEN* **2021**, *44*, 150-158, doi:10.1016/j.clnesp.2021.05.015.
5. Kahleova, H.; Dort, S.; Holubkov, R.; Barnard, N.D. A Plant-Based High-Carbohydrate, Low-Fat Diet in Overweight Individuals in a 16-Week Randomized Clinical Trial: The Role of Carbohydrates. *Nutrients* **2018**, *10*, 14, doi:https://dx.doi.org/10.3390/nu10091302.
6. Neacsu, M.; Fyfe, C.; Horgan, G.; Johnstone, A.M. Appetite control and biomarkers of satiety with vegetarian (soy) and meat-based high-protein diets for weight loss in obese men: a randomized crossover

trial. *American Journal of Clinical Nutrition* **2014**, *100*, 548-558, doi:https://dx.doi.org/10.3945/ajcn.113.077503.
7. Li, J.; Armstrong, C.L.; Campbell, W.W. Effects of Dietary Protein Source and Quantity during Weight Loss on Appetite, Energy Expenditure, and Cardio-Metabolic Responses. *Nutrients* **2016**, *8*, 63, doi:https://dx.doi.org/10.3390/nu8020063.
8. Mahon, A.K.; Flynn, M.G.; Stewart, L.K.; McFarlin, B.K.; Iglay, H.B.; Mattes, R.D.; Lyle, R.M.; Considine, R.V.; Campbell, W.W. Protein intake during energy restriction: effects on body composition and markers of metabolic and cardiovascular health in postmenopausal women. *Journal of the American College of Nutrition* **2007**, *26*, 182-189.
9. Sofi, F.; Dinu, M.; Pagliai, G.; Cesari, F.; Gori, A.M.; Sereni, A.; Becatti, M.; Fiorillo, C.; Marcucci, R.; Casini, A. Low-Calorie Vegetarian Versus Mediterranean Diets for Reducing Body Weight and Improving Cardiovascular Risk Profile: CARDIVEG Study (Cardiovascular Prevention With Vegetarian Diet). *Circulation* **2018**, *137*, 1103-1113, doi:https://dx.doi.org/10.1161/CIRCULATIONAHA.117.030088.
10. Macknin, M.; Kong, T.; Weier, A.; Worley, S.; Tang, A.S.; Alkhouri, N.; Golubic, M. Plant-based, no-added-fat or American heart association diets: Impact on cardiovascular risk in obese children with hypercholesterolemia and their parents. *Journal of Pediatrics* **2015**, *166*, 953-959.e953, doi:10.1016/j.jpeds.2014.12.058.
11. Nct; Azienda Ospedaliera Specializzata in Gastroenterologia Saverio de, B. Interactions Between Diet, Intestinal Microbiota and Metabolomics. **2018**. Available online: https://www.clinicaltrials.gov/study/NCT03475368 (accessed on 13 Sep 2024).
12. Nct; Physicians Committee for Responsible, M. Hospital Workplace Nutrition Study. **2020**. Available online: https://clinicaltrials.gov/study/NCT04222894 (accessed on 13 Sep 2024).
13. Ctri; Dr Jim Ounny, J. To study the role of whole food, plant based diet in reducing weight in obese patients in comparison with the standard weight reducing diet. **2020**. Available online: http://www.ctri.nic.in/Clinicaltrials/pmaindet2.php?trialid=45703 (accessed on 13 Sep 2024).
14. Tufts, U. APOA2 Gene, Diet, Inflammation and Gut Health. **2017**. Available online: https://clinicaltrials.gov/study/NCT03322449 (accessed on 13 Sep 2024).
15. Gratz, S.W.; Scobbie, L.; Richardson, A.J.; Zhang, X.; Fyfe, C.; Farquharson, F.M.; Duncan, G.; Filipe, J.; Zhu, W.Y.; Johnstone, A.M.; et al. Comparison of meat versus soya based high-protein diets on faecal microbiota and microbial metabolites. *Proceedings of the Nutrition Society* **2020**, *79*, doi:10.1017/S0029665120007673.
16. Keller, A.L.; Ziad, S.; Stephens, A.M.; Tesch, E.A.; Sky, J.; Shih, E.; Brooks, K.A.; Howarth, V.E.; Shah, S.A. Impact of a Boxed Meal Facilitated Plant-based Diet on Cardiometabolic Endpoints: A Clinical Trial. *Circulation* **2020**, *142*, doi:10.1161/circ.142.suppl_3.14055.
