# Supplementary material for: Health Effects of Plant-Based Diets in People with Overweight or Obesity: A Systematic Review and Meta-Analysis
Source: Nutrients. 2026 Jun 19;18(12):1987. doi: 10.3390/nu18121987 (PMC13304861; doi:10.3390/nu18121987)
Supplement: Supplementary file 1 [file nutrients-18-01987-s001.zip › Supplementary File S4_Nutrition table.pdf]

Supplementary file S4

Table S4: Nutrition table

No.	Author, publication date	Intervention diet	Control diet
1	Barnard 2005 [1-4]	LFV	NCEP
2	Barnard 2021 [5]	LVF	MED
3	Jenkins 2014 [6,7]	LC-vegan	LOV-HC
4	Kahleova, 2021 [8-13]	LVF	Control
5	Kahleova 2018 [14-16]	LFV-2	Control
6	Neacsu 2014 [17]	soy-HPWL	Meat-HPWL
7	Li 2016 [18]	ER-P-LOV	Control
8	Mahon 2007 [19]	ER-LOV	ER
9	Sofi 2018 [20]	LOV-HC	MED
10	Macknin 2015 [21]	LVF-3	AHA

Abbreviations: AHA: American Heart Association Diet, ER: energy restricted diet, ER-LOV: energy restricted- lacto-ovo vegetarian diet, ER-P-LOV: Energy restricted-protein-lacto-ovo vegetarian diet, LC-vegan: Low-carbohydrate plant based diet, LFV: low-fat vegan diet, LOV-HC: lacto-ovo vegetarian diet-hypocaloric, LVF-2: Low-fat vegan diet-2, LVF-3: low fat vegan diet-3, MED: Mediterranean diet, Meat-HPWL Meat-high protein weight loss diet, NCEP: National Education Cholesterol Program, soy-HPWL: soy-high protein weight loss diet

References

1. Barnard, N.D.; Scialli, A.R.; Turner-McGrievy, G.; Lanou, A.J.; Glass, J. The effects of a low-fat, plant-based dietary intervention on body weight, metabolism, and insulin sensitivity. *American Journal of Medicine* **2005**, *118*, 991-997, https://doi.org/10.1016/j.amjmed.2005.03.039
2. Barnard, N.D.; Scialli, A.R.; Turner-McGrievy, G.; Lanou, A.J. Acceptability of a low-fat vegan diet compares favorably to a step II diet in a randomized, controlled trial. *Journal of Cardiopulmonary Rehabilitation* **2004**, *24*, 229-235, https://doi.org/10.1097/00008483-200407000-00004
3. Turner-McGrievy, G.M.; Barnard, N.D.; Scialli, A.R.; Lanou, A.J. Effects of a low-fat vegan diet and a Step II diet on macro- and micronutrient intakes in overweight postmenopausal women. *Nutrition* **2004**, *20*, 738-746, https://doi.org/10.1016/j.nut.2004.05.005
4. Turner-McGrievy, G.M.; Barnard, N.D.; Scialli, A.R. A two-year randomized weight loss trial comparing a vegan diet to a more moderate low-fat diet. *Obesity* **2007**, *15*, 2276-2281, https://doi.org/10.1038/oby.2007.270
5. Barnard, N.D.; Alwarith, J.; Rembert, E.; Brandon, L.; Nguyen, M.; Goergen, A.; Horne, T.; do Nascimento, G.F.; Lakkadi, K.; Tura, A.; et al. A Mediterranean Diet and Low-Fat Vegan Diet to Improve Body Weight and Cardiometabolic Risk Factors: A Randomized, Cross-over Trial. *J Am Coll Nutr* **2021**, 1-13, https://doi.org/10.1080/07315724.2020.1869625

6. Jenkins, D.J.; Wong, J.M.; Kendall, C.W.; Esfahani, A.; Ng, V.W.; Leong, T.C.; Faulkner, D.A.; Vidgen, E.; Paul, G.; Mukherjea, R.; et al. Effect of a 6-month vegan low-carbohydrate ('Eco-Atkins') diet on cardiovascular risk factors and body weight in hyperlipidaemic adults: a randomised controlled trial. *BMJ Open* **2014**, *4*, e003505, https://doi.org/10.1136/bmjopen-2013-003505
7. Jenkins, D.J.; Wong, J.M.; Kendall, C.W.; Esfahani, A.; Ng, V.W.; Leong, T.C.; Faulkner, D.A.; Vidgen, E.; Greaves, K.A.; Paul, G.; et al. The effect of a plant-based low-carbohydrate ("Eco-Atkins") diet on body weight and blood lipid concentrations in hyperlipidemic subjects. *Archives of Internal Medicine* **2009**, *169*, 1046-1054, https://doi.org/10.1001/archinternmed.2009.115
8. Kahleova, H.; McCann, J.; Alwarith, J.; Rembert, E.; Tura, A.; Holubkov, R.; Barnard, N.D. A plant-based diet in overweight adults in a 16-week randomized clinical trial: The role of dietary acid load. *Clin Nutr ESPEN* **2021**, *44*, 150-158, https://doi.org/10.1016/j.clnesp.2021.05.015
9. Kahleova, H.; Petersen, K.F.; Shulman, G.I.; Alwarith, J.; Rembert, E.; Tura, A.; Hill, M.; Holubkov, R.; Barnard, N.D. Effect of a Low-Fat Vegan Diet on Body Weight, Insulin Sensitivity, Postprandial Metabolism, and Intramyocellular and Hepatocellular Lipid Levels in Overweight Adults: A Randomized Clinical Trial. *JAMA Network Open* **2020**, *3*, e2025454, https://dx.doi.org/10.1001/jamanetworkopen.2020.25454.
10. Kahleova, H. Changes in Food and Nutrient Intake and Diet Quality on a Low-Fat Vegan Diet Are Associated with Changes in Body Weight and Body Composition in Overweight Adults: a Randomized Clinical Trial. *Diabetes* **2022**, *71*, https://doi.org/10.2337/db22-552-P
11. Kahleova, H.; Brennan, H.; Znayenko-Miller, T.; Holubkov, R.; Barnard, N.D. Does diet quality matter? A secondary analysis of a randomized clinical trial. *European Journal of Clinical Nutrition* **2023**, *28*, 28, https://dx.doi.org/10.1038/s41430-023-01371-y.
12. Kahleova, H.; Rembert, E.; Alwarith, J.; Yonas, W.N.; Tura, A.; Holubkov, R.; Agnello, M.; Chutkan, R.; Barnard, N.D. Effects of a Low-Fat Vegan Diet on Gut Microbiota in Overweight Individuals and Relationships with Body Weight, Body Composition, and Insulin Sensitivity. A Randomized Clinical Trial. *Nutrients* **2020**, *12*, https://doi.org/10.3390/nu12102917
13. Crosby, L.; Rembert, E.; Levin, S.; Green, A.; Ali, Z.; Jardine, M.; Nguyen, M.; Elliott, P.; Goldstein, D.; Freeman, A.; et al. Changes in Food and Nutrient Intake and Diet Quality on a Low-Fat Vegan Diet Are Associated with Changes in Body Weight, Body Composition, and Insulin Sensitivity in Overweight Adults: A Randomized Clinical Trial. *Journal of the Academy of Nutrition & Dietetics* **2022**, *122*, 1922-1939.e1920, https://dx.doi.org/10.1016/j.jand.2022.04.008.
14. Kahleova, H.; Dort, S.; Holubkov, R.; Barnard, N.D. A Plant-Based High-Carbohydrate, Low-Fat Diet in Overweight Individuals in a 16-Week Randomized Clinical Trial: The Role of Carbohydrates. *Nutrients* **2018**, *10*, 14, https://dx.doi.org/10.3390/nu10091302.
15. Kahleova, H.; Fleeman, R.; Hlozkova, A.; Holubkov, R.; Barnard, N.D. A plant-based diet in overweight individuals in a 16-week randomized clinical trial: metabolic benefits of plant protein. *Nutrition & Diabetes* **2018**, *8*, 58, https://dx.doi.org/10.1038/s41387-018-0067-4.
16. Kahleova, H.; Hlozkova, A.; Fleeman, R.; Fletcher, K.; Holubkov, R.; Barnard, N.D. Fat Quantity and Quality, as Part of a Low-Fat, Vegan Diet, Are Associated with Changes in Body Composition, Insulin Resistance, and Insulin Secretion. A 16-Week Randomized Controlled Trial. *Nutrients* **2019**, *11*, 13, https://dx.doi.org/10.3390/nu11030615.
17. Neacsu, M.; Fyfe, C.; Horgan, G.; Johnstone, A.M. Appetite control and biomarkers of satiety with vegetarian (soy) and meat-based high-protein diets for weight loss in obese men: a randomized crossover trial. *American Journal of Clinical Nutrition* **2014**, *100*, 548-558, https://dx.doi.org/10.3945/ajcn.113.077503.
18. Li, J.; Armstrong, C.L.; Campbell, W.W. Effects of Dietary Protein Source and Quantity during Weight Loss on Appetite, Energy Expenditure, and Cardio-Metabolic Responses. *Nutrients* **2016**, *8*, 63, https://dx.doi.org/10.3390/nu8020063.
19. Mahon, A.K.; Flynn, M.G.; Stewart, L.K.; McFarlin, B.K.; Iglay, H.B.; Mattes, R.D.; Lyle, R.M.; Considine, R.V.; Campbell, W.W. Protein intake during energy restriction: effects on body composition and markers of metabolic and cardiovascular health in postmenopausal women. *Journal of the American College of Nutrition* **2007**, *26*, 182-189, https://doi.org/10.1080/07315724.2007.10719600

20. Sofi, F.; Dinu, M.; Pagliai, G.; Cesari, F.; Gori, A.M.; Sereni, A.; Becatti, M.; Fiorillo, C.; Marcucci, R.; Casini, A. Low-Calorie Vegetarian Versus Mediterranean Diets for Reducing Body Weight and Improving Cardiovascular Risk Profile: CARDIVEG Study (Cardiovascular Prevention With Vegetarian Diet). *Circulation* **2018**, *137*, 1103-1113, https://dx.doi.org/10.1161/CIRCULATIONAHA.117.030088.
21. Macknin, M.; Kong, T.; Weier, A.; Worley, S.; Tang, A.S.; Alkhouri, N.; Golubic, M. Plant-based, no-added-fat or American heart association diets: Impact on cardiovascular risk in obese children with hypercholesterolemia and their parents. *Journal of Pediatrics* **2015**, *166*, 953-959.e953, https://doi.org/10.1016/j.jpeds.2014.12.058
