# Supplementary material for: Health Effects of Plant-Based Diets in People with Overweight or Obesity: A Systematic Review and Meta-Analysis
Source: Nutrients. 2026 Jun 19;18(12):1987. doi: 10.3390/nu18121987 (PMC13304861; doi:10.3390/nu18121987)

Supplementary file S5: ANALYSES

Effect of plant-based diets versus control diet on body weight

Analysis S1.1 Plant-based diets versus control (omnivorous) diet. Outcome: body weight (kg)

See Figure 2 in manuscript.

Analysis S1.2 Plant-based diets versus control diet (lacto-ovo vegetarian diet). Outcome: body weight (kg)

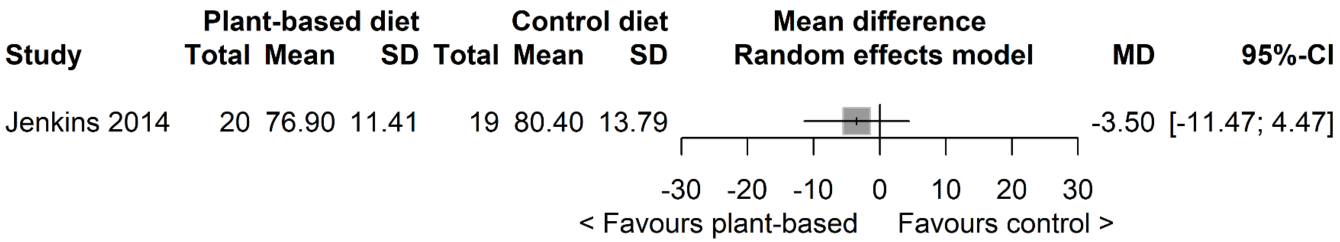

Analysis S1.3 Plant-based diets versus control (omnivorous) diet. Outcome: body weight (kg) by diet

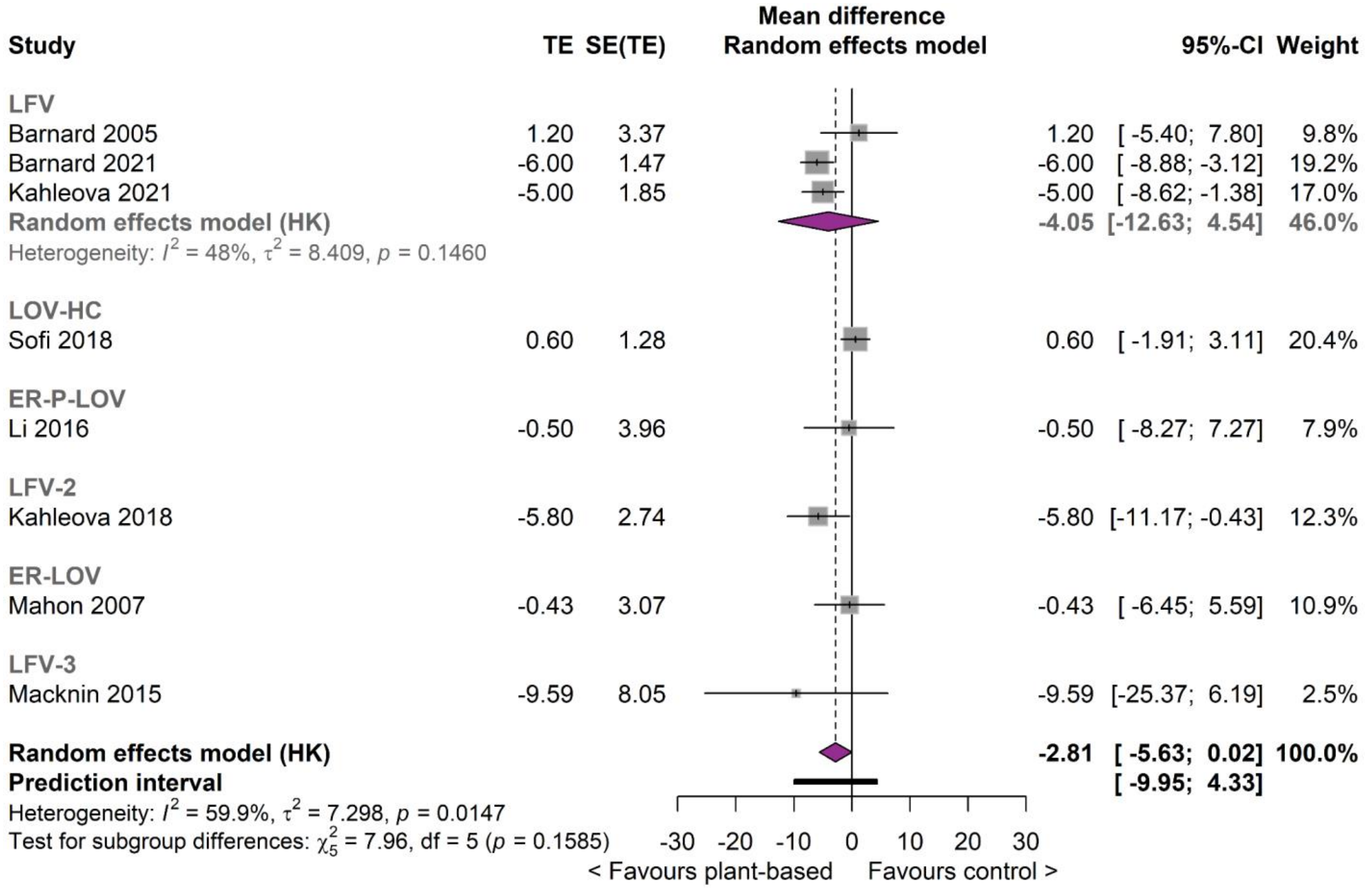

Analysis S1.4 Plant-based diets versus control (omnivorous) diet. Outcome: body weight (kg) by time

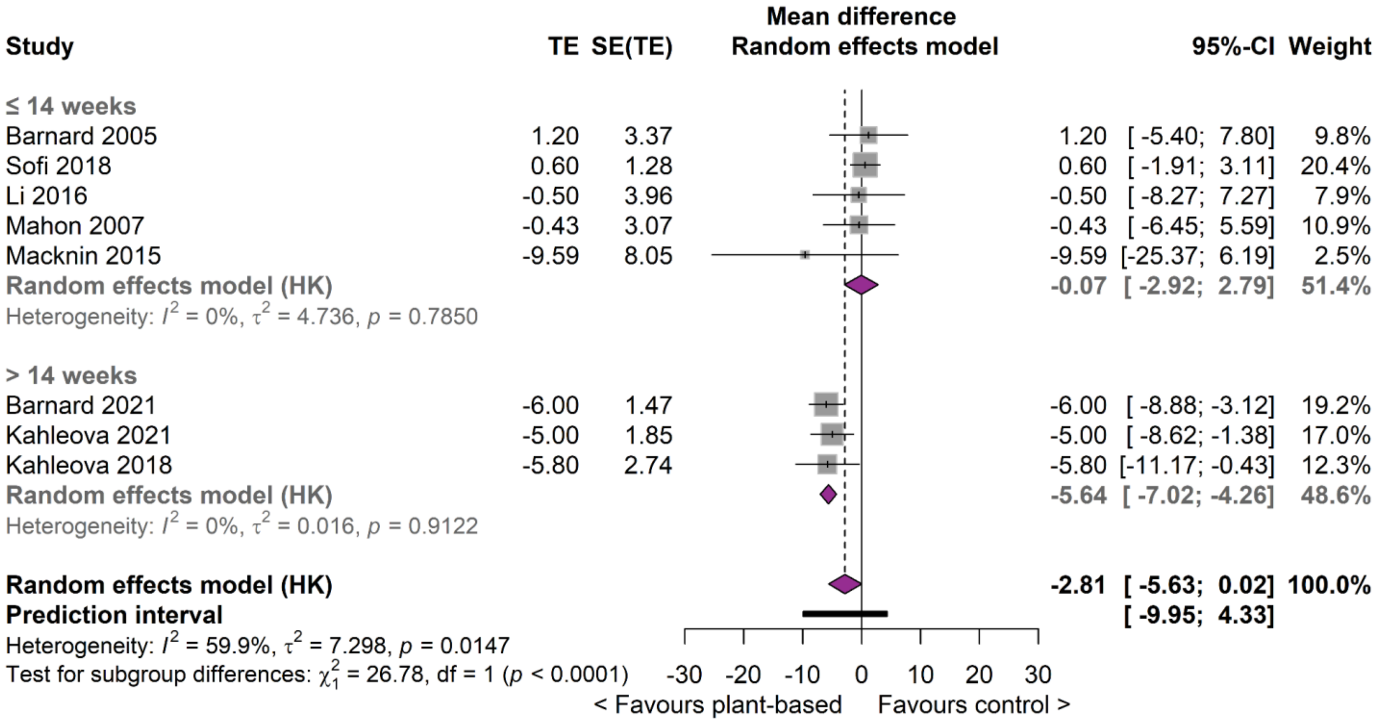

Effect of plant-based diets versus control diet on BMI

Analysis S2.1 Plant-based diets versus control (omnivorous) diet. Outcome: BMI (kg/m²)

See Figure 3 in manuscript

Analysis S2.2 Plant-based diet versus control diet (lacto-ovo vegetarian diet). Outcome: BMI (kg/m²)

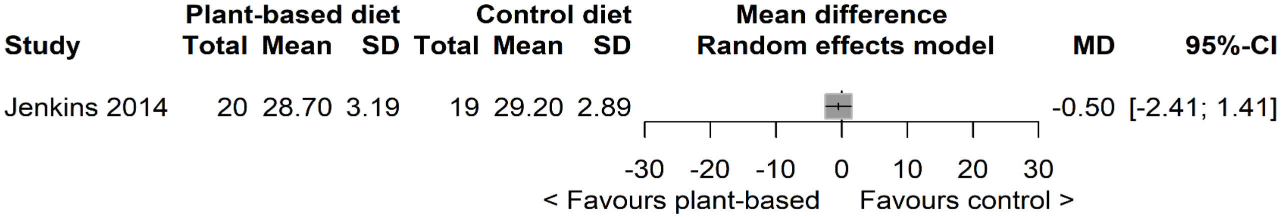

Analysis S2.3 Plant-based diet versus control (omnivorous) diet. Outcome: BMI percentile, BMI z-score (children)

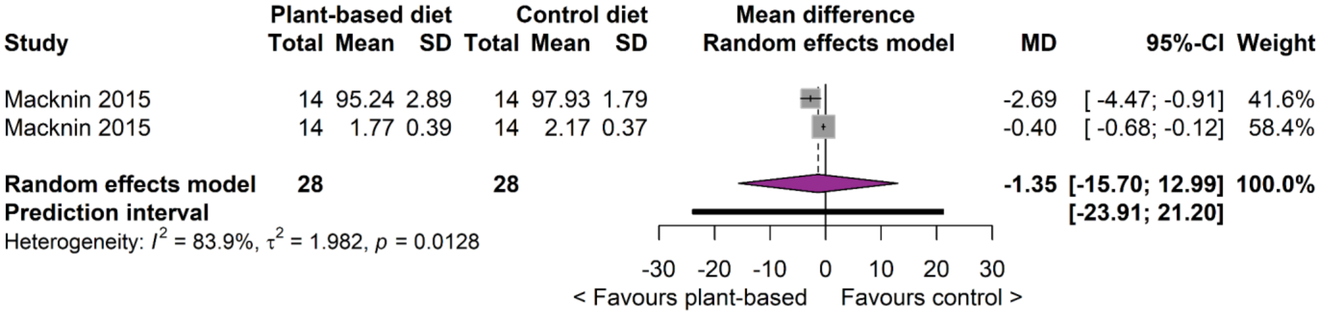

Analysis S2.4 Plant-based diets versus control (omnivorous) diet. Outcome: BMI (kg/m²) by diet

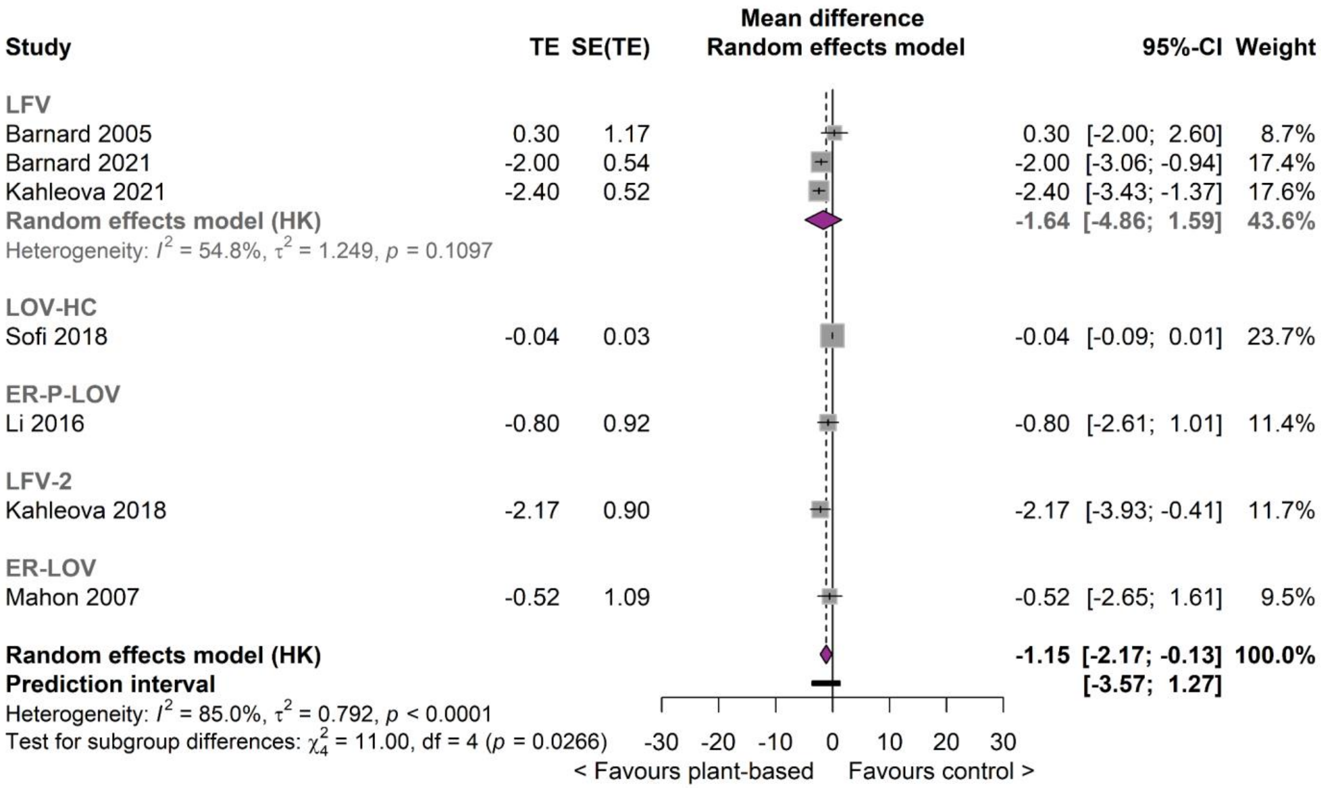

Analysis S2.5 Plant-based diets versus control (omnivorous) diet. Outcome: BMI (kg/m2) by time

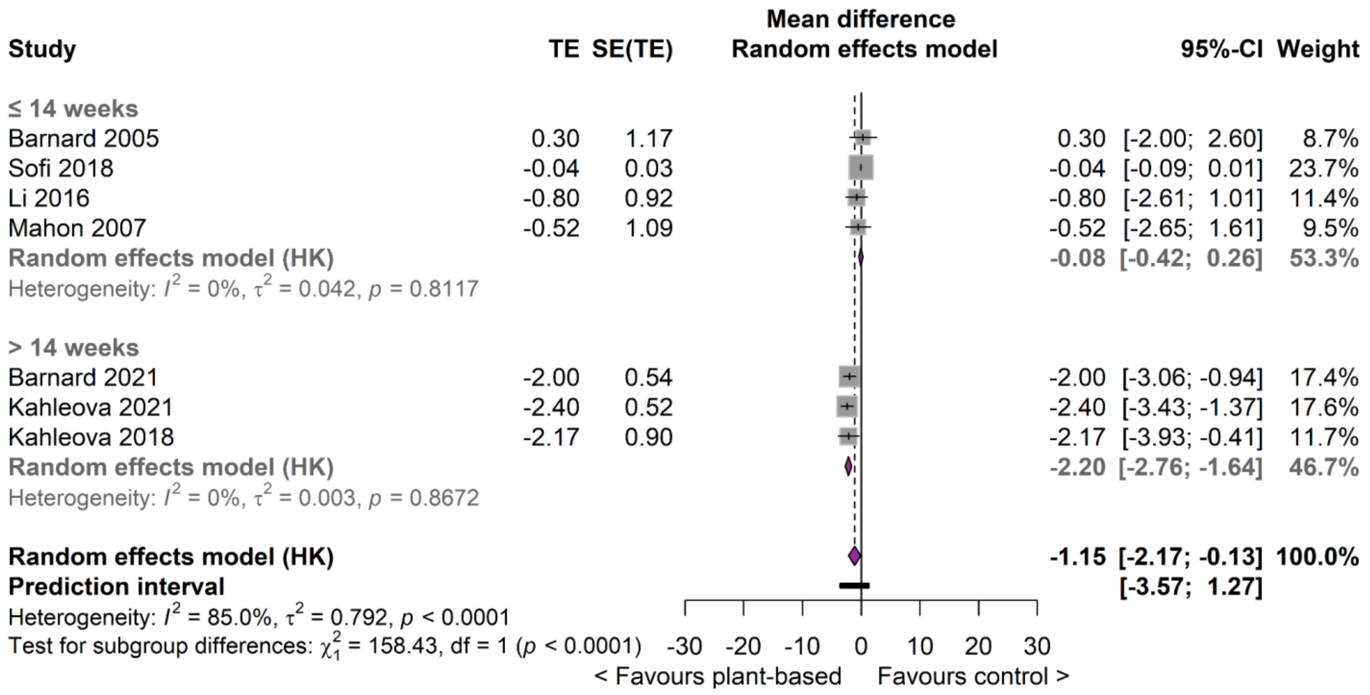

Effect of plant-based diets versus control diet on body fat mass

Analysis S3.1 Plant-based diets versus control (omnivorous) diet. Outcome: body fat mass (in kg)

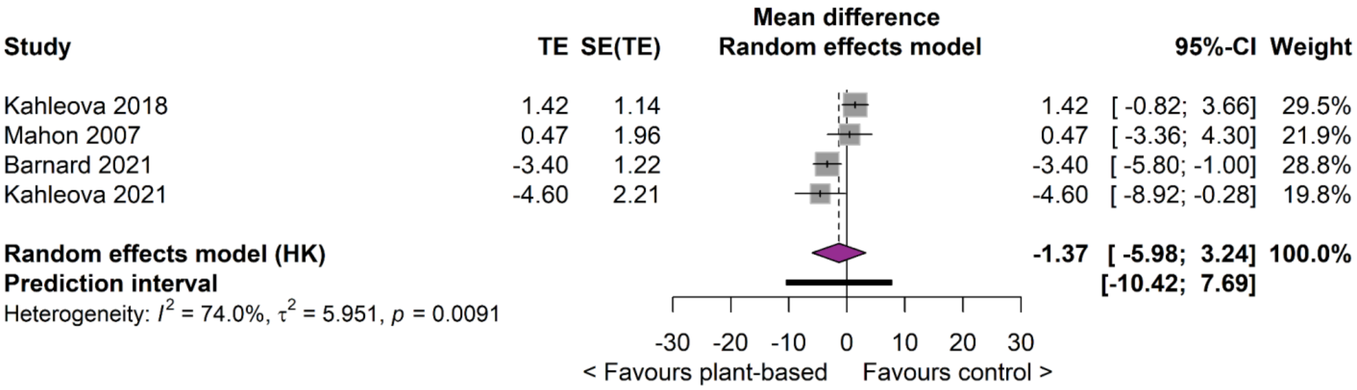

Analysis S3.2 Plant-based diets versus control (omnivorous) diet. Outcome: body fat mass (in %)

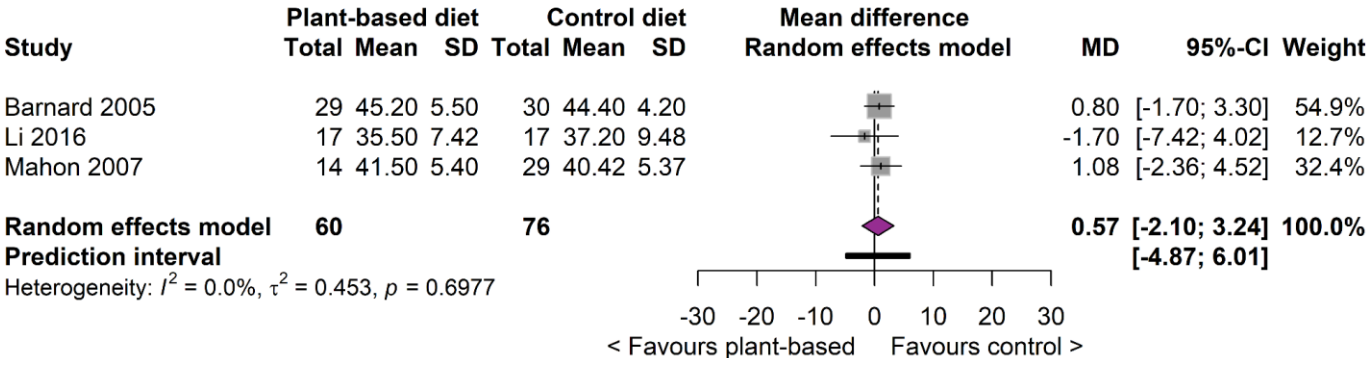

Analysis S3.3 Plant-based diets versus control (omnivorous) diet. Outcome: body fat mass (combined)

Study	TE	SE(TE)	Mean difference Random effects model	95%-CI	Weight
Barnard 2005	0.80	1.03		0.80 [-1.22; 2.82]	21.2%
Li 2016	-1.70	1.99		-1.70 [-5.60; 2.20]	13.4%
Kahleova 2018	1.42	1.14		1.42 [-0.82; 3.66]	20.2%
Mahon 2007	0.47	1.96		0.47 [-3.36; 4.30]	13.7%
Barnard 2021	-3.40	1.22		-3.40 [-5.80; -1.00]	19.5%
Kahleova 2021	-4.60	2.21		-4.60 [-8.92; -0.28]	12.1%
**Random effects model (HK)**				**-0.93 [-3.47; 1.62]**	**100.0%**
**Prediction interval**				**[-6.70; 4.85]**	

Heterogeneity: I^2 = 64.5%, τ^2 = 3.973, p = 0.0151

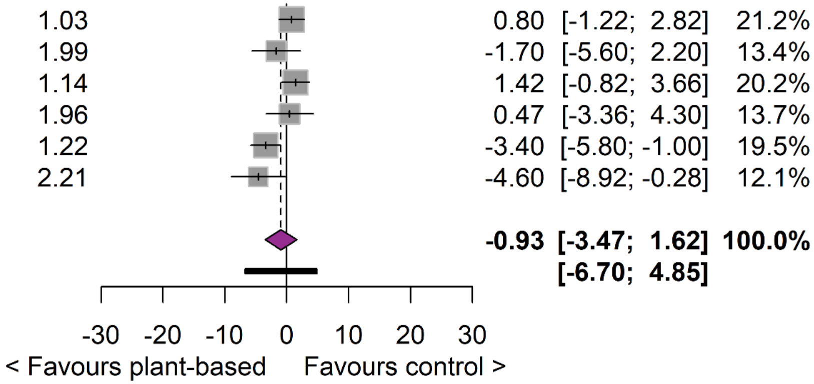

Analysis S3.4 Plant-based diet versus control diet (lacto-ovo vegetarian diet). Outcome: body fat mass (%)

Study	Plant-based diet Total	Mean	SD	Control diet Total	Mean	SD	Mean difference Random effects model	MD	95%-CI
Jenkins 2014	20	31.40	11.98	19	35.00	9.45		-3.60	[-10.35; 3.15]

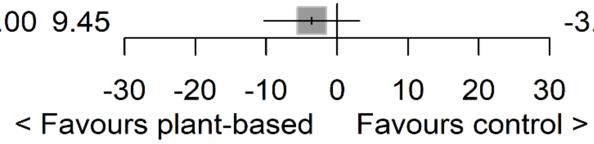

Analysis S3.5 Plant-based diet versus control (omnivorous) diet. Outcome: body fat mass (kg) by diet

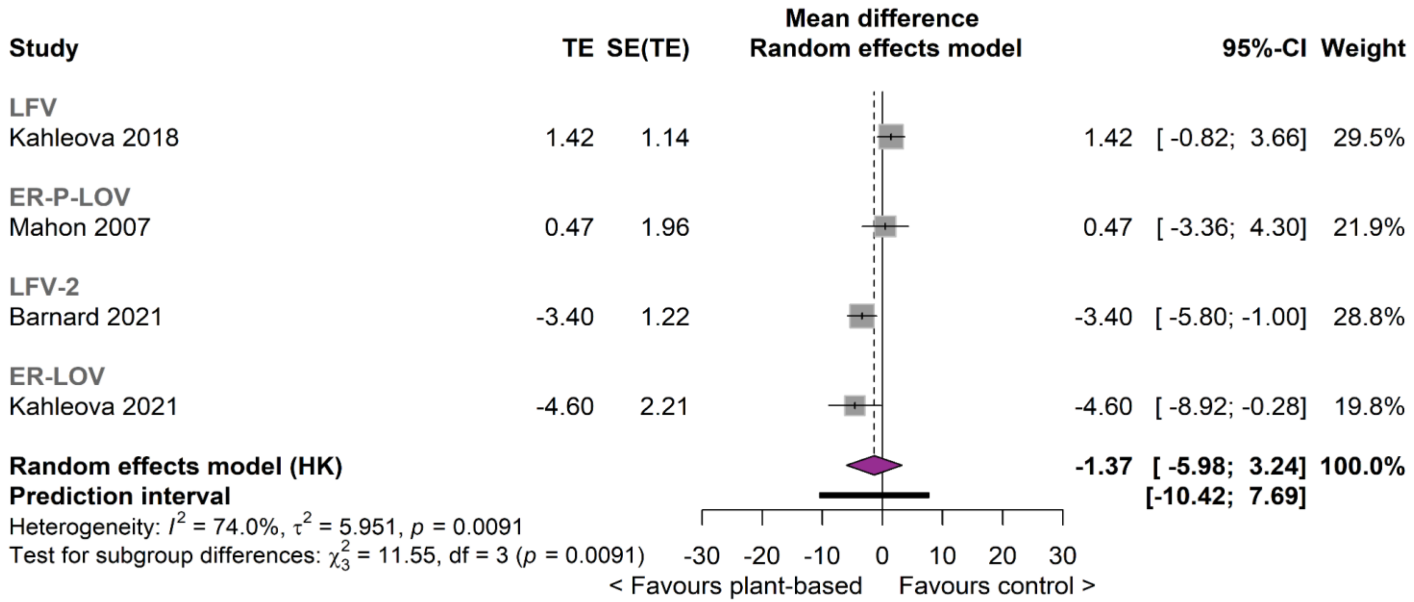

Analysis S3.6 Plant-based diets versus control (omnivorous) diet. Outcome: body fat mass (%) by diet

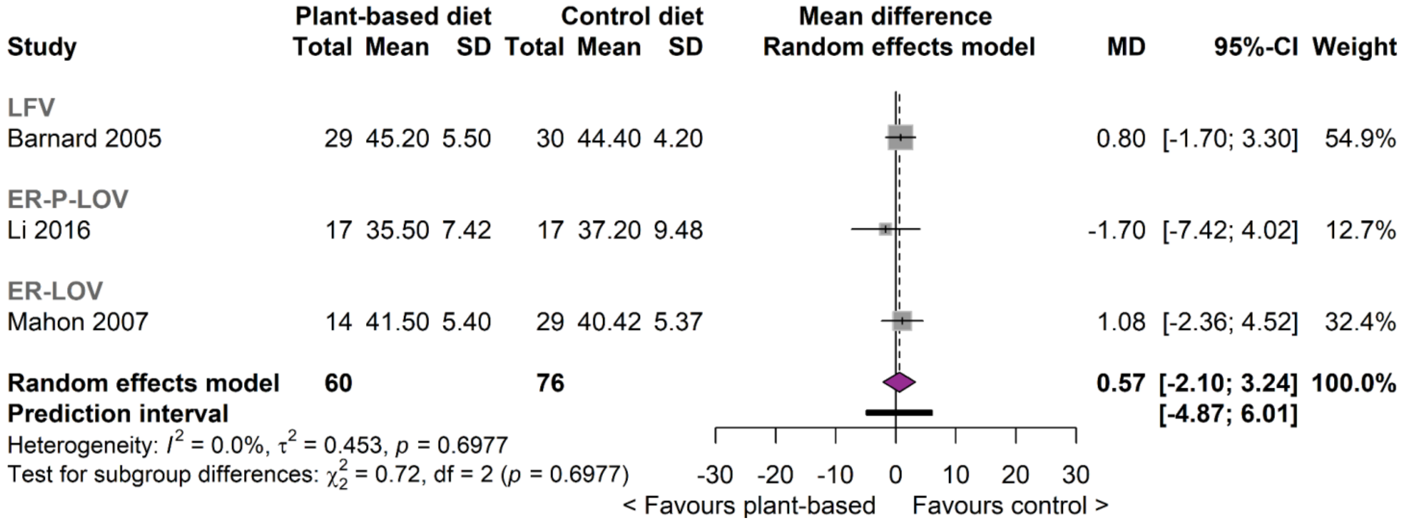

Analysis S3.7 Plant-based diets versus control (omnivorous) diet. Outcome: body fat mass (combined) by diet

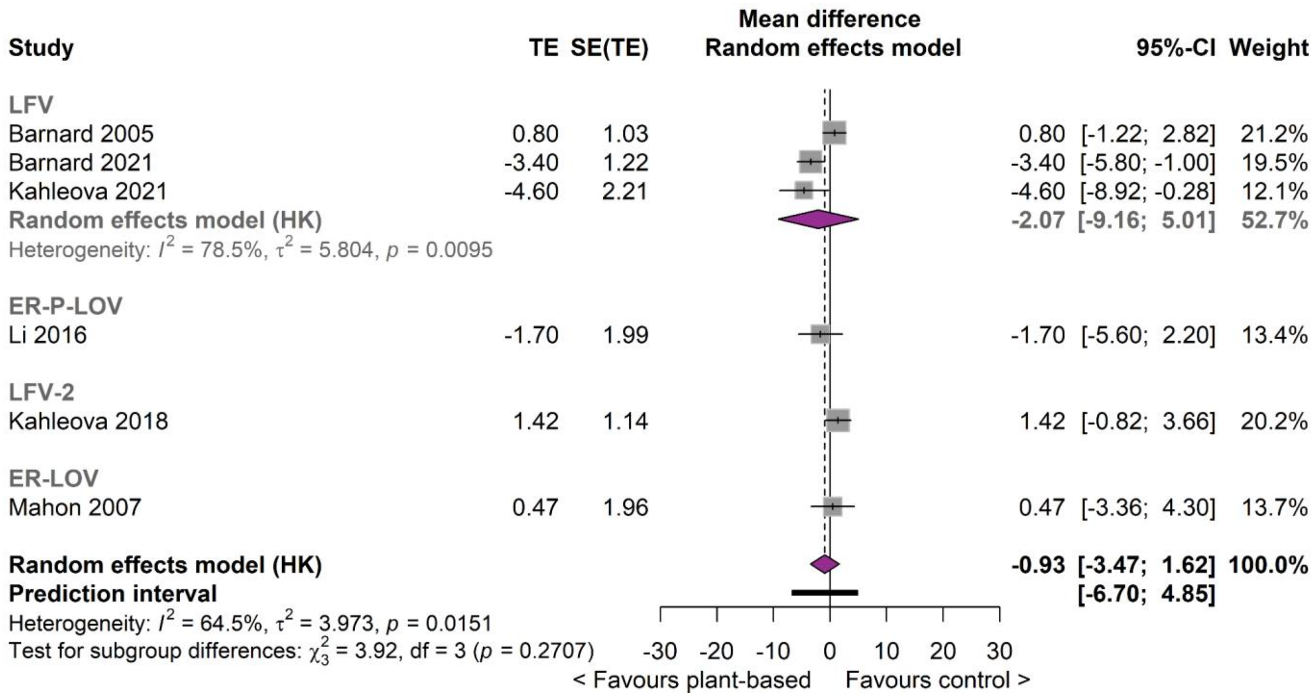

Analysis S3.8 Plant-based diets versus control (omnivorous) diet. Outcome: body fat mass (kg) by time

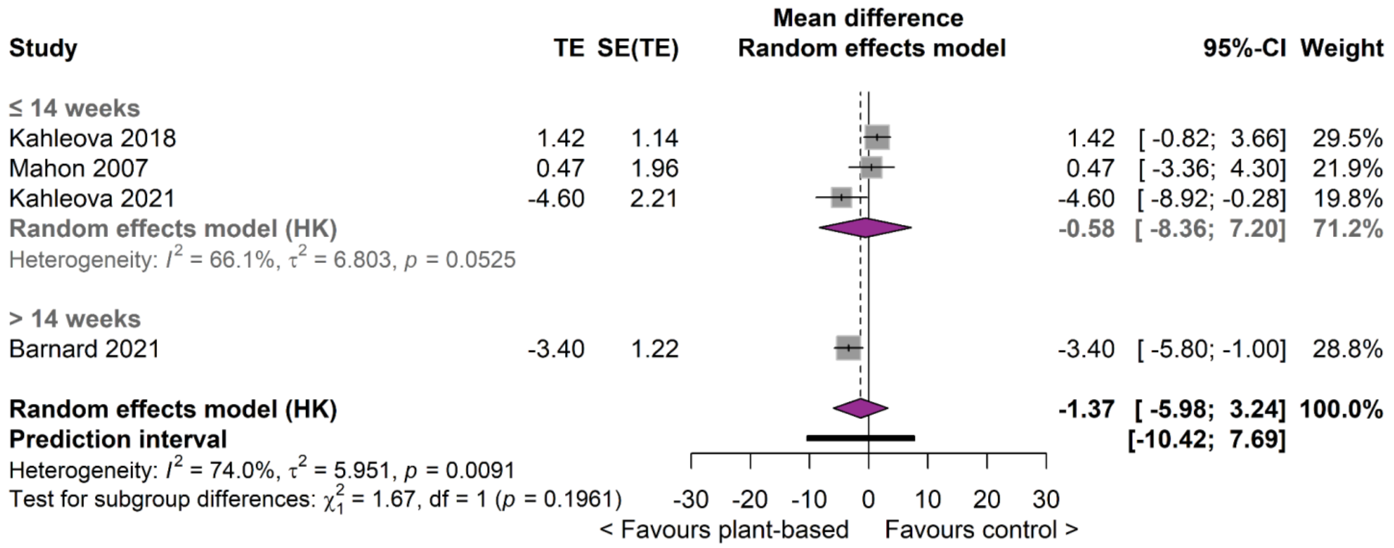

Analysis S3.9 Plant-based diets versus control (omnivorous) diet. Outcome: body fat mass (%) by time

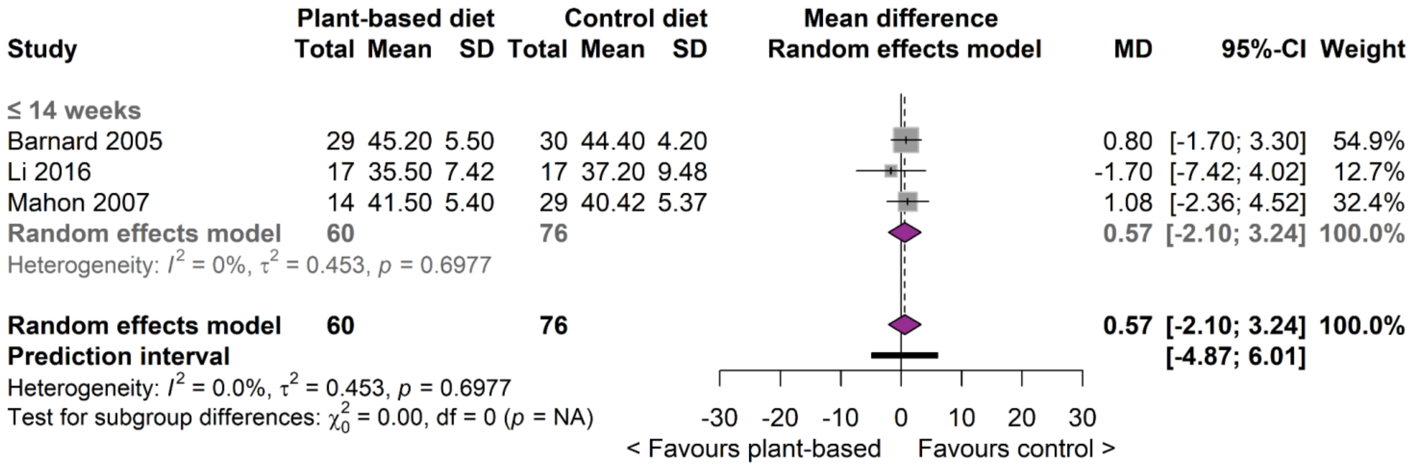

Analysis S3.10 Plant-based diets versus control (omnivorous) diet. Outcome: body fat mass (combined) by time

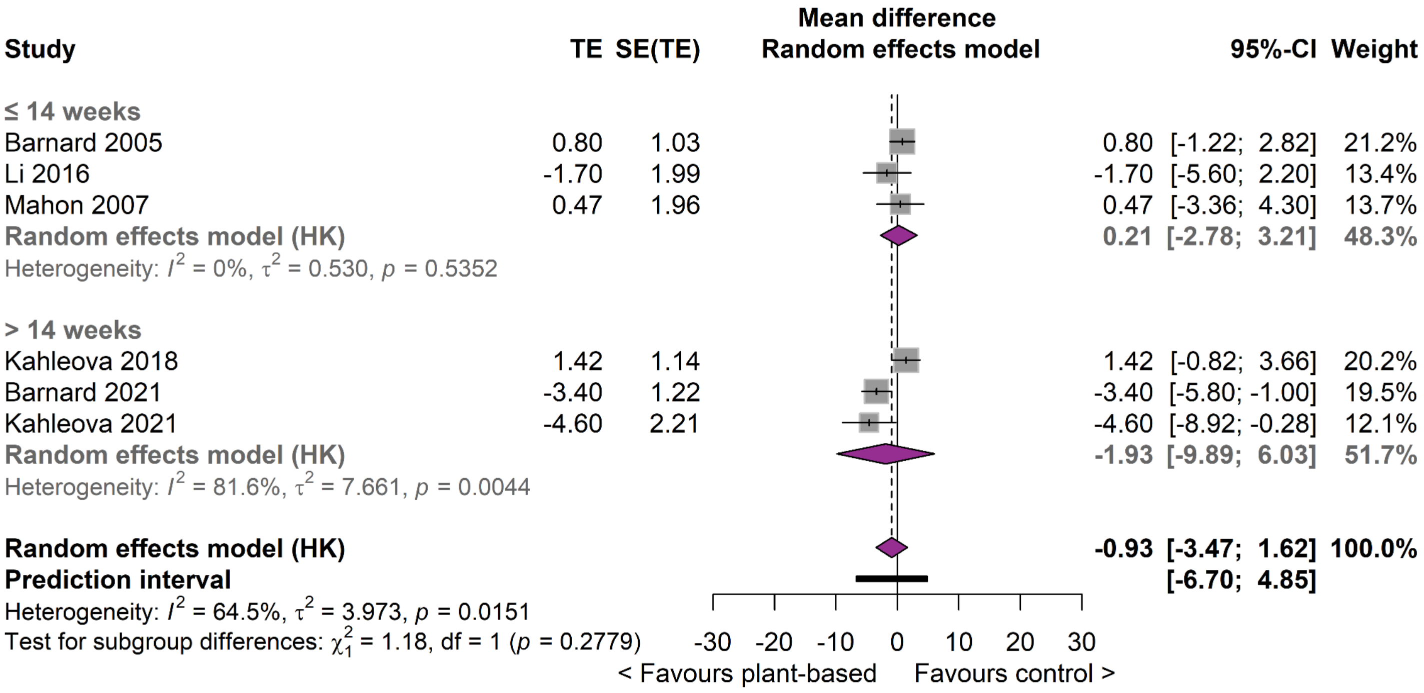

Effect of plant-based diets versus control diet on blood pressure

Analysis S4.1 Plant-based diets versus control (omnivorous) diet. Outcome: systolic blood pressure (mmHg)

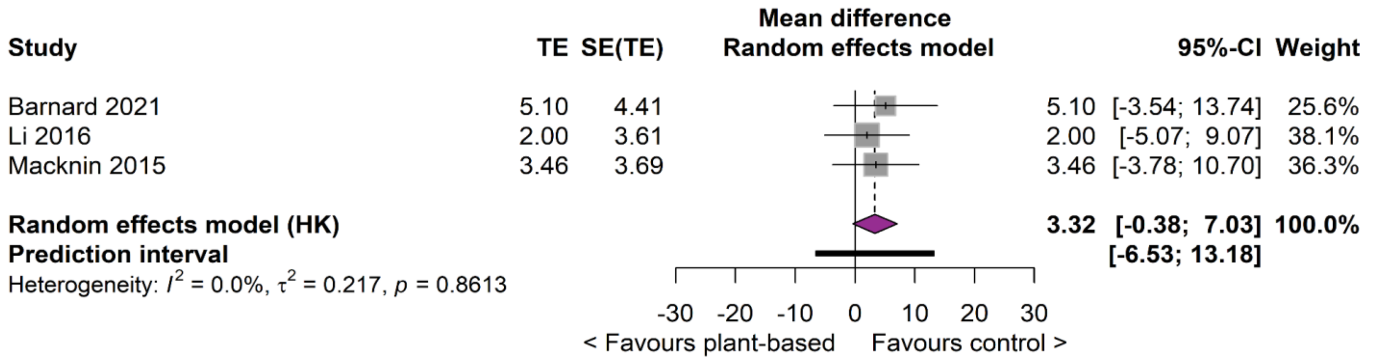

Analysis S4.2 Plant-based diets versus control (omnivorous) diet. Outcome: diastolic blood pressure (mmHg)

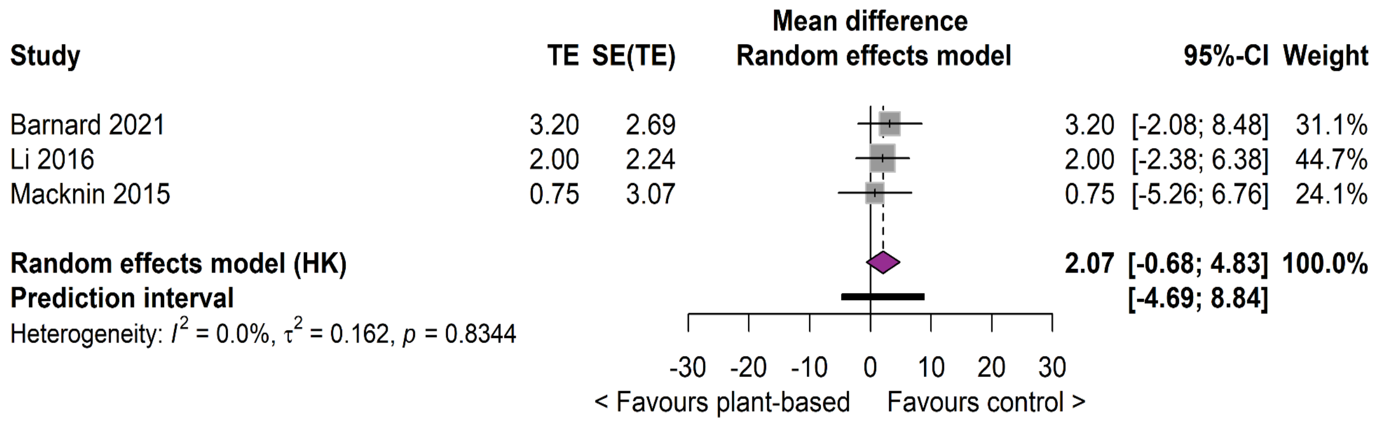

Analysis S4.3 Plant-based diets versus control diet (lacto-ovo vegetarian diet). Outcome: systolic blood pressure (mmHg)

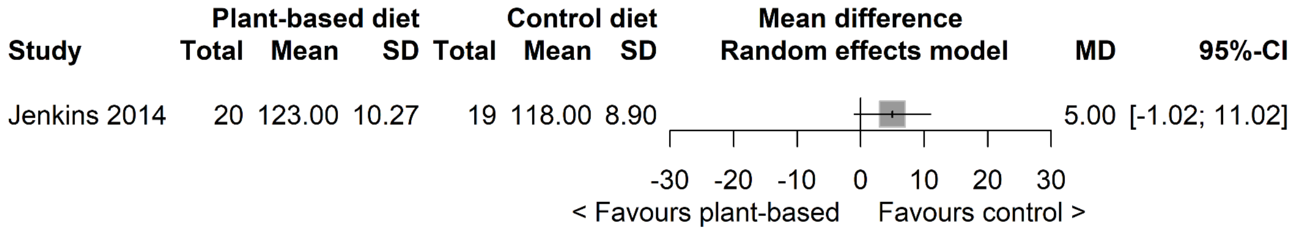

Analysis S4.4 Plant-based diet versus control diet (lacto-ovo vegetarian diet). Outcome: diastolic blood pressure (mmHg)

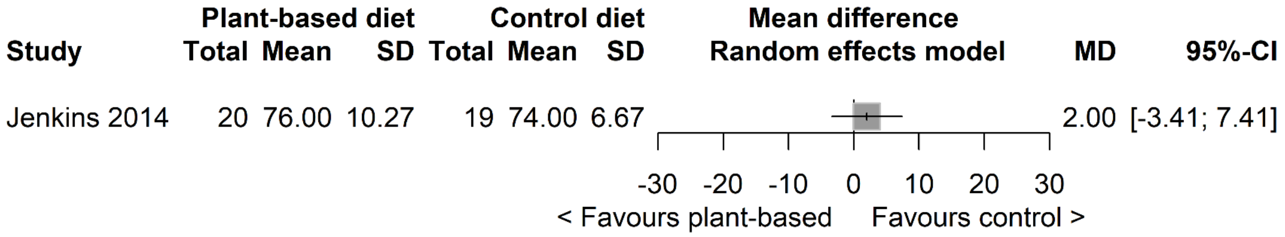

Analysis S4.5 Plant-based diets versus control (omnivorous) diet. Outcome: systolic blood pressure (mmHg) by diet

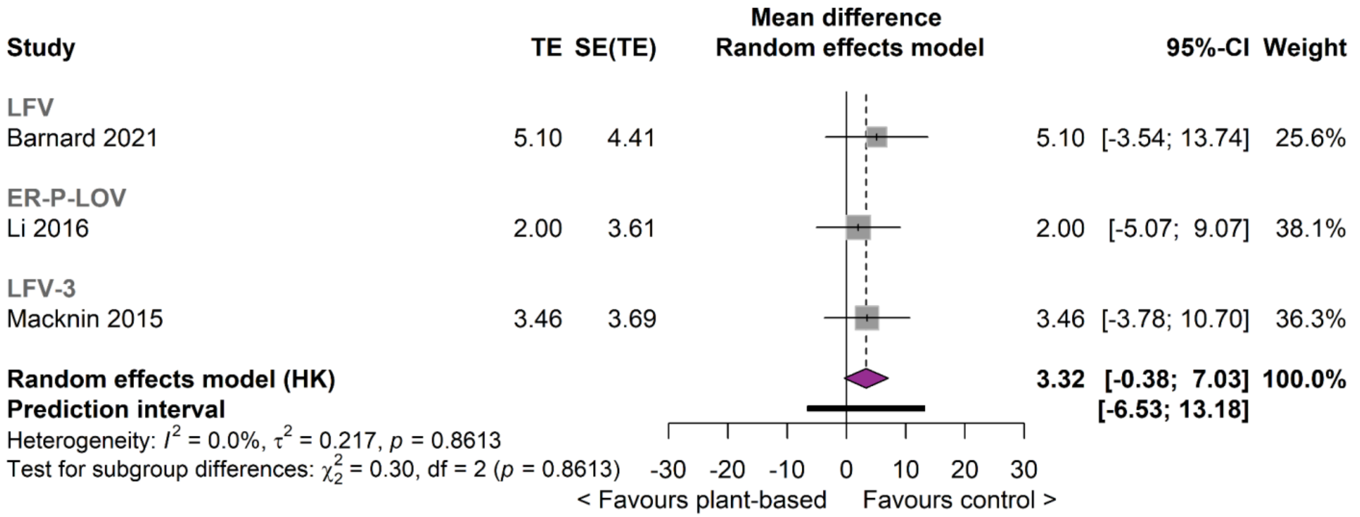

Analysis S4.6 Plant-based diets versus control (omnivorous) diet. Outcome: diastolic blood pressure (mmHg) by diet

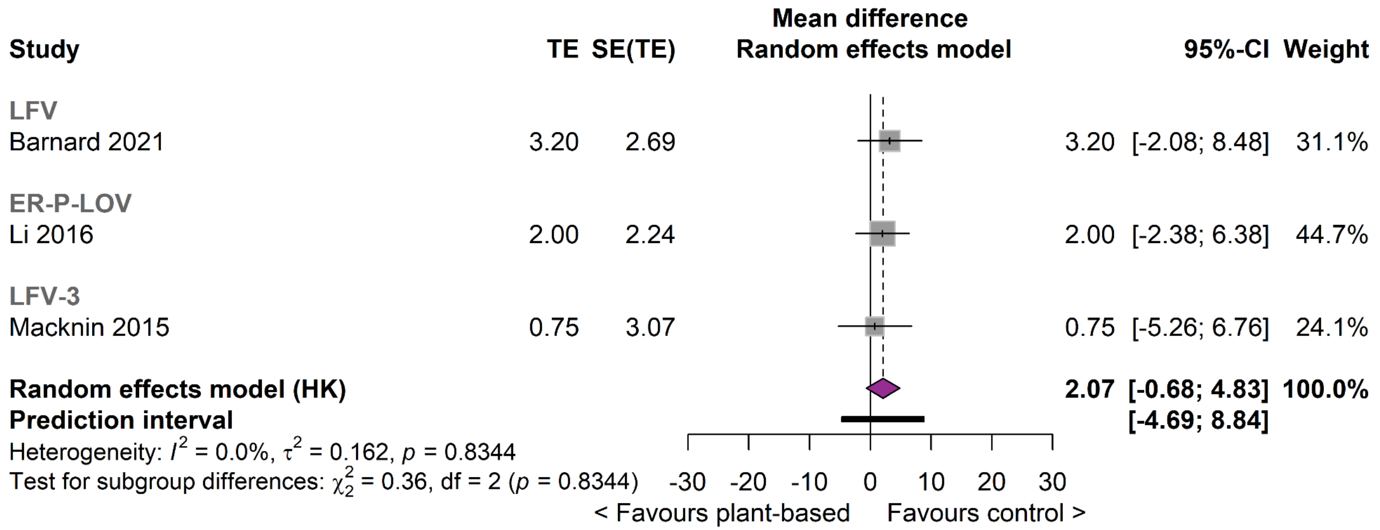

Analysis S4.7 Plant-based diets versus control (omnivorous) diet. Outcome: systolic blood pressure (mmHg) by time

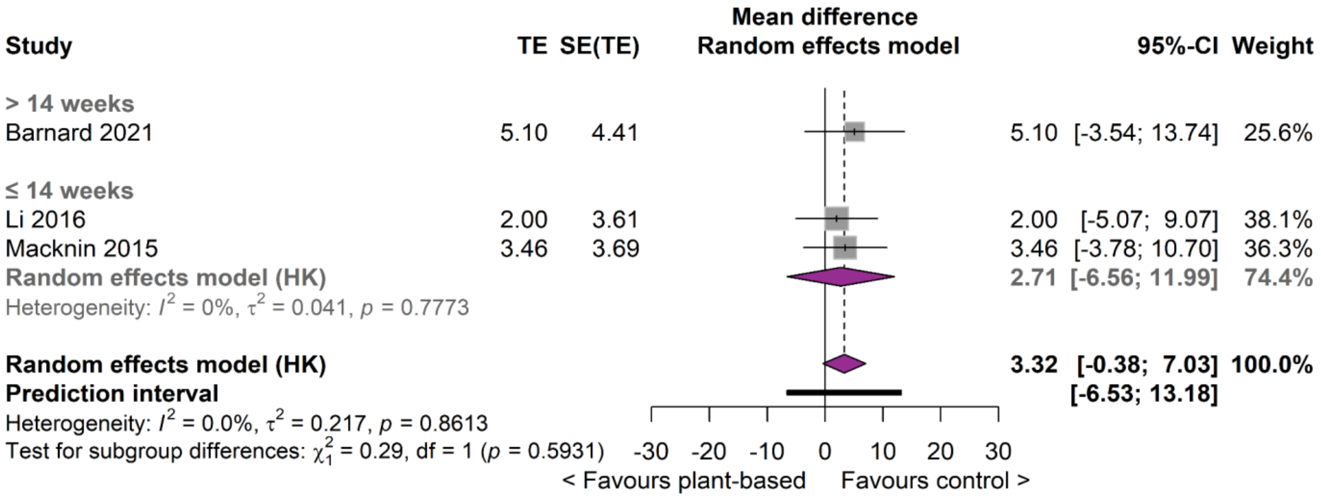

Analysis S4.8 Plant-based diets versus control (omnivorous) diet. Outcome: diastolic blood pressure (mmHg) by time

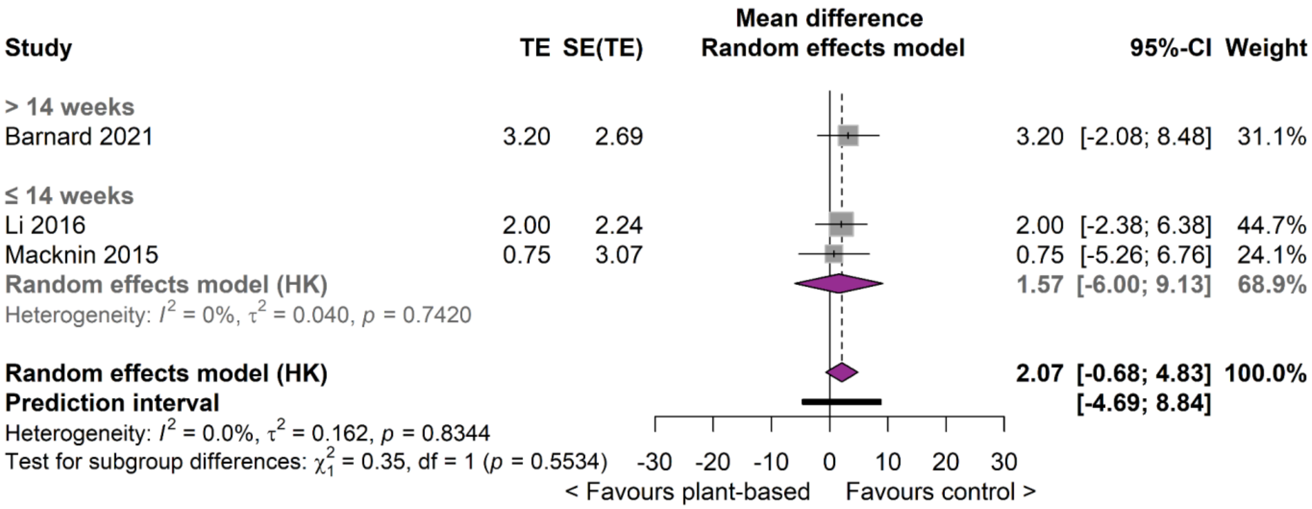

Effect of plant-based diets versus control diet on lipid levels

Analysis S5.1 Plant-based diets versus control (omnivorous) diet. Outcome: total cholesterol (mg/dl)

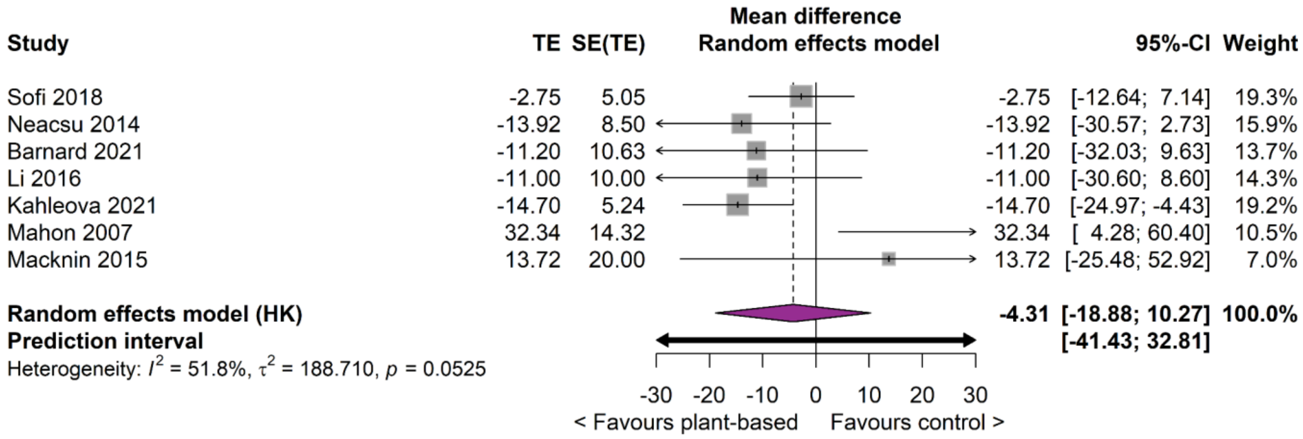

Analysis S5.2 Plant-based diet versus control diet (lacto-ovo vegetarian diet). Outcome: total cholesterol (mg/dl)

Study	Plant-based diet Total	Plant-based diet Mean	Plant-based diet SD	Control diet Total	Control diet Mean	Control diet SD	Mean difference Random effects model	MD	95%-CI
Jenkins 2014	20	235.89	37.90	19	250.97	45.24	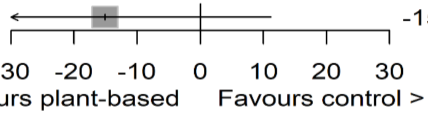	-15.08	[-41.34; 11.18]

Analysis S5.3 Plant-based diets versus control (omnivorous) diet. Outcome: total cholesterol (mg/dl) by diet

Study	TE	SE(TE)	Mean difference Random effects model	95%-CI	Weight
**LOV-HC**					
Sofi 2018	-2.75	5.05	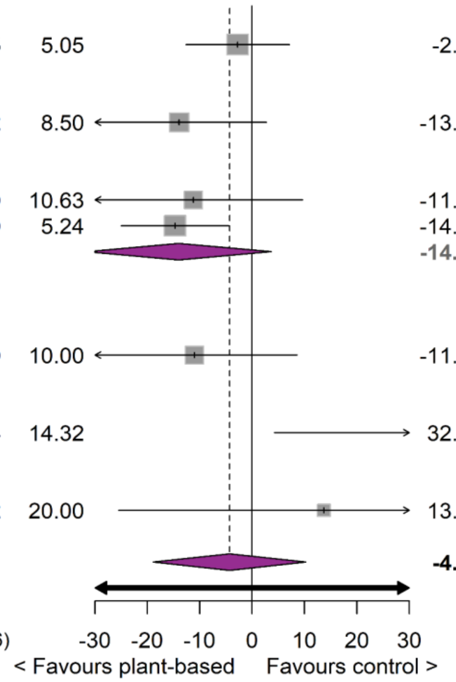	-2.75 [-12.64; 7.14]	19.3%
**Soy-HPWL**					
Neacsu 2014	-13.92	8.50		-13.92 [-30.57; 2.73]	15.9%
**LFV**					
Barnard 2021	-11.20	10.63		-11.20 [-32.03; 9.63]	13.7%
Kahleova 2021	-14.70	5.24		-14.70 [-24.97; -4.43]	19.2%
**Random effects model (HK)**				**-14.01 [-31.69; 3.67]**	**32.9%**
Heterogeneity: $I^2 = 0\%$, $\tau^2 = 0.256$, $p = 0.7677$					
**ER-P-LOV**					
Li 2016	-11.00	10.00		-11.00 [-30.60; 8.60]	14.3%
**ER-LOV**					
Mahon 2007	32.34	14.32		32.34 [4.28; 60.40]	10.5%
**LFV-3**					
Macknin 2015	13.72	20.00		13.72 [-25.48; 52.92]	7.0%
**Random effects model (HK)**				**-4.31 [-18.88; 10.27]**	**100.0%**
**Prediction interval**				**[-41.43; 32.81]**	
Heterogeneity: $I^2 = 51.8\%$, $\tau^2 = 188.710$, $p = 0.0525$					
Test for subgroup differences: $\chi^2_5 = 16.47$, df = 5 ($p = 0.0056$)					

Analysis S5.4 Plant-based diets versus control (omnivorous) diet. Outcome: total cholesterol (mg/dl) by time

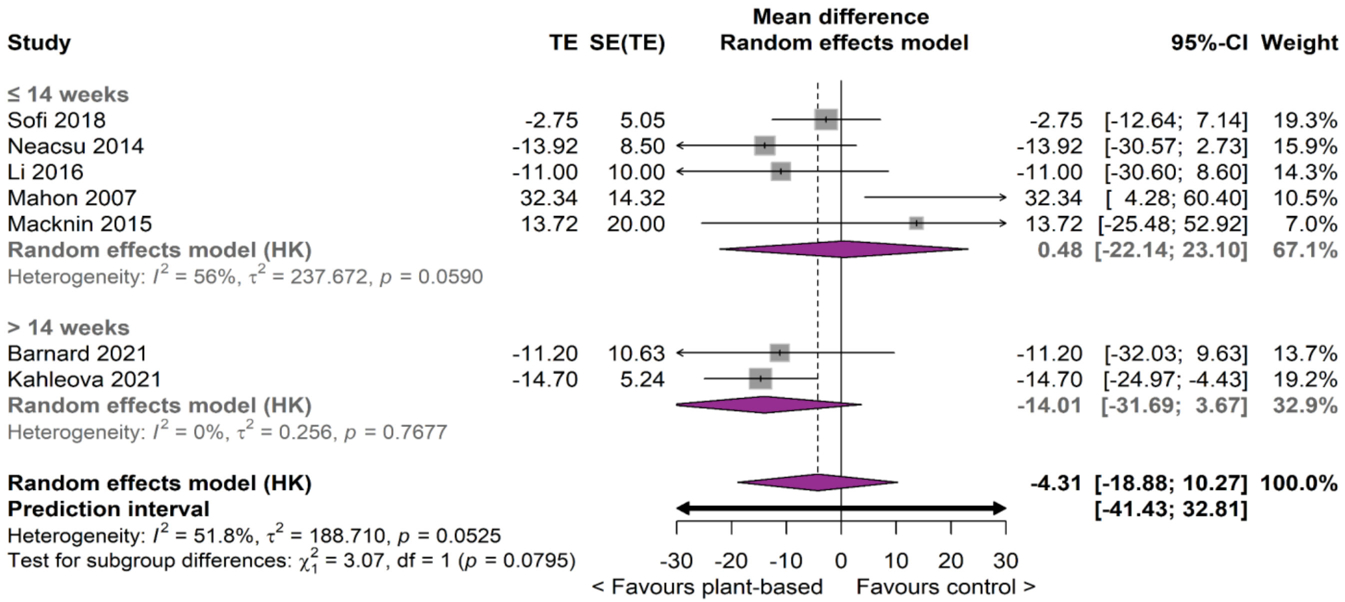

Analysis S5.5 Plant-based diets versus control (omnivorous) diet. Outcome: LDL-cholesterol (mg/dl)

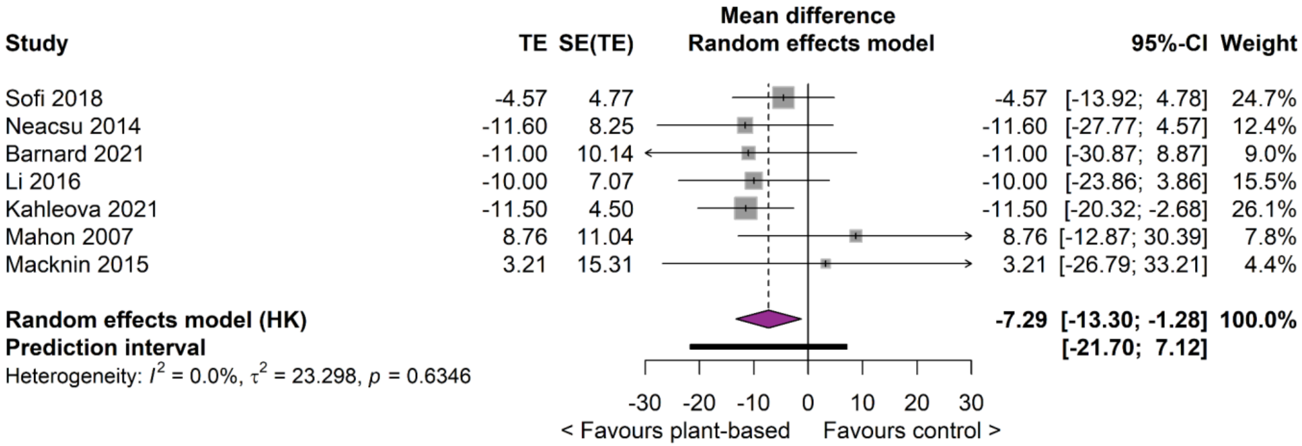

Analysis S5.6 Plant-based diet versus control diet (lacto-ovo vegetarian diet). Outcome: LDL-cholesterol (mg/dl)

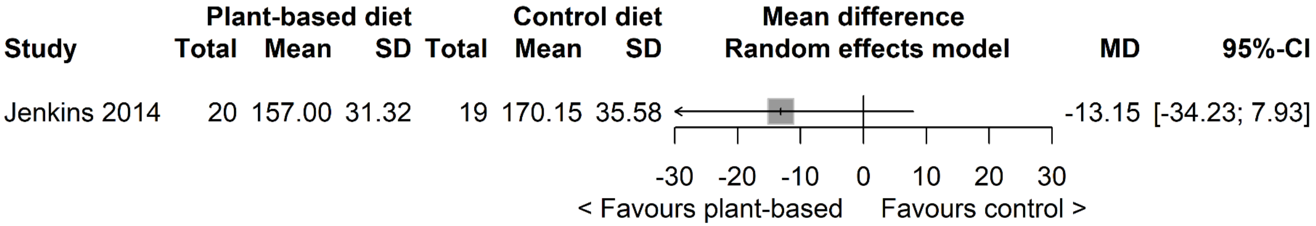

Analysis S5.7 Plant-based diets versus control (omnivorous) diet. Outcome: LDL-cholesterol (mg/dl) by diet

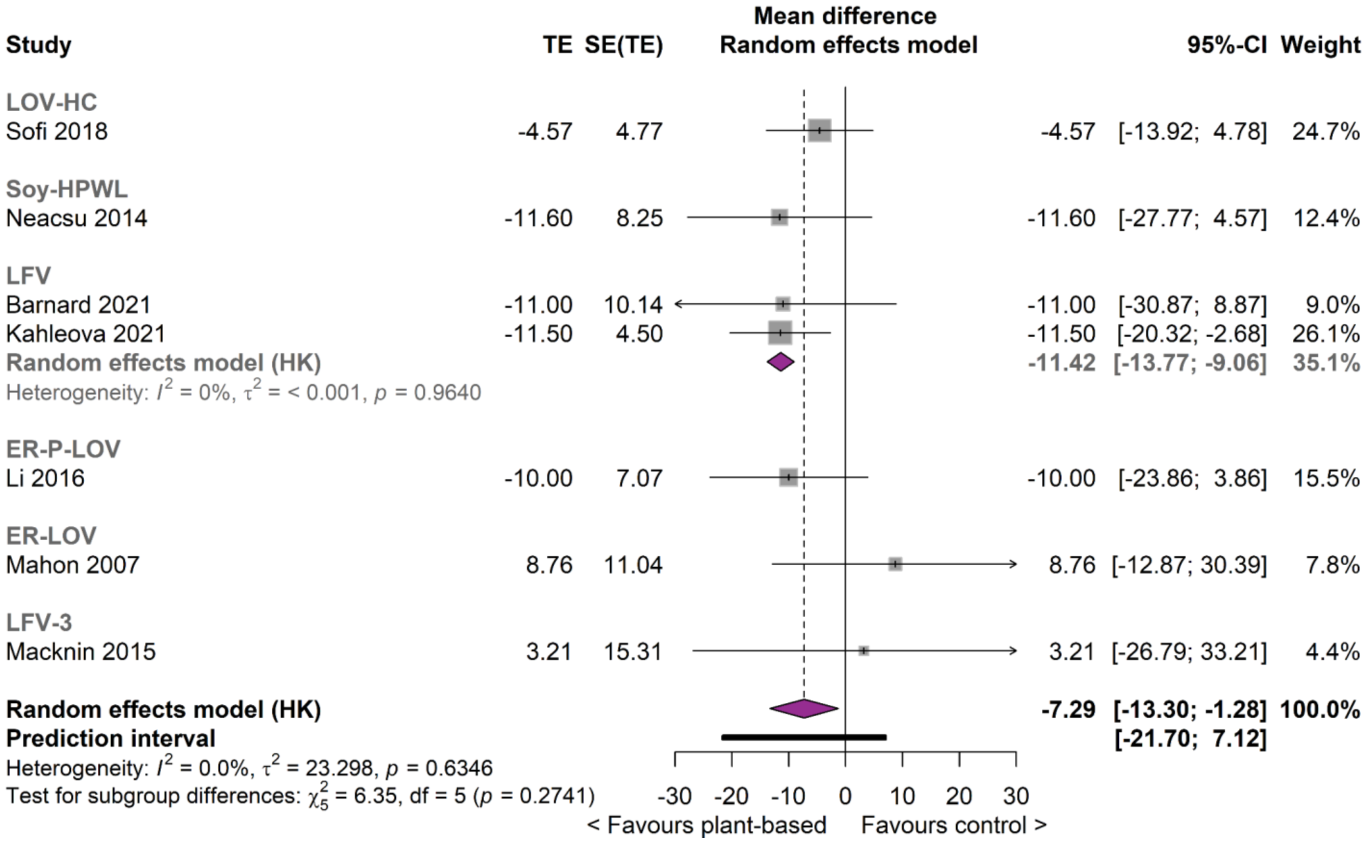

Analysis S5.8 Plant-based diets versus control (omnivorous) diet. Outcome: LDL-cholesterol (mg/dl) by time

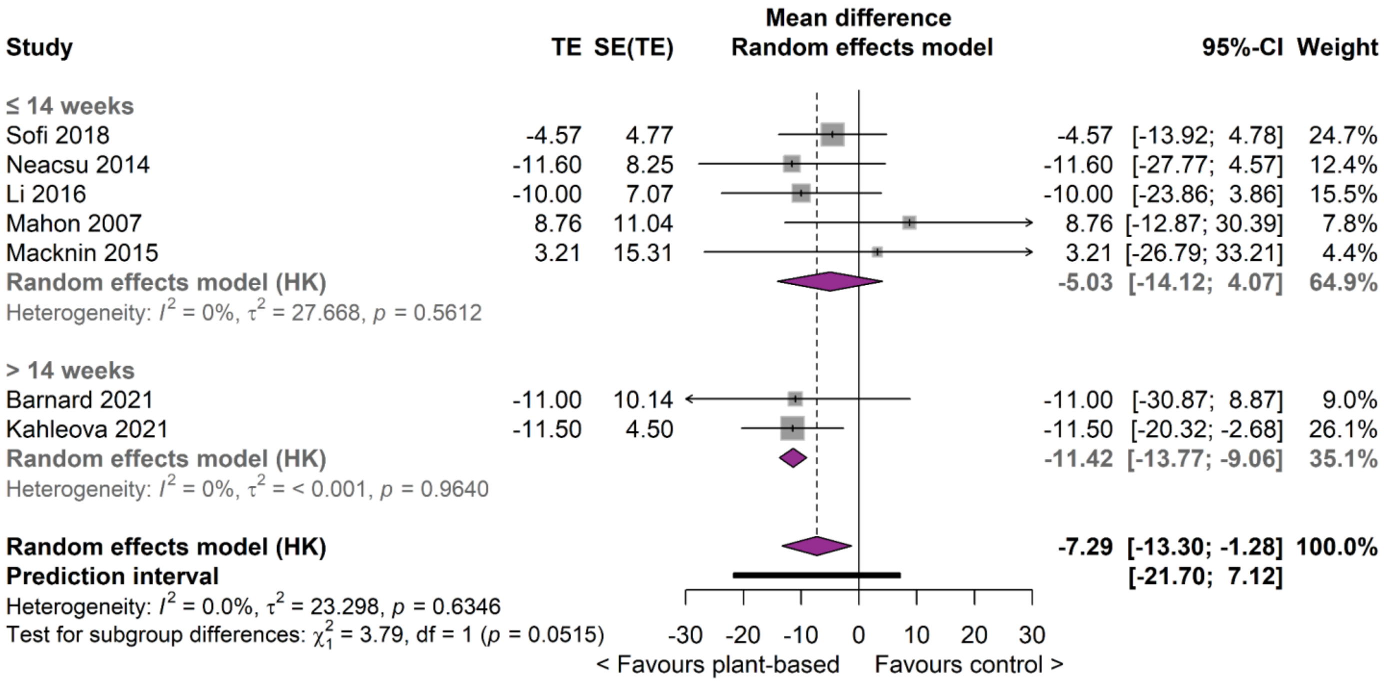

Analysis S5.9 Plant-based diets versus control (omnivorous) diet. Outcome: HDL-cholesterol (mg/dl)

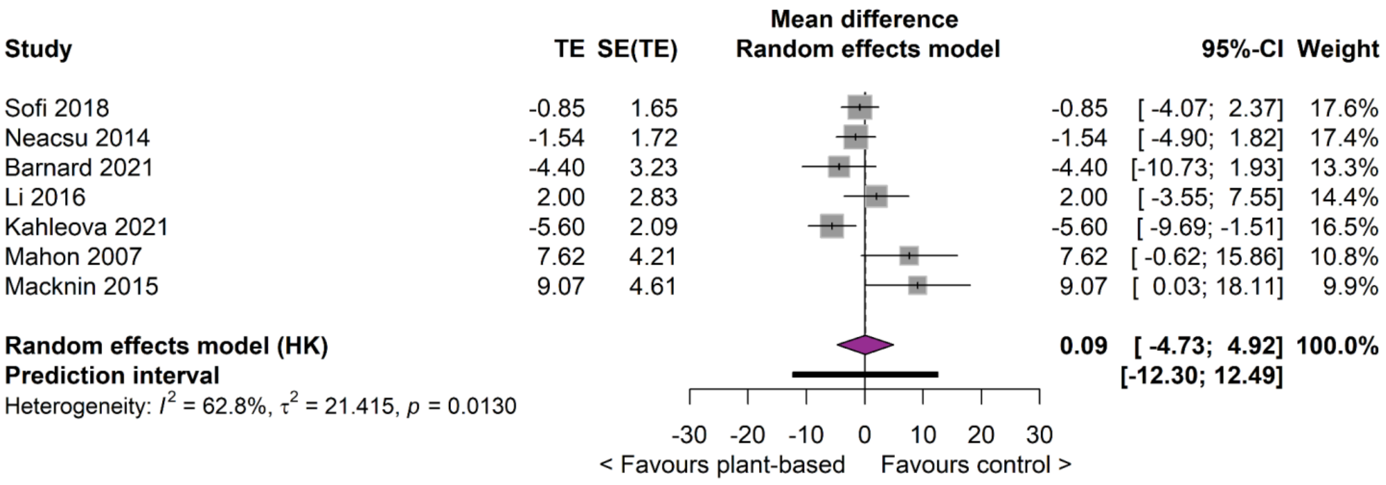

Analysis S5.10 Plant-based diet versus control diet (lacto-ovo vegetarian diet). Outcome: HDL-cholesterol (mg/dl)

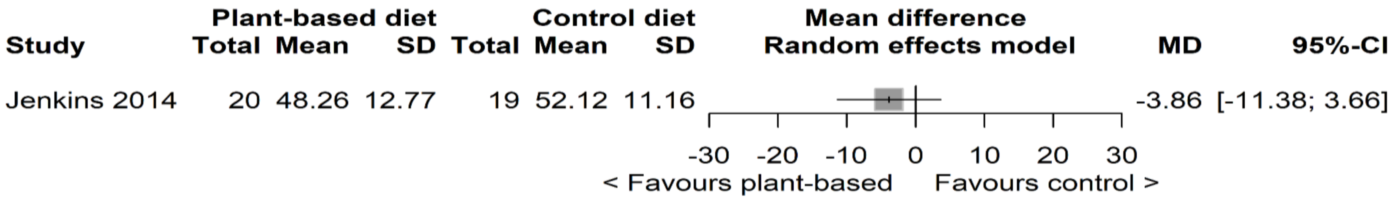

Analysis S5.11 Plant-based diets versus control (omnivorous) diet. Outcome: HDL-cholesterol (mg/dl) by time

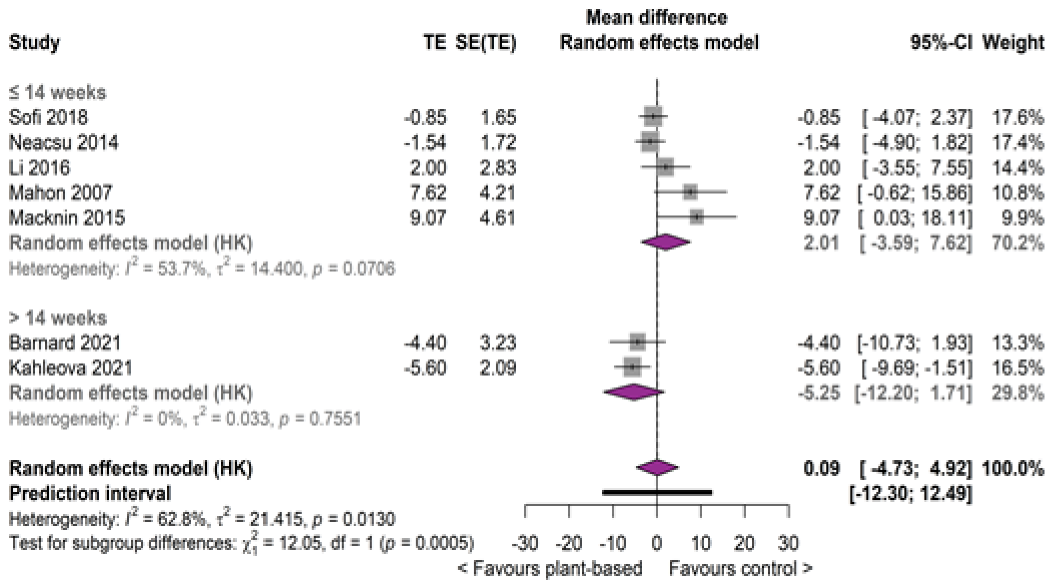

Analysis S5.12 Plant-based diets versus control (omnivorous) diet. Outcome: HDL-cholesterol (mg/dl) by diet

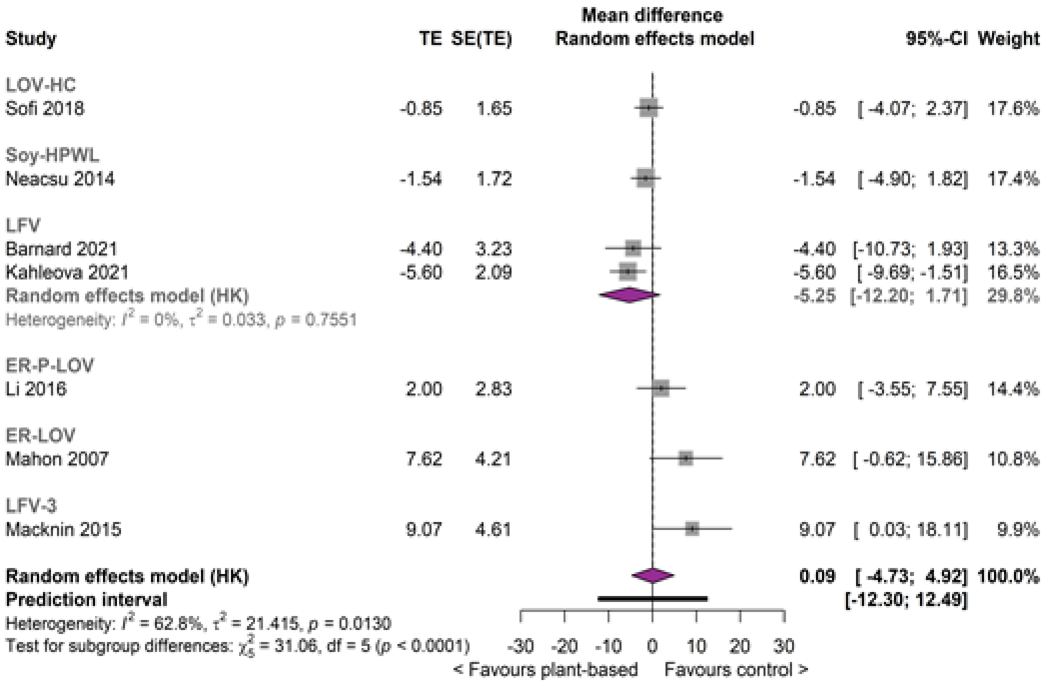

Analysis S5.13 Plant-based diets versus control (omnivorous) diet. Outcome: triglycerides (mg/dl)

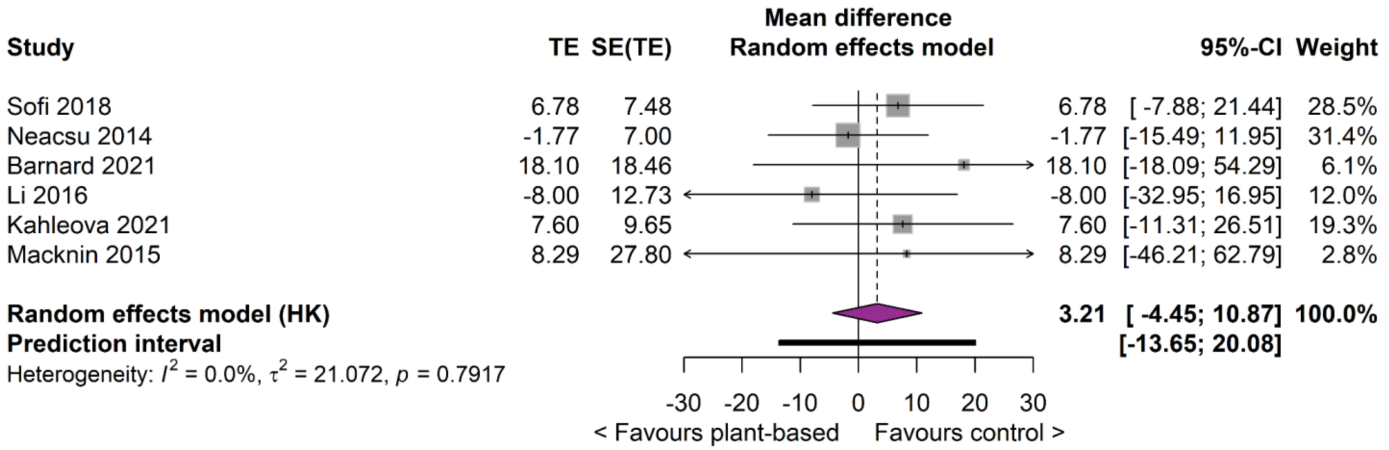

Analysis S5.14 Plant-based diet versus control diet (lacto-ovo vegetarian diet). Outcome: triglycerides (mg/dl)

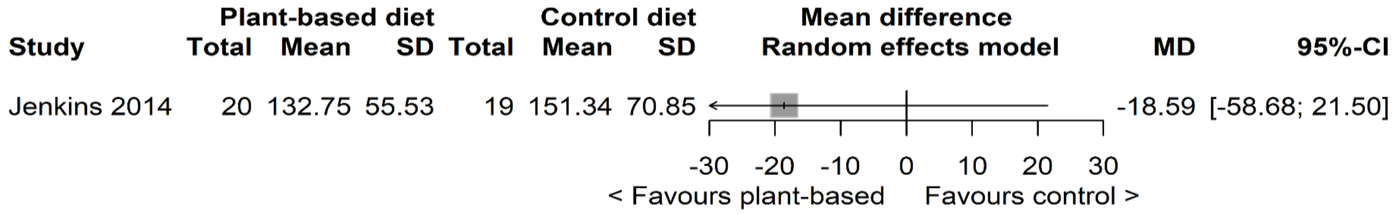

Analysis S5.15 Plant-based diets versus control (omnivorous) diet. Outcome: triglycerides (mg/dl) by diet

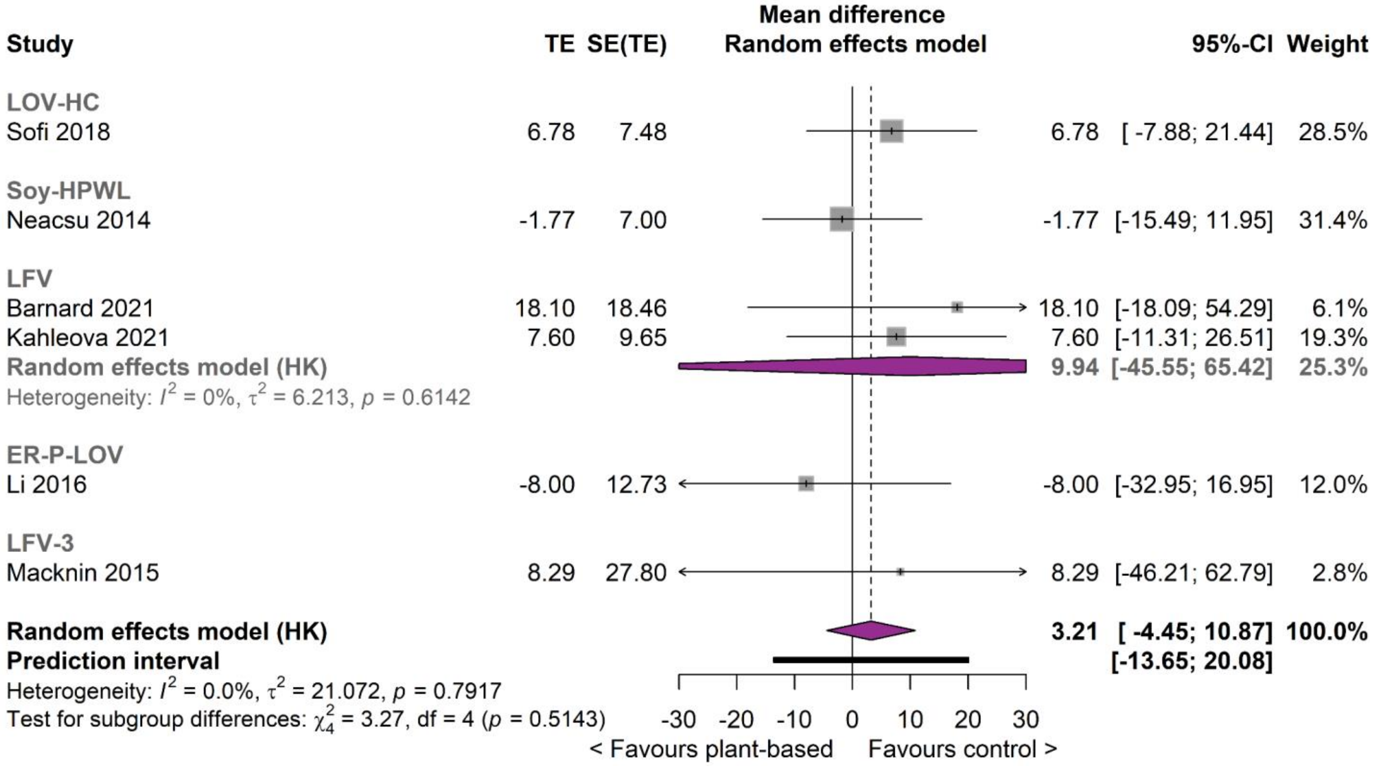

Analysis S5.16 Plant-based diets versus control (omnivorous) diet. Outcome: triglycerides (mg/dl) by time

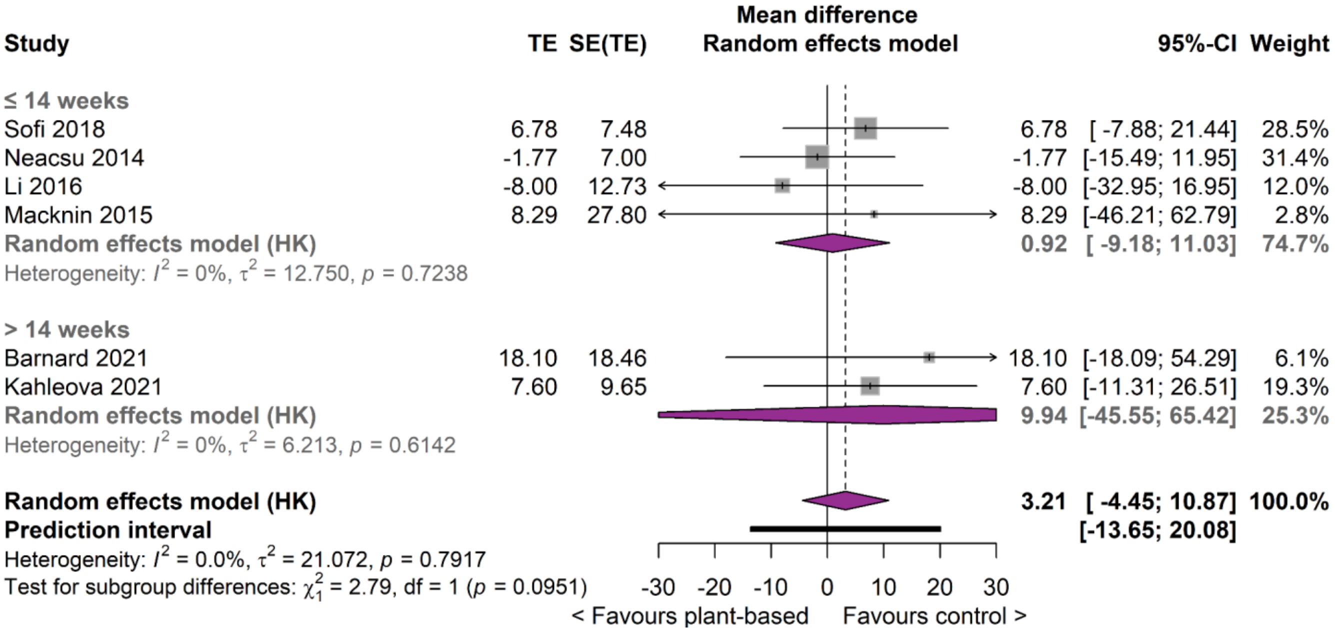

Effect of plant-based diets versus control diet on diabetes-related outcomes (glucose levels, serum insulin, HbA1c, insulin sensitivity)

Analysis S6.1 Plant-based diets versus control (omnivorous) diet. Outcome: glucose (mg/dl)

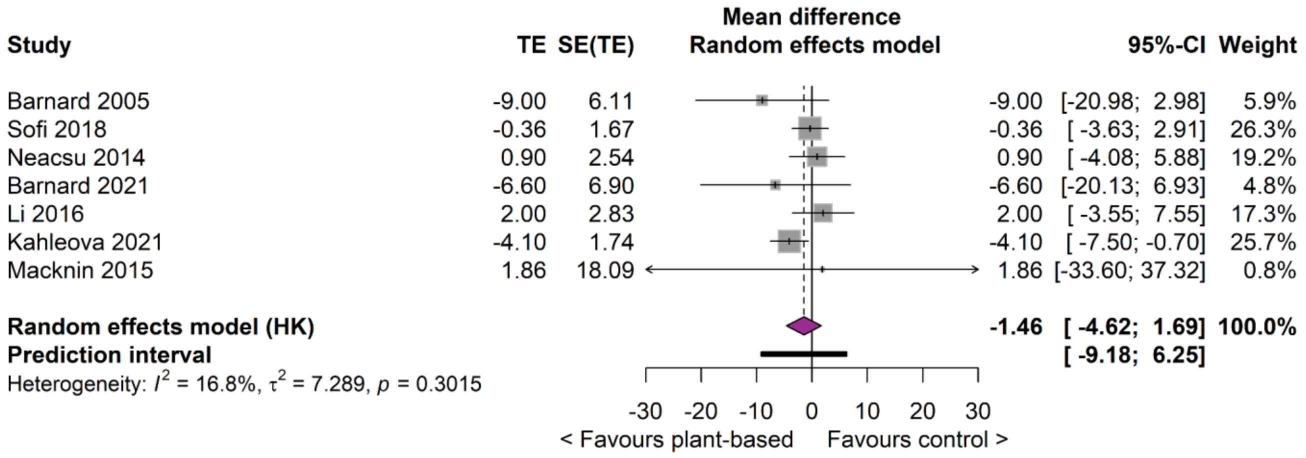

Analysis S6.2 Plant-based diet versus control diet (lacto-ovo vegetarian diet). Outcome: glucose (mg/dl)

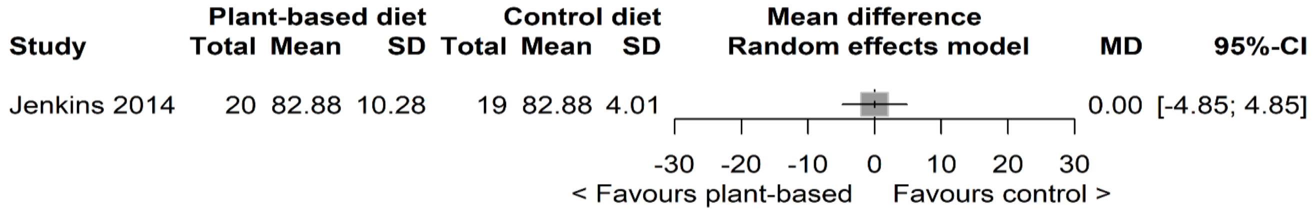

Analysis S6.3 Plant-based diets versus control (omnivorous) diet. Outcome: glucose (mg/dl) by diet

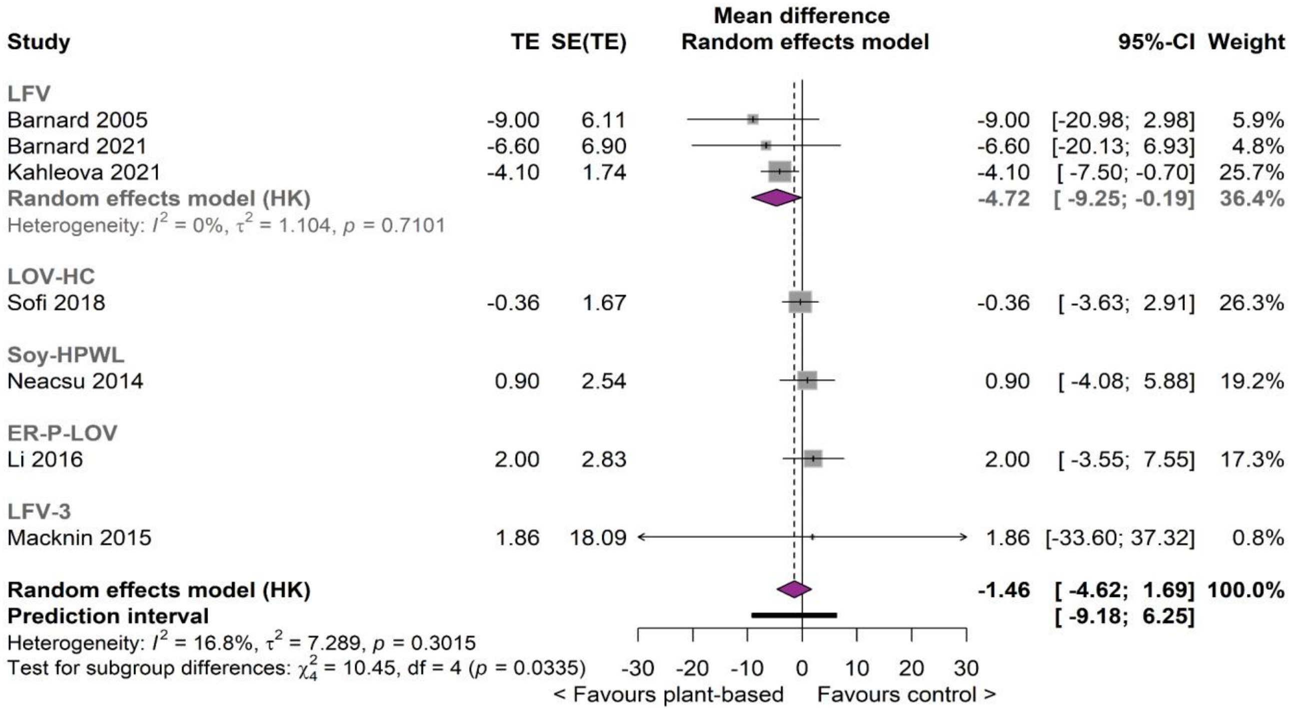

Analysis S6.4 Plant-based diets versus control (omnivorous) diet. Outcome: glucose (mg/dl) by time

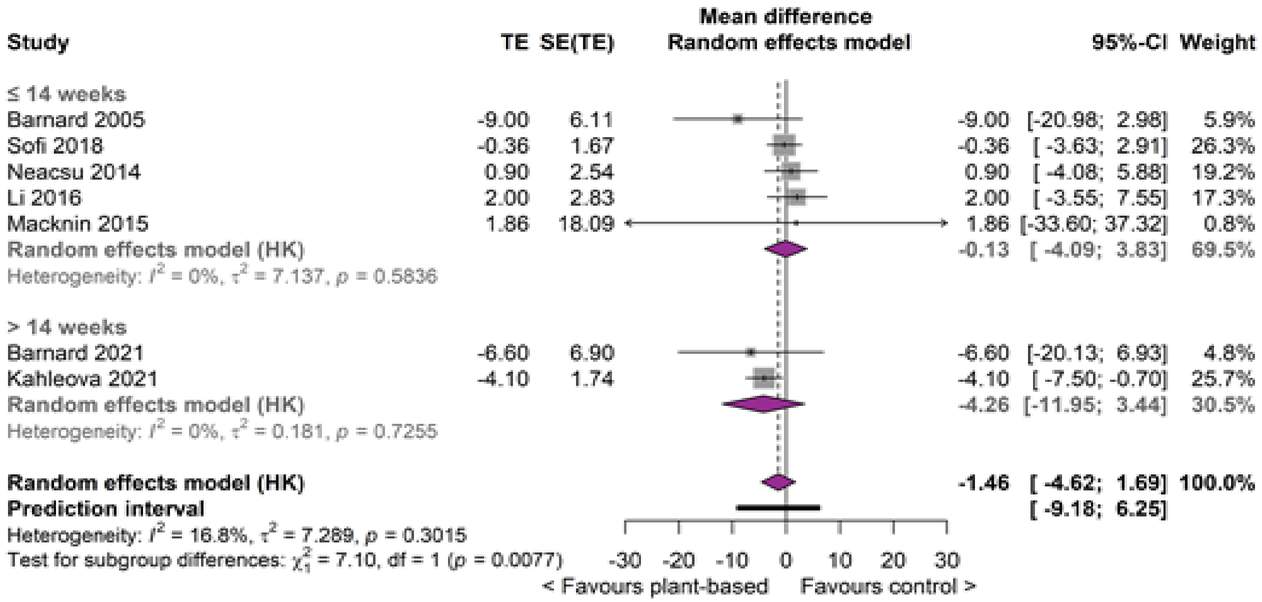

Analysis S6.5 Plant-based diets versus control (omnivorous) diet. Outcome: insulin (μU/mL)

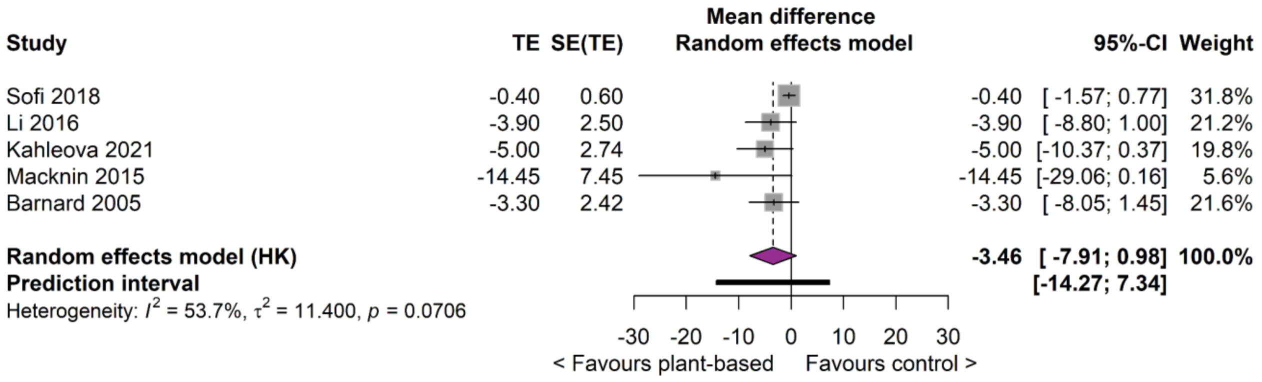

Analysis S6.6 Plant-based diets versus control diet (lacto-ovo vegetarian diet). Outcome: insulin (μU/mL)

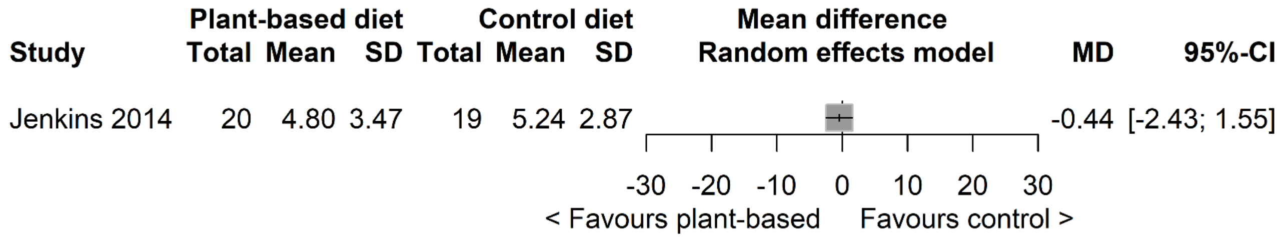

Analysis S6.7 Plant-based diets versus control (omnivorous) diet. Outcome: insulin (μU/mL) by diet

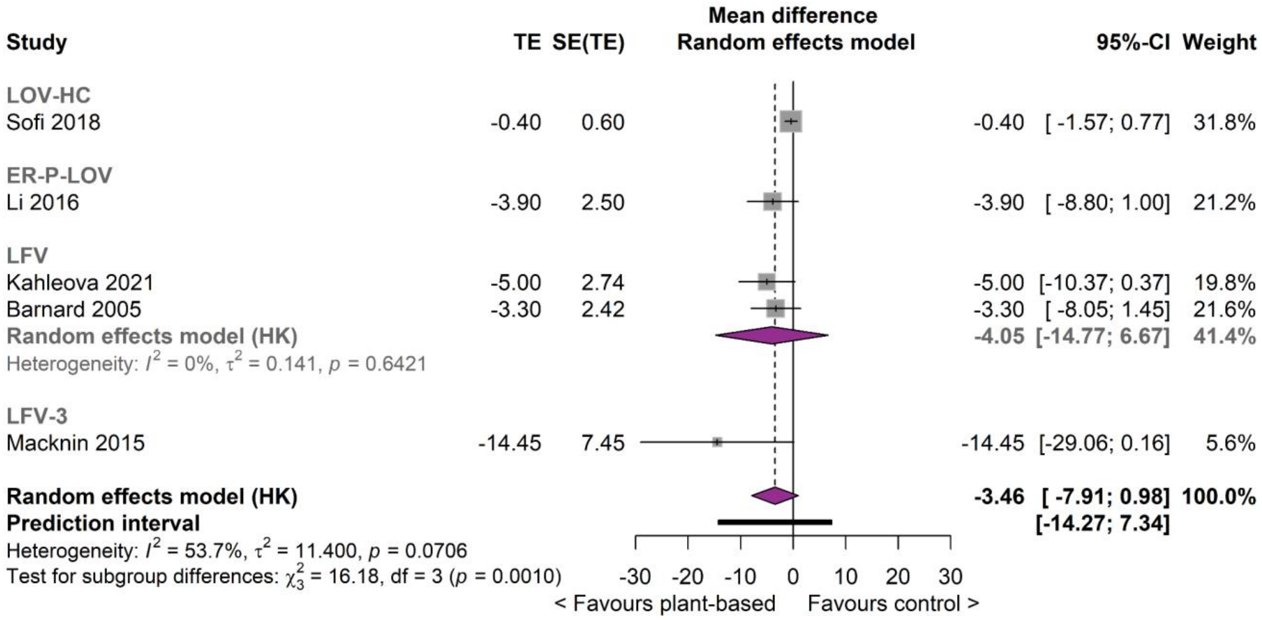

Analysis S6.8 Plant-based diets versus control (omnivorous) diet. Outcome: insulin (μU/mL) by time

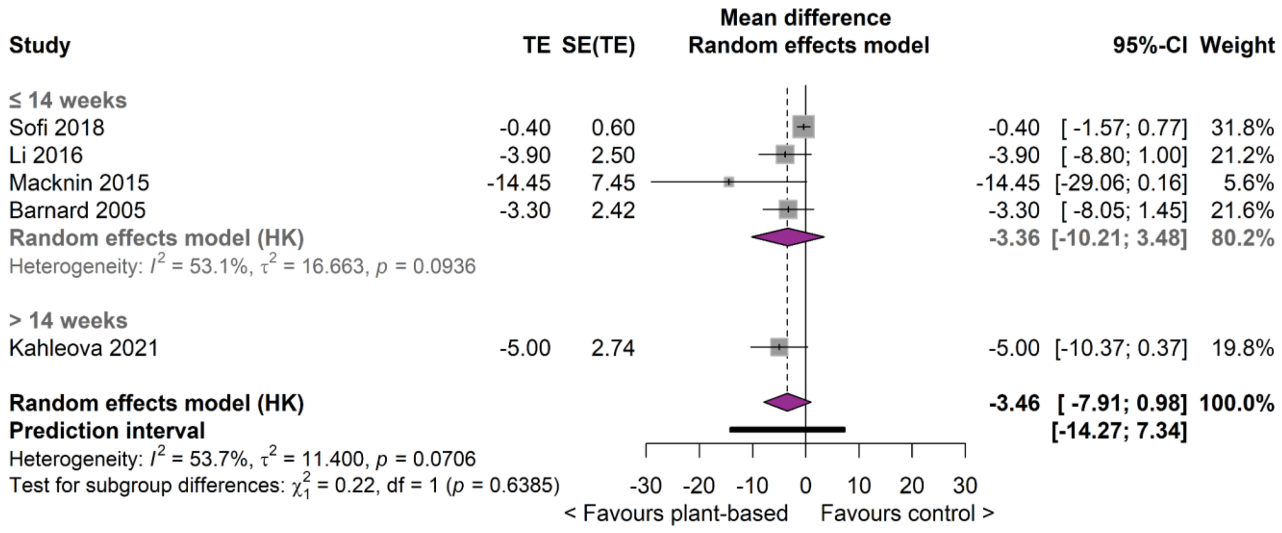

Analysis S6.9 Plant-based diets versus control diet. Outcome: HbA1c (%)

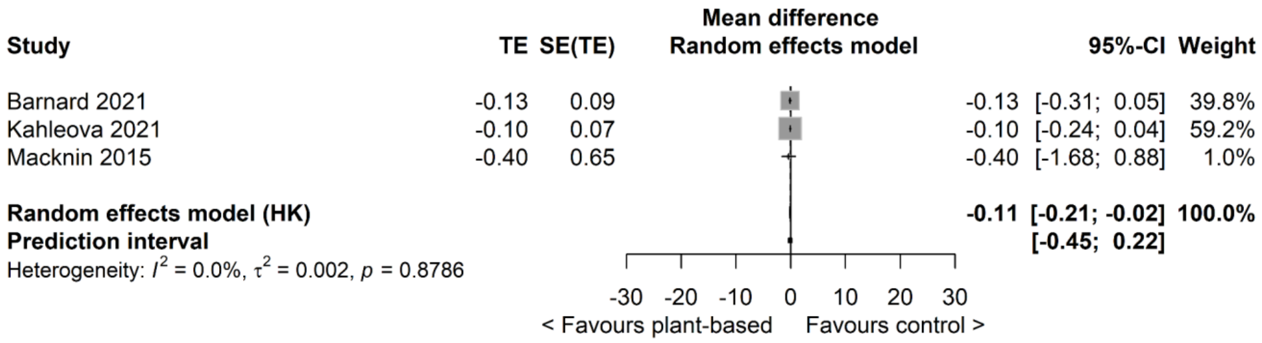

Analysis S6.10 Plant-based diet versus control diet (lacto-ovo vegetarian diet). Outcome: HbA1c (%)

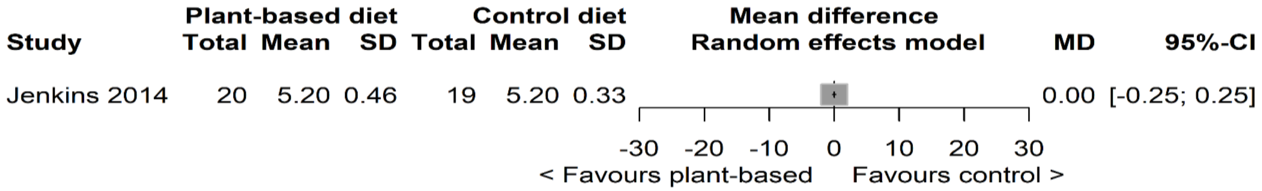

Analysis S6.11 Plant-based diets versus control (omnivorous) diet. Outcome: HbA1c (%) by diet

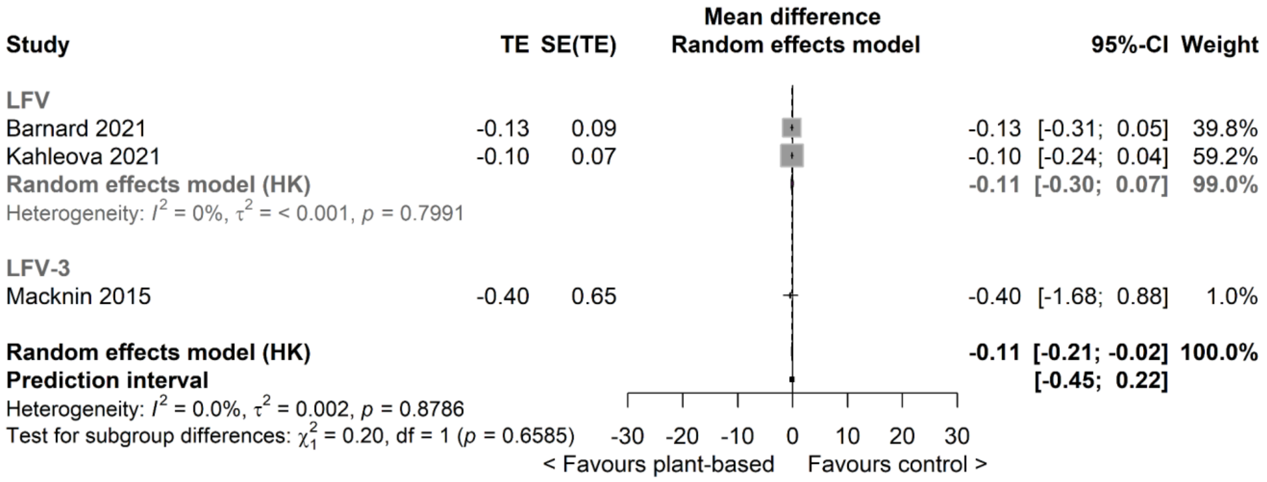

Analysis S6.12 Plant-based diets versus control (omnivorous) diet. Outcome: HbA_{1c} (%) by time

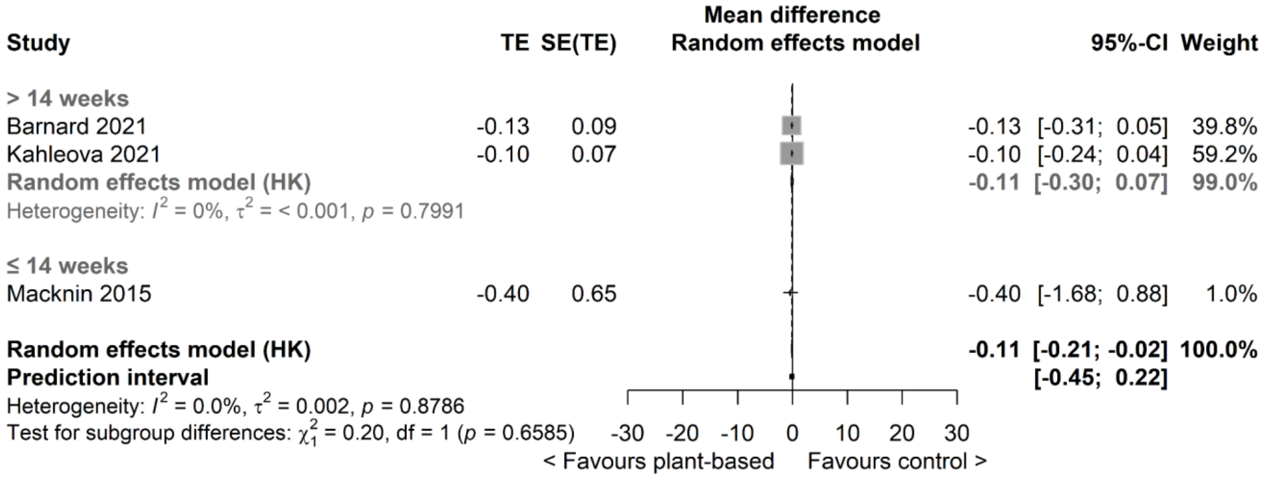

Analysis S6.13 Plant-based diets versus control (omnivorous) diet. Outcome: insulin sensitivity (mg/kg/min)

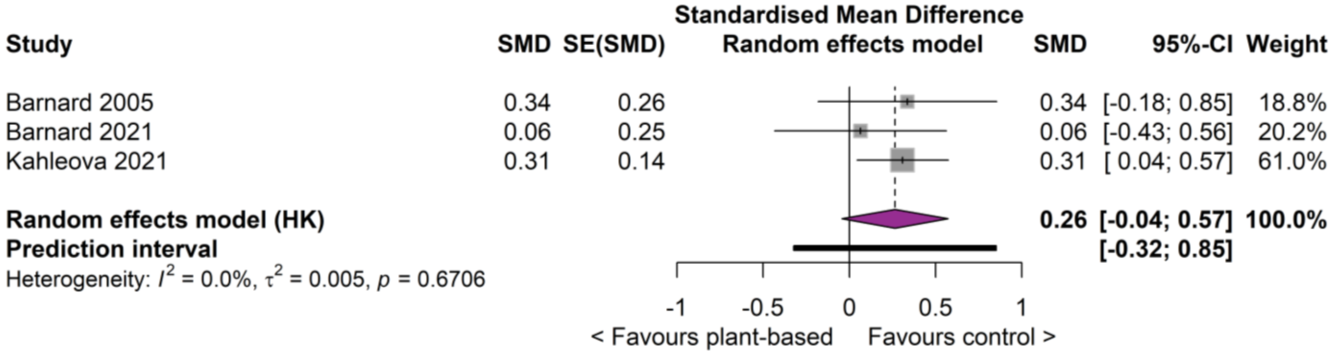

Analysis S6.14 Plant-based diets versus control (omnivorous) diet. Outcome: insulin sensitivity (mg/kg/min) by diet

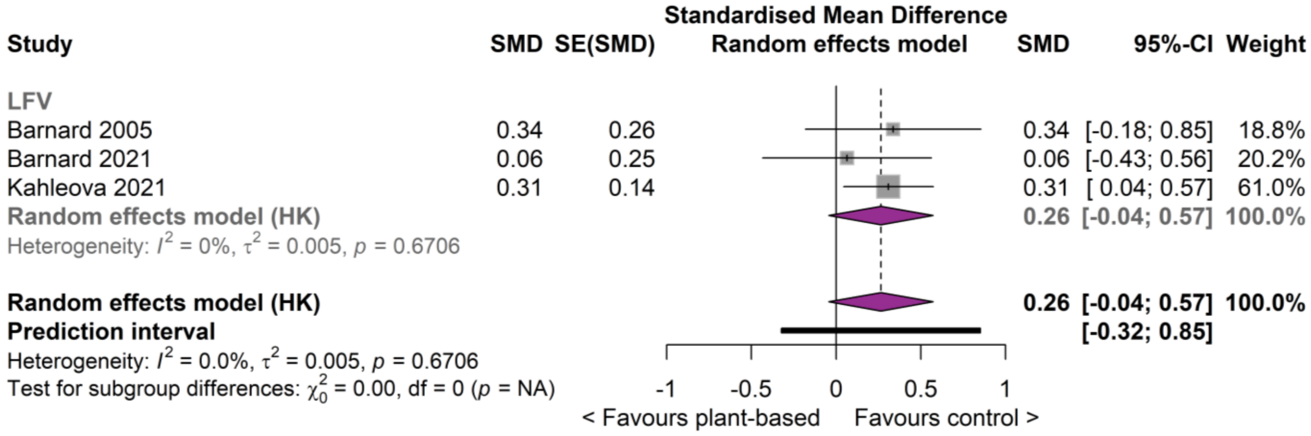

Analysis S6.15 Plant-based diets versus control (omnivorous) diet. Outcome: insulin sensitivity (mg/kg/min) by time

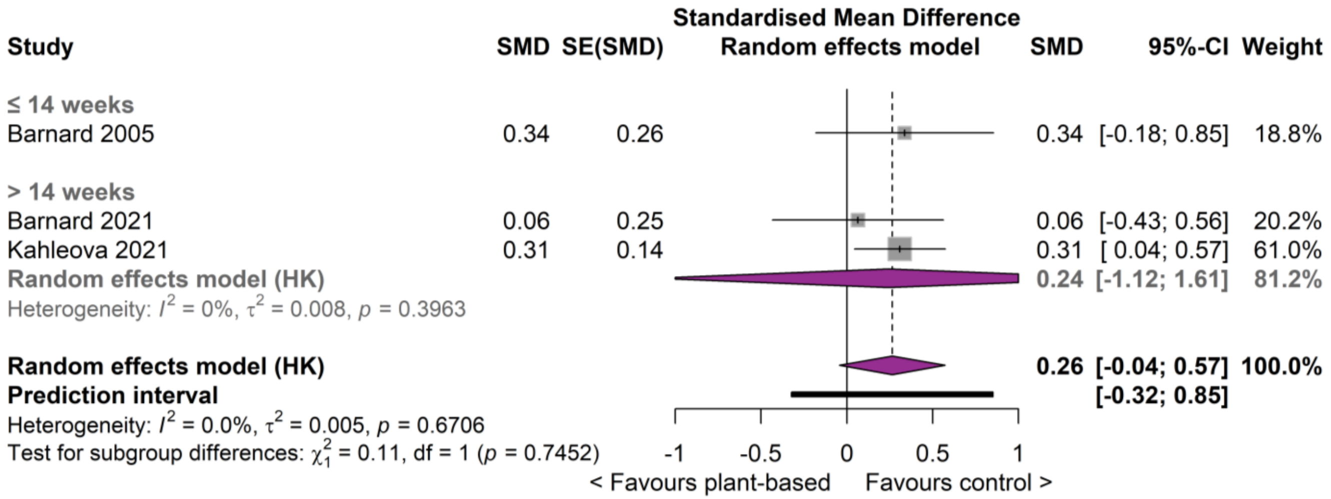

Supplement: Supplementary file 1 [file nutrients-18-01987-s001.zip › Supplementary File S5_Analyses_resubmitted version.pdf]
