# Supplementary material for: Health Effects of Plant-Based Diets in People with Overweight or Obesity: A Systematic Review and Meta-Analysis
Source: Nutrients. 2026 Jun 19;18(12):1987. doi: 10.3390/nu18121987 (PMC13304861; doi:10.3390/nu18121987)
Supplement: Supplementary file 1 [file nutrients-18-01987-s001.zip › Supplementary File S6_Pooled_estimated table.pdf]

Supplementary file S6: Pooled estimated table

Outcome	No. of studies	No. of participants	Statistical method	Effect size
HOMA-IR	8	963	Mean Difference (IV, Random, 95% CI)	-0.41 [-0.74, -0.08]
VAT	5	691	Mean Difference (IV, Random, 95% CI)	-296.12 [-357.89, -234.34]
LEAN mass (kg)	4	387	Mean Difference (IV, Random, 95% CI)	-9.67 [-45.68, 26.34]
Waist (cm)	3	126	Mean Difference (IV, Random, 95% CI)	-2.74 [-9.46, 3.98]
AST (U/L)	2	235	Mean Difference (IV, Random, 95% CI)	-0.58 [-2.03, 0.87]
ALT (U/L)	2	235	Mean Difference (IV, Random, 95% CI)	0.02 [-14.29, 14.34]
APO-A1 (mg/dL)	2	73	Mean Difference (IV, Random, 95% CI)	-6.73 [-70.16, 56.71]
APO-B (mg/dL)	2	73	Mean Difference (IV, Random, 95% CI)	-6.69 [-34.6, 21.22]
hs-CRP (mg/L)	2	67	Mean Difference (IV, Random, 95% CI)	-0.11 [-6.13, 5.91]
IL-6 (pg/mL)	2	235	Mean Difference (IV, Random, 95% CI)	-0.04 [-2.97, 2.9]
Dietary restraint	1	59	Mean Difference (IV, Random, 95% CI)	1 [-0.47, 2.47]
Disinhibition	1	59	Mean Difference (IV, Random, 95% CI)	-0.5 [-2.17, 1.17]

Hunger	1	59	Mean Difference (IV, Random, 95% CI)	0 [-1.28, 1.28]
AE-Decreased energy	1	58	Mean Difference (IV, Random, 95% CI)	– *
AE-Weight gain	1	58	Mean Difference (IV, Random, 95% CI)	–
AE- Dizziness	1	58	Mean Difference (IV, Random, 95% CI)	–
AE-Gassiness	1	58	Mean Difference (IV, Random, 95% CI)	–
AE-Excess hair growth	1	58	Mean Difference (IV, Random, 95% CI)	–
AE-Hair thinning	1	58	Mean Difference (IV, Random, 95% CI)	–
AE-Worse sleep	1	58	Mean Difference (IV, Random, 95% CI)	–
Hip (cm)	1	59	Mean Difference (IV, Random, 95% CI)	2.5 [-0.3, 5.3]
Waist-hip ratio	1	59	Mean Difference (IV, Random, 95% CI)	-0.02 [-2.82, 2.78]
Resting metabolite rate (kcal/d)	1	59	Mean Difference (IV, Random, 95% CI)	-2 [-4.8, 0.8]
Thermic effect of food (kcal/170 min)	1	58	Mean Difference (IV, Random, 95% CI)	0 [-2.8, 2.8]
Adhered to diet (kg)	1	36	Mean Difference (IV, Random, 95% CI)	-5.5 [-15.83, 4.83]

Not adhered to diet (kg)	1	26	Mean Difference (IV, Random, 95% CI)	2.4 [-9.47, 14.27]
VLDL (mg/dL)	1	62	Mean Difference (IV, Random, 95% CI)	-2.7 [-7.48, 2.08]
OGIS (mg/min/m^2)	1	62	Mean Difference (IV, Random, 95% CI)	-23.9 [-59.79, 11.99]
Satiety	1	39	Mean Difference (IV, Random, 95% CI)	-0.2 [-0.59, 0.19]
APO B-AI ratio	1	39	Mean Difference (IV, Random, 95% CI)	-0.04 [-0.14, 0.06]
10-year CVD risk (%)	1	39	Mean Difference (IV, Random, 95% CI)	-2 [-4.5, 0.5]
Gut microbiota diversity	1	115	Mean Difference (IV, Random, 95% CI)	0.1 [-0.04, 0.24]
HOMA-β (%)	1	36	Mean Difference (IV, Random, 95% CI)	36.1 [-16.09, 88.29]
Se creatinine (mg/dL)	1	34	Mean Difference (IV, Random, 95% CI)	0.04 [-0.07, 0.15]
Creatinine clearance rate (mL/min)	1	34	Mean Difference (IV, Random, 95% CI)	4 [-15.6, 23.6]
Glomerular filtration rate (mL/min)	1	34	Mean Difference (IV, Random, 95% CI)	-2 [-13.09, 9.09]
Resting Energy Expenditure (kcal/kg/h)	1	34	Mean Difference (IV, Random, 95% CI)	-0.11 [-0.22, 0]
Fasting BUN (mg/dL)	1	34	Mean Difference (IV, Random, 95% CI)	-0.64 [-3.73, 2.45]

Mid arm (cm)	1	28	Mean Difference (IV, Random, 95% CI)	2.62 [-0.18, 5.42]
MPO (pmol/L)	1	28	Mean Difference (IV, Random, 95% CI)	22.36 [19.56, 25.16]
Food acceptability	1	28	Mean Difference (IV, Random, 95% CI)	0 [0, 0]
Hydroxybutyrate (mmol/L)	1	40	Mean Difference (IV, Random, 95% CI)	-0.12 [-2.92, 2.68]
LDL:HDL ratio	1	40	Mean Difference (IV, Random, 95% CI)	0.2 [-2.6, 3]
Total cholesterol:HDL ratio	1	40	Mean Difference (IV, Random, 95% CI)	0.21 [-2.59, 3.01]
Urea (mmol/L)	1	40	Mean Difference (IV, Random, 95% CI)	0.16 [-2.64, 2.96]
Creatine (μmol/L)	1	40	Mean Difference (IV, Random, 95% CI)	6.74 [3.94, 9.54]
Total bilirubin (μmol/L)	1	40	Mean Difference (IV, Random, 95% CI)	-0.37 [-3.17, 2.43]
Creatinine (g/dL)	1	107	Mean Difference (IV, Random, 95% CI)	0.04 [0, 0.08]
Urea nitrogen (mg/dL)	1	107	Mean Difference (IV, Random, 95% CI)	3 [0.09, 5.91]
BUN (mg/dL)	1	107	Mean Difference (IV, Random, 95% CI)	1.4 [0.05, 2.75]
Uric acid (g/dL)	1	107	Mean Difference (IV, Random, 95% CI)	0.38 [0.02, 0.74]

BUN/creatinine (g/dL)	1	107	Mean Difference (IV, Random, 95% CI)	4.9 [1.08, 8.72]
eGFR	1	107	Mean Difference (IV, Random, 95% CI)	-4.2 [-7.88, -0.52]
Leptin (ng/mL)	1	107	Mean Difference (IV, Random, 95% CI)	-0.48 [-4.51, 3.55]
Adiponectin (μg/mL)	1	107	Mean Difference (IV, Random, 95% CI)	-0.04 [-0.89, 0.81]
L/A ratio	1	107	Mean Difference (IV, Random, 95% CI)	-0.03 [-0.25, 0.19]
Resistin (ng/mL)	1	107	Mean Difference (IV, Random, 95% CI)	0.01 [-1.07, 1.09]
Visfatin (ng/mL)	1	107	Mean Difference (IV, Random, 95% CI)	0.07 [-0.2, 0.34]
PAI-1 (ng/mL)	1	107	Mean Difference (IV, Random, 95% CI)	-0.73 [-3.71, 2.25]
Ghrelin (pg/mL)	1	107	Mean Difference (IV, Random, 95% CI)	1.3 [-45.83, 48.43]
WBC (*103/mm3)	1	207	Mean Difference (IV, Random, 95% CI)	0.03 [-0.35, 0.41]
RBC (*103/mm3)	1	207	Mean Difference (IV, Random, 95% CI)	0.05 [-0.06, 0.16]
Hemoglobin (g/dL)	1	207	Mean Difference (IV, Random, 95% CI)	0.14 [-0.2, 0.48]
Hematocrit (%)	1	207	Mean Difference (IV, Random, 95% CI)	0.29 [-0.67, 1.25]

Folate (ng/mL)	1	207	Mean Difference (IV, Random, 95% CI)	0.18 [-0.79, 1.15]
Vitamin B12 (pg/mL)	1	207	Mean Difference (IV, Random, 95% CI)	28.53 [-2.97, 60.03]
Ferritin (ng/mL)	1	207	Mean Difference (IV, Random, 95% CI)	2.83 [-12.35, 18.01]
Iron (μg/dL)	1	207	Mean Difference (IV, Random, 95% CI)	0.47 [-7.25, 8.19]
Sodium (mEq/L)	1	207	Mean Difference (IV, Random, 95% CI)	-0.18 [-0.63, 0.27]
Potassium (mEq/L)	1	207	Mean Difference (IV, Random, 95% CI)	0.02 [-0.05, 0.09]
Calcium (mg/dL)	1	207	Mean Difference (IV, Random, 95% CI)	0 [-0.12, 0.12]
Magnesium (mg/dL)	1	207	Mean Difference (IV, Random, 95% CI)	0 [-0.05, 0.05]
γ-GT (U/L)	1	207	Mean Difference (IV, Random, 95% CI)	-0.1 [-3.87, 3.67]
TBARS (pg/mL)	1	207	Mean Difference (IV, Random, 95% CI)	0.02 [-0.3, 0.34]
TAC (μmol/mL)	1	207	Mean Difference (IV, Random, 95% CI)	0.18 [-0.78, 1.14]
L-derived ROS (RFU)	1	207	Mean Difference (IV, Random, 95% CI)	18.4 [-50.76, 87.56]
M-derived ROS (RFU)	1	207	Mean Difference (IV, Random, 95% CI)	-16.4 [-131.24, 98.44]

G-derived ROS (RFU)	1	207	Mean Difference (IV, Random, 95% CI)	-63.1 [-230.17, 103.97]
IL-1 ra (pg/mL)	1	207	Mean Difference (IV, Random, 95% CI)	0.37 [-1.96, 2.7]
IL-4 (pg/mL)	1	207	Mean Difference (IV, Random, 95% CI)	0 [-0.05, 0.05]
IL-8 (pg/mL)	1	207	Mean Difference (IV, Random, 95% CI)	0.15 [-0.79, 1.09]
IL10 (pg/mL)	1	207	Mean Difference (IV, Random, 95% CI)	-0.33 [-0.97, 0.31]
IL-12 (pg/mL)	1	207	Mean Difference (IV, Random, 95% CI)	-1.08 [-4.08, 1.92]
IL17 (pg/mL)	1	207	Mean Difference (IV, Random, 95% CI)	-1.58 [-3.01, -0.15]
MCP-1 (pg/mL)	1	207	Mean Difference (IV, Random, 95% CI)	-1.15 [-4.08, 1.78]
MIP-1β (pg/ml)	1	207	Mean Difference (IV, Random, 95% CI)	0.36 [-5.79, 6.51]
VEGF (pg/mL)	1	207	Mean Difference (IV, Random, 95% CI)	0.86 [-7.62, 9.34]
TNF-α (pg/mL)	1	207	Mean Difference (IV, Random, 95% CI)	-0.64 [-1.72, 0.44]
IP-10 (pg/mL)	1	207	Mean Difference (IV, Random, 95% CI)	12.79 [-47.45, 73.03]
IFN-γ (pg/mL)	1	207	Mean Difference (IV, Random, 95% CI)	0.56 [-0.43, 1.55]

Hepatocellular lipids (%)	1	223	Mean Difference (IV, Random, 95% CI)	1.2 [1.02, 1.38]
Intramyocellular lipids (%)	1	223	Mean Difference (IV, Random, 95% CI)	0.2 [0.02, 0.38]
Total bone area (g)	1	43	Mean Difference (IV, Random, 95% CI)	52.41 [49.61, 55.22]
Total bone mineral density (g/cm^2)	1	43	Mean Difference (IV, Random, 95% CI)	0.02 [-2.78, 2.82]
Total bone mineral content (cm^2)	1	43	Mean Difference (IV, Random, 95% CI)	109.1 [106.3, 111.91]
C-reactive protein (mg/L)	1	43	Mean Difference (IV, Random, 95% CI)	-0.49 [-3.29, 2.31]
CHOL:HDL	1	43	Mean Difference (IV, Random, 95% CI)	-0.09 [-2.9, 2.71]

* Results were not suitable for analysis.

Abbreviations: HOMA-IR: Homeostatic Model Assessment for Insulin Resistance; VAT: visceral adipose tissue; AST: aspartate aminotransferase; ALP: Alkaline phosphatase; APO: apolipoprotein; hs-CRP: high-sensitivity C-reactive protein; IL: interleukin; AE: adverse effect; VLDL: very-low-density-lipoprotein; OGIS: oral glucose insulin sensitivity; CVD: cardiovascular disease; fasting BUN: fasting blood urea nitrogen; MPO: myeloperoxidase; LDL:HDL ratio: low-density lipoprotein:high density lipoprotein ratio; L/A ratio: leptin/adiponectin ratio; PAI: Plasminogen activator inhibitor; WBC: white blood cell; RBC: red blood cell; γ-GT: gamma-glutamyl-transferase; TBARS: Thiobarbituric acid reactive substances; TAC: total antioxidant capacity; ROS: reactive oxygen species; MCP: monocyte chemoattractant protein; MIP: macrophage inflammatory protein; VEGF: vascular endothelial growth factor; TNF-α: tumour necrosis factor-alpha; IFN-γ: interferon gamma; CHOL: cholesterol
