# Supplementary material for: Health Effects of Plant-Based Diets in People with Overweight or Obesity: A Systematic Review and Meta-Analysis
Source: Nutrients. 2026 Jun 19;18(12):1987. doi: 10.3390/nu18121987 (PMC13304861; doi:10.3390/nu18121987)

## Supplementary file S7

## Risk of bias assessment

**Figure 1. Risk of bias summary: review authors' judgements about each risk of bias item presented as percentage across all parallel design included RCTs**

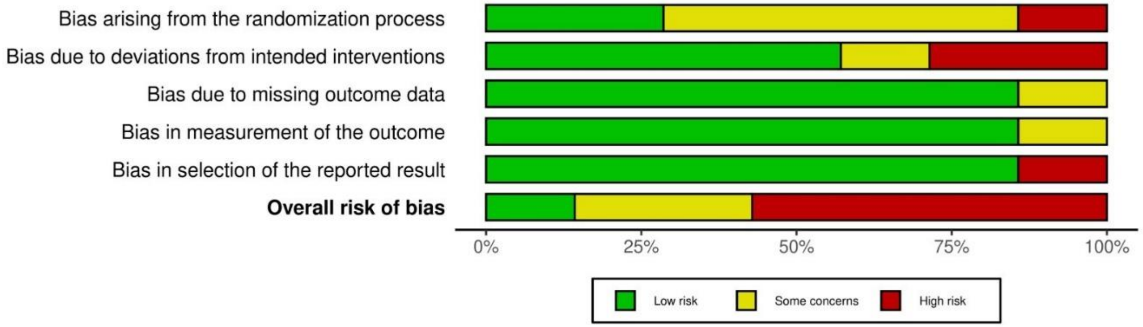


**Figure 2. Bias risk graph: the review authors' judgements on each bias risk element are shown for all included parallel RCTs**

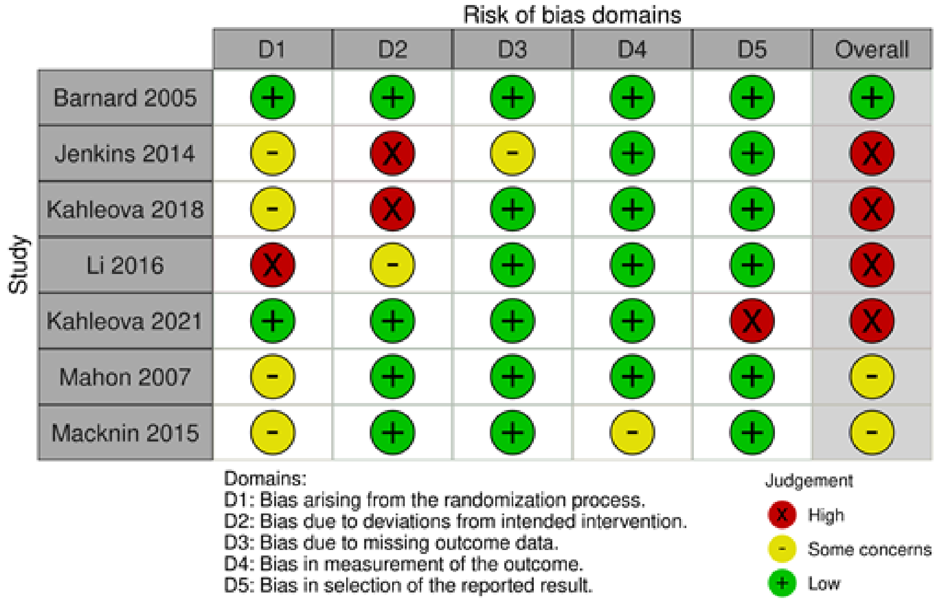


**Figure 3. Bias risk graph: the review authors' judgements on each bias risk element are shown for all included crossover RCTs**

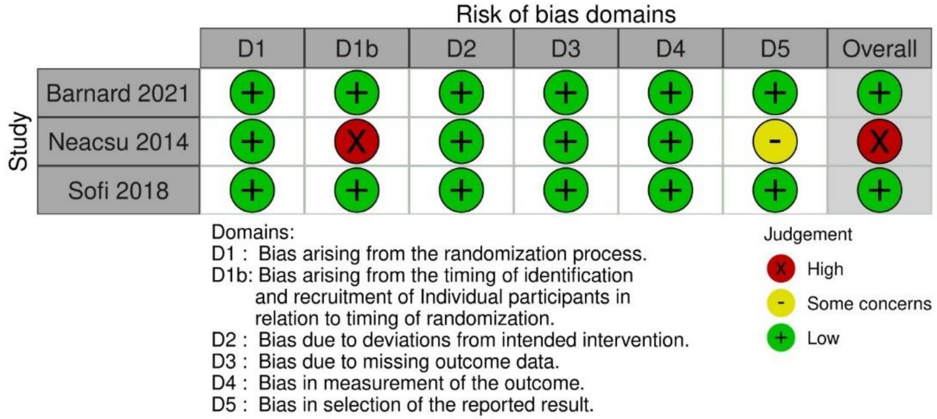

Supplement: Supplementary file 1 [file nutrients-18-01987-s001.zip › Supplementary File S7_RoB.pdf]
