# Supplementary material for: Health Effects of Plant-Based Diets in People with Overweight or Obesity: A Systematic Review and Meta-Analysis
Source: Nutrients. 2026 Jun 19;18(12):1987. doi: 10.3390/nu18121987 (PMC13304861; doi:10.3390/nu18121987)
Supplement: Supplementary file 1 [file nutrients-18-01987-s001.zip › Supplementary file S8_GRADE_resubmitted version.pdf]

# Supplementary file S9: GRADE Assessment

| Certainty assessment | | | | | | | № of patients | | Effect | | Certainty | Importance |
|---|---|---|---|---|---|---|---|---|---|---|---|---|
| № of studies | Study design | Risk of bias | Inconsistency | Indirectness | Imprecision | Other considerations | plant-based diet | omnivorous diet | Relative (95% CI) | Absolute (95% CI) | | |
| Body weight (follow-up: range 4 to 16 weeks; assessed with: kg) | | | | | | | | | | | | |
| 8 [1-8] | randomised trials | serious[a] | not serious | not serious | serious[b] | none | 392 | 398 | - | MD **2.81 kg lower** (5.63 lower to 0.02 higher) | ⨁⨁◯◯ Low[a,b] | CRITICAL |
| BMI (follow-up: range 9 to 16 weeks; assessed with: kg/m2) | | | | | | | | | | | | |
| 7 [1-4,6-8] | randomised trials | serious[c] | serious[d] | not serious | serious[b] | none | 378 | 384 | - | MD **1.15 kg/m2 lower** (2.17 lower to 0.13 lower) | ⨁◯◯◯ Very low[b,c,d] | CRITICAL |
| Systolic blood pressure (follow-up: range 4 to 16 weeks; assessed with: mmHg) | | | | | | | | | | | | |
| 3 [3,5,7] | randomised trials | serious[e] | not serious | not serious | very serious[f] | none | 93 | 93 | - | MD **3.32 Hgmm higher** (0.38 lower to 7.03 higher) | ⨁◯◯◯ Very low[e,f] | CRITICAL |
| Diastolic blood pressure (follow-up: range 4 to 16 weeks; assessed with: mmHg) | | | | | | | | | | | | |
| 3 [3,5,7] | randomised trials | serious[e] | serious[g] | not serious | very serious[f] | none | 93 | 93 | - | MD **2.07 Hgmm higher** (0.68 lower to 4.83 higher) | ⨁◯◯◯ Very low[e,f,g] | CRITICAL |
| Serum glucose (follow-up: range 4 to 16 weeks; assessed with: mg/dL) | | | | | | | | | | | | |

| Certainty assessment | | | | | | | № of patients | | Effect | | Certainty | Importance |
|---|---|---|---|---|---|---|---|---|---|---|---|---|
| № of studies | Study design | Risk of bias | Inconsistency | Indirectness | Imprecision | Other considerations | plant-based diet | omnivorous diet | Relative (95% CI) | Absolute (95% CI) | | |
| 7 [1-3,5-7,9] | randomised trials | serious[c] | not serious | not serious | serious[b] | none | 357 | 346 | - | MD **1.46 mg/dL lower** (4.62 lower to 1.69 higher) | ⨁⨁◯◯ Low[b,c] | CRITICAL |
| Serum insulin (follow-up: range 4 to 16 weeks; assessed with: mU/L) | | | | | | | | | | | | |
| 5 [1-3,5,6] | randomised trials | serious[h] | not serious | not serious | serious[b] | none | 267 | 256 | - | MD **3.46 mU/L lower** (7.91 lower to 0.98 higher) | ⨁⨁◯◯ Low[b,h] | CRITICAL |
| Total cholesterol (follow-up: range 4 to 16 weeks; assessed with: mg/dL) | | | | | | | | | | | | |
| 7 [2-7,9] | randomised trials | serious[i] | serious[j] | not serious | serious[b] | none | 348 | 351 | - | MD **4.31 mg/dL lower** (18.88 lower to 10.27 higher) | ⨁◯◯◯ Very low[b,i,j] | IMPORTANT |
| Triglycerides (follow-up: range 4 to 16 weeks; assessed with: mg/dL) | | | | | | | | | | | | |
| 6 [2,3,5-7,9] | randomised trials | serious[k] | not serious | not serious | serious[b] | none | 334 | 322 | - | MD **3.21 mg/dL higher** (4.45 lower to 10.87 higher) | ⨁⨁◯◯ Low[b,k] | IMPORTANT |
| LDL cholesterol (follow-up: range 4 to 16 weeks; assessed with: mg/dL) | | | | | | | | | | | | |
| 7 [2,3,5-7,9] | randomised trials | serious[i] | not serious | not serious | serious[b] | none | 348 | 351 | - | MD **7.29 mg/dL lower** (13.3 lower to 1.28 lower) | ⨁⨁◯◯ Low[b,i] | IMPORTANT |
| HDL cholesterol (follow-up: range 4 to 16 weeks; assessed with: mg/dL) | | | | | | | | | | | | |

| Certainty assessment | | | | | | | № of patients | | Effect | | Certainty | Importance |
|---|---|---|---|---|---|---|---|---|---|---|---|---|
| № of studies | Study design | Risk of bias | Inconsistency | Indirectness | Imprecision | Other considerations | plant-based diet | omnivorous diet | Relative (95% CI) | Absolute (95% CI) | | |
| 7 [2-7,9] | randomised trials | serious[i] | serious[l] | not serious | serious[b] | none | 348 | 351 | - | MD **0.09 mg/dL higher** (4.73 lower to 4.92 higher) | ⨁◯◯◯ Very low[b,i,l] | IMPORTANT |
| Body fat mass in kg (follow-up: range 9 weeks to 16 weeks; assessed with: kg) | | | | | | | | | | | | |
| 4 [4,6-8] | randomised trials | serious[m] | serious[n] | not serious | very serious[f] | none | 228 | 234 | - | MD **1.37 kg lower** (5.98 lower to 3.24 higher) | ⨁◯◯◯ Very low[f,m,n] | IMPORTANT |
| Body fat mass in mass % (follow-up: range 9 to 14 weeks; assessed with: %) | | | | | | | | | | | | |
| 3 [1,3,4] | randomised trials | serious[e] | not serious | not serious | very serious[f] | none | 60 | 76 | - | MD **0.57 % higher** (2.1 lower to 3.24 higher) | ⨁◯◯◯ Very low[e,f] | IMPORTANT |
| HbA1c (follow-up: range 4 to 16 weeks; assessed with: %) | | | | | | | | | | | | |
| 3 [5-7] | randomised trials | serious[e] | not serious | not serious | very serious[f] | none | 193 | 182 | - | MD **0.11 % lower** (0.21 lower to 0.02 lower) | ⨁◯◯◯ Very low[f,m] | IMPORTANT |
| Insulin sensitivity (follow-up: range 14 to 16 weeks; assessed with: mg/kg/min) | | | | | | | | | | | | |
| 3 [1,6,7] | randomised trials | serious[o] | not serious | not serious | very serious[f] | none | 208 | 198 | - | MD **~~0.55~~ 0.26 mg/kg/min higher** (~~0.09~~ 0.04 higher to ~~1.01~~ 0.57 higher) | ⨁◯◯◯ Very low[f,o] | IMPORTANT |

**CI:** confidence interval; **MD:** mean difference

## Explanations

a. Downgraded by one lever for risk of bias, as three studies were rated with high RoB, two with some concerns, and three with low RoB.

b. Downgraded by one level for imprecision. The median sample size was low (<100 participants) and the number of included studies was moderate (5 to 10 studies)

c. Downgraded by one level for risk of bias, as 3 studies out of the included 7 were rated high RoB, and one was rated with some concern for RoB.

d. Downgraded by one level for inconsistency since I2 was 85%, p-value for heterogeneity was <0.0001. Sub-group analyses did not fully explain heterogeneity.

e. Downgraded by one lever for risk of bias, as one study was rated with high RoB, one with some concerns, and only one with low RoB.

f. Downgraded by two levels for imprecision. The median sample size was low (<100 participants) and the number of included studies was low (<5 studies)

g. Downgraded by one level for inconsistency since I2 was 53%, p-value for heterogeneity was <0.05. Sub-group analyses did not fully explain heterogeneity.

h. Downgraded by one lever for risk of bias, as two studies were rated with high RoB, one with some concerns, and two with low RoB.

i. Downgraded by one lever for risk of bias, as three studies were rated with high RoB, two with some concerns, and two with low RoB.

j. Downgraded by one level for inconsistency. Statistical heterogeneity was moderate (I2 was 52%), and was only partially explained by the sub-group analyses.

k. Downgraded by one lever for risk of bias, as three studies were rated with high RoB, one with some concerns, and two with low RoB.

l. Downgraded by one level for inconsistency since I2 was 62%. Sub-group analyses did not fully explain heterogeneity.

m. Downgraded by one lever for risk of bias, as two studies were rated with high RoB, one with some concerns, and only one with low RoB.

n. Downgraded by one level for inconsistency since I2 was 74%, p-value for heterogeneity was <0.05. Sub-group analyses did not fully explain heterogeneity.

o. Downgraded by one level for risk of bias, as one study out of the included 3 was rated high RoB.

## References


1. Barnard, N.D.; Scialli, A.R.; Turner-McGrievy, G.; Lanou, A.J.; Glass, J. The effects of a low-fat, plant-based dietary intervention on body weight, metabolism, and insulin sensitivity. *American Journal of Medicine* **2005**, *118*, 991-997. https://doi.org/10.1016/j.amjmed.2005.03.039
2. Sofi, F.; Dinu, M.; Pagliai, G.; Cesari, F.; Gori, A.M.; Sereni, A.; Becatti, M.; Fiorillo, C.; Marcucci, R.; Casini, A. Low-Calorie Vegetarian Versus Mediterranean Diets for Reducing Body Weight and Improving Cardiovascular Risk Profile: CARDIVEG Study (Cardiovascular Prevention With Vegetarian Diet). *Circulation* **2018**, *137*, 1103-1113, https://dx.doi.org/10.1161/CIRCULATIONAHA.117.030088.
3. Li, J.; Armstrong, C.L.; Campbell, W.W. Effects of Dietary Protein Source and Quantity during Weight Loss on Appetite, Energy Expenditure, and Cardio-Metabolic Responses. *Nutrients* **2016**, *8*, 63, https://dx.doi.org/10.3390/nu8020063.
4. Mahon, A.K.; Flynn, M.G.; Stewart, L.K.; McFarlin, B.K.; Iglay, H.B.; Mattes, R.D.; Lyle, R.M.; Considine, R.V.; Campbell, W.W. Protein intake during energy restriction: effects on body composition and markers of metabolic and cardiovascular health in postmenopausal women. *Journal of the American College of Nutrition* **2007**, *26*, 182-189. https://doi.org/10.1080/07315724.2007.10719600
5. Macknin, M.; Kong, T.; Weier, A.; Worley, S.; Tang, A.S.; Alkhouri, N.; Golubic, M. Plant-based, no-added-fat or American heart association diets: Impact on cardiovascular risk in obese children with hypercholesterolemia and their parents. *Journal of Pediatrics* **2015**, *166*, 953-959.e953, https://doi.org/10.1016/j.jpeds.2014.12.058

6. Kahleova, H.; McCann, J.; Alwarith, J.; Rembert, E.; Tura, A.; Holubkov, R.; Barnard, N.D. A plant-based diet in overweight adults in a 16-week randomized clinical trial: The role of dietary acid load. *Clin Nutr ESPEN* **2021**, *44*, 150-158, https://doi.org/10.1016/j.clnesp.2021.05.015
7. Barnard, N.D.; Alwarith, J.; Rembert, E.; Brandon, L.; Nguyen, M.; Goergen, A.; Horne, T.; do Nascimento, G.F.; Lakkadi, K.; Tura, A.; et al. A Mediterranean Diet and Low-Fat Vegan Diet to Improve Body Weight and Cardiometabolic Risk Factors: A Randomized, Cross-over Trial. *J Am Coll Nutr* **2021**, 1-13, https://doi.org/10.1080/07315724.2020.1869625
8. Kahleova, H.; Dort, S.; Holubkov, R.; Barnard, N.D. A Plant-Based High-Carbohydrate, Low-Fat Diet in Overweight Individuals in a 16-Week Randomized Clinical Trial: The Role of Carbohydrates. *Nutrients* **2018**, *10*, 14, https://dx.doi.org/10.3390/nu10091302.
9. Neacsu, M.; Fyfe, C.; Horgan, G.; Johnstone, A.M. Appetite control and biomarkers of satiety with vegetarian (soy) and meat-based high-protein diets for weight loss in obese men: a randomized crossover trial. *American Journal of Clinical Nutrition* **2014**, *100*, 548-558, https://dx.doi.org/10.3945/ajcn.113.077503.
